# Supplementary material for: Creating synthetic populations in transplantation: A Bayesian approach enabling simulation without registry re-sampling
Source: PLoS One. 2024 Mar 21;19(3):e0296839. doi: 10.1371/journal.pone.0296839 (PMC10956776; doi:10.1371/journal.pone.0296839)
Supplement: S1 Text — (DOCX) [file pone.0296839.s001.docx]

Appendix

S1 Text – Details of Bayesian Network

Bayesian networks are graph-based representations of conditionality among a set of variables.^1^ In these graphs, conditionality between variables (boxes or “nodes”) is represented by directional links (arrows). For example, a simple, four-variable Bayesian network might be represented by a set of relationship depicted in **Figure A**:

**Figure A** – Illustrative directed acyclic graph (DAG)


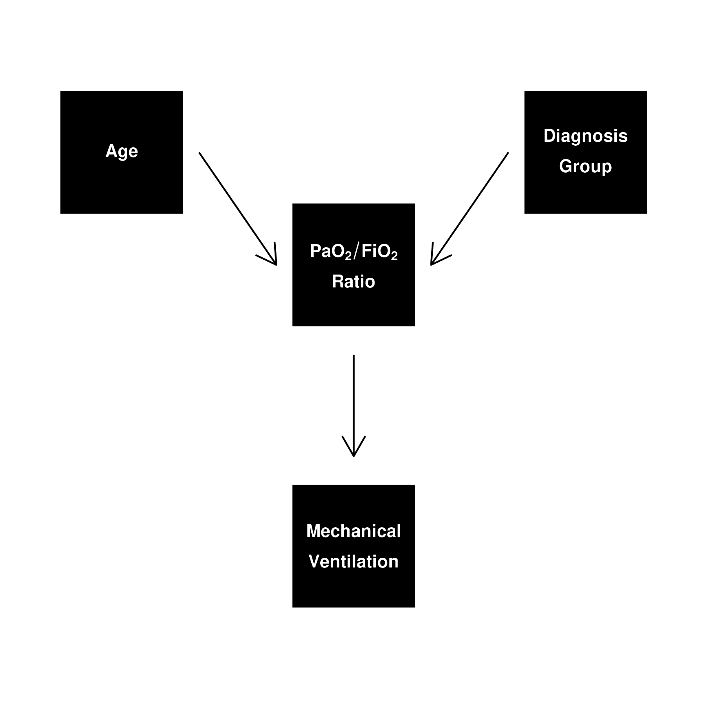


In this simplified network, PaO2/FiO2 (P/F) ratio is the “descendent” of the “parent” nodes age and diagnosis group, while mechanical ventilation is the descendent of P/F ratio. Assuming these conditional relationships are correctly specified, then whether a candidate is on mechanical ventilation could be predicted solely by observing their P/F ratio; in other words, mechanical ventilation is conditionally independent from age and diagnosis group when the P/F ratio is known. Bayesian networks can be interpreted as causal networks when the links between nodes represent assumed or hypothesized causal relationships.^2,3^ A key characteristic of Bayesian networks is that they are “acyclic”, meaning that a given node does not directly or indirectly inform its parents. Hence, graphical depictions of Bayesian networks like the one above are known as directed acyclic graphs (DAGs).

Probability theory can be applied to prove an important property in Bayesian networks: The joint probability distribution of all the variables in a path is equal to the product of conditional distributions for each variable in that path, given each variables’ respective parents.^1^ To illustrate using our simplified example, this implies:

Pr(age, diagnosis group, P/F ratio, mechanical ventilation) =

Pr(age) × Pr(diagnosis group) × Pr(P/F ratio | age, diagnosis group) × Pr(mechanical ventilation | P/F ratio).

where “,” can be interpreted as “and” and “|” can be interpreted as “given” (or “conditioned upon”). In other words, the probability (Pr) of observing a specific combination of age, diagnosis group, P/F ratio, and mechanical ventilation status in a patient can be calculated by multiplying the probability that a patient is a certain age, by the probability that they are categorized in a certain diagnostic group, by the probability of having a certain P/F ratio given their age and diagnosis group, by the probability of being on mechanical ventilation given their P/F ratio.

The distribution of each variable in the Bayesian network can be estimated from relevant data by applying any number of statistical or machine learning approaches, with coefficients corresponding to parent variables. For example, the distribution of age might be estimated using ordinary least squares regression with an intercept term only, resulting in a predicted normal distribution for Pr(age); while the P/F ratio distribution might be estimated using a log-linear model and incorporating coefficients for age and diagnosis groups, resulting in a predicted lognormal distribution for Pr(P/F ratio | age, diagnosis group).

**Table A** - Final priors used for each donor characteristic

| **Outcome/Descendent Variable** | **Final Priors** |
| --- | --- |
| Hospital/Date | gamma(alpha = 2.75, beta = 1) |
| Sex | beta(alpha = 1.5, beta = 1) |
| Cause of Death | Coefficient matrix: normal(mean = 0, standard deviation (sd) = 0.55) |
| Height | Coefficient matrix for mean: normal(mean = 0, sd = 10)  Standard Error: normal(mean = 0, sd = 5) restricted to > 0 |
| DCD status | Coefficient matrix: normal(mean = 0, sd = 1) |
| Age | Mean: normal(mean = 40, sd = 5)  Standard Error: normal(mean = 5, sd = 1) restricted to > 0  Skewness: normal(mean = 0, sd = 3) |
| Race/Ethnicity | Coefficient matrix: normal(mean = 0, sd = 0.655) |
| Blood Type | Coefficient matrix: normal(mean = 0, sd = 2) |
| >20 pack year Smoking History | Coefficient matrix: normal(mean = 0, sd = 2) |
| Lungs Available  (Double, Left, or Right) | -- |

**Table B** - Final priors used for each candidate risk factor

| **Outcome/Descendent Variable** | **Predictor(s)/Parent Variable(s)** |
| --- | --- |
| Center/Date/Diagnosis Group | Group A: gamma(alpha = 2.3, beta = 1)  Group B: gamma(alpha = 0.8 beta = 2)  Group C: gamma(alpha = 1.0, beta = 2)  Group D: gamma(alpha = 6.25, beta = 1) |
| Sex | beta(alpha = 1, beta = 1) |
| Race/Ethnicity | Coefficient matrix: normal(mean = 0, standard deviation (sd) = 0.7) |
| Height | Coefficient matrix for mean: normal(mean = 0, sd = 10)  Standard Error: normal(mean = 0, sd = 5) restricted to > 0 |
| Weight | Coefficient matrix for mean: normal(mean = 3, sd = 1)  Standard Error: normal(mean = 0, sd = 3) restricted to > 0 |
| Weight (Group C) | Coefficient matrix for mean: normal(mean = 0, sd = 3)  Standard Error: normal(mean = 0, sd = 10) restricted to > 0  Skewness: normal(mean = 0, sd = 2) |
| Age | Mean: normal(mean = 65, sd = 10)  Standard Error: normal(mean = 10, sd = 2) restricted to > 0  Skewness: normal(mean = -8, sd = 2) |
| Airway Function Index | Mean: normal(mean = 0, sd = 0.25)  Standard Error: normal(mean = 0, sd = .10) restricted to > 0  Skewness: normal(mean = 0, sd = 0.20) |
| Oxygen Function Index | Mean: normal(mean = 0, sd = 0.25)  Added Coefficient matrix for mean: normal(mean = 0, sd = 0.10)  Standard Error: normal(mean = 0, sd = .10) restricted to > 0  Skewness: normal(mean = 0, sd = 0.20) |
| Respiratory Support Cluster* | Coefficient matrix: normal(mean = 0, sd = 2) |
| FEV_1_ | Coefficient matrix for mean: normal(mean = 0, sd = 3)  Standard Error: normal(mean = 0, sd = 10) restricted to > 0 |
| FVC | Coefficient matrix for mean: normal(mean = 0, sd = 3)  Standard Error: normal(mean = 0, sd = 10) restricted to > 0 |
| pCO_2_ | Coefficient matrix for mean: normal(mean = 0, sd = 0.05)  Standard Error: normal(mean = 0, sd = 0.05) restricted to > 0 |
| P/F Ratio | Coefficient matrix for mean: normal(mean = 0, sd = 3)  Standard Error: normal(mean = 0, sd = 5) restricted to > 0 |
| pO_2_ | Coefficient matrix for mean: normal(mean = 0, sd = 0.05)  Standard Error: normal(mean = 0, sd = 0.05) restricted to > 0 |
| Mean Pulmonary Artery Pressure (PAP) | Coefficient matrix for mean: normal(mean = 0, sd = 3)  Standard Error: normal(mean = 0, sd = 5) restricted to > 0 |
| Oxygen Frequency | Coefficient matrix: normal(mean = 0, sd = 5) |
| Ventilator | Coefficient matrix: normal(mean = 0, sd = 5) |
| Supplemental Oxygen Requirement | Coefficient matrix: normal(mean = 0, sd = 2)  Thresholds: normal(mean = 0, sd = 5) |
| Six Minute Walk Distance | Coefficient matrix for mean: normal(mean = 0, sd = 1000)  Standard Error: normal(mean = 100, sd = 10) restricted to > 0  Zero Inflated Odds: beta(alpha = 1, beta = 2) |
| Bilirubin | Coefficient matrix for mean: normal(mean = 0, sd = 2)  Standard Error: normal(mean = 0, sd = 1) restricted to > 0 |
| Creatinine | Coefficient matrix for mean: normal(mean = 0, sd = 2)  Standard Error: normal(mean = 0, sd = 1) restricted to > 0 |
| Systolic PAP | Coefficient matrix for mean: normal(mean = 0, sd = 20)  Standard Error: normal(mean = 0, sd = 20) restricted to > 0 |
| Cardiac Index | Coefficient matrix for mean: normal(mean = 0, sd = 2)  Standard Error: normal(mean = 0, sd = 2) restricted to > 0 |
| Central Venous Pressure | Coefficient matrix for alpha: normal(mean = 0, sd = 5)  Standard Error: normal(mean = 0, sd = 5) restricted to > 0 |
| Functional Status | Coefficient matrix: normal(mean = 0, sd = 2) |
| pCO_2_ Threshold | Coefficient matrix: normal(mean = 0, sd = 2) |
| Blood Type | Coefficient matrix: normal(mean = 0, sd = 2) |
| Surgical Type | Coefficient matrix: normal(mean = 0, sd = 3.5) |
| Diabetes | Coefficient matrix: normal(mean = 0, sd = 3) |
| Sarcoidosis | Group A: Coefficient matrix: normal(mean = 0, sd = 0.5)  Group D: Coefficient matrix: normal(mean = 0, sd = 0.5) |
| Eisenmenger Syndrome | Coefficient matrix: normal(mean = 0, sd = 4) |
| Lymphangioleiomyomatosis (LAM) | Coefficient matrix: normal(mean = 0, sd = 0.7) |
| Other Specific Diagnoses from Lung Allocation Score models   - Bronchiectasis - Pulmonary Fibrosis (other) - Constrictive Bronchiolitis - Bronchiolitis Obliterans | Group A: Coefficient matrix: normal(mean = 0, sd = 0.5)  Group D: Coefficient matrix: normal(mean = 0, sd = 0.7) |

**References**

1. Koller D, Friedman D. *Probabilistic Graphical Models: Principles and Techniques.* Cambridge, Massachusetts. The MIT Press; 2009.
2. Pearl J. *Causality: Models, Reasoning, and Inference*. New York. Cambridge University Press; 2000.
3. Pearl J, Mackenzie, D. *The Book of Why: The New Science of Cause and Effect.* New York. Basic Books; 2018.
