# Supplementary figures and images for: Creating synthetic populations in transplantation: A Bayesian approach enabling simulation without registry re-sampling
Source: PLoS One. 2024 Mar 21;19(3):e0296839. doi: 10.1371/journal.pone.0296839 (PMC10956776; doi:10.1371/journal.pone.0296839)

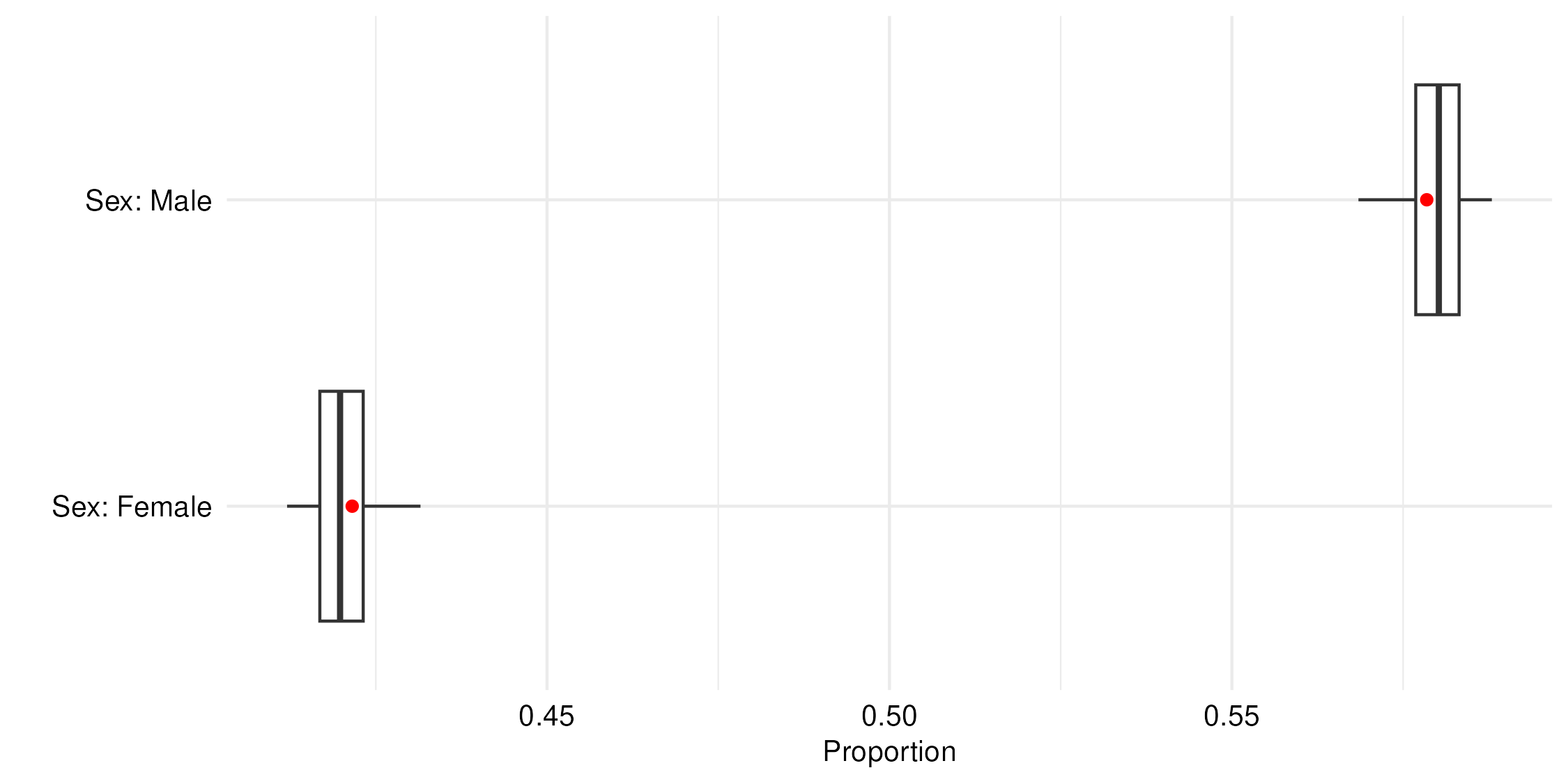

Supplement: S1 Fig — Each box horizontally spans the interquartile range (IQR), while the vertical line inside each box represents the median. The extremes of each whisker mark the observation that is farthest to the left or right of the median while still falling within 1.5 times the IQR. Black dots represent outlying synthetic population counts, defined as greater then +/-1.5 times the IQR from the third and first quartiles respectively. (TIF) [file pone.0296839.s002.tif]

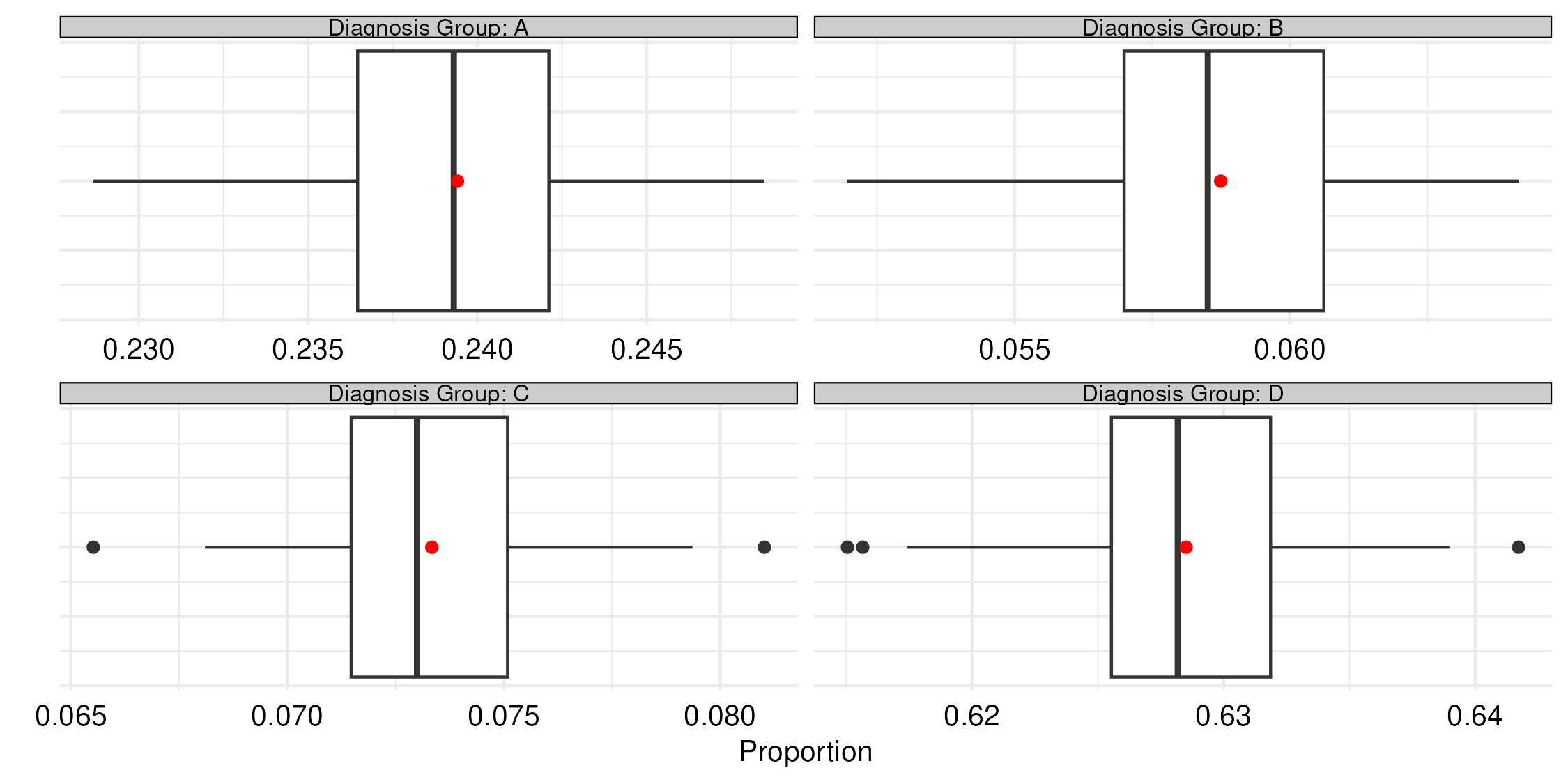

Supplement: S2 Fig — (TIF) [file pone.0296839.s003.tif]

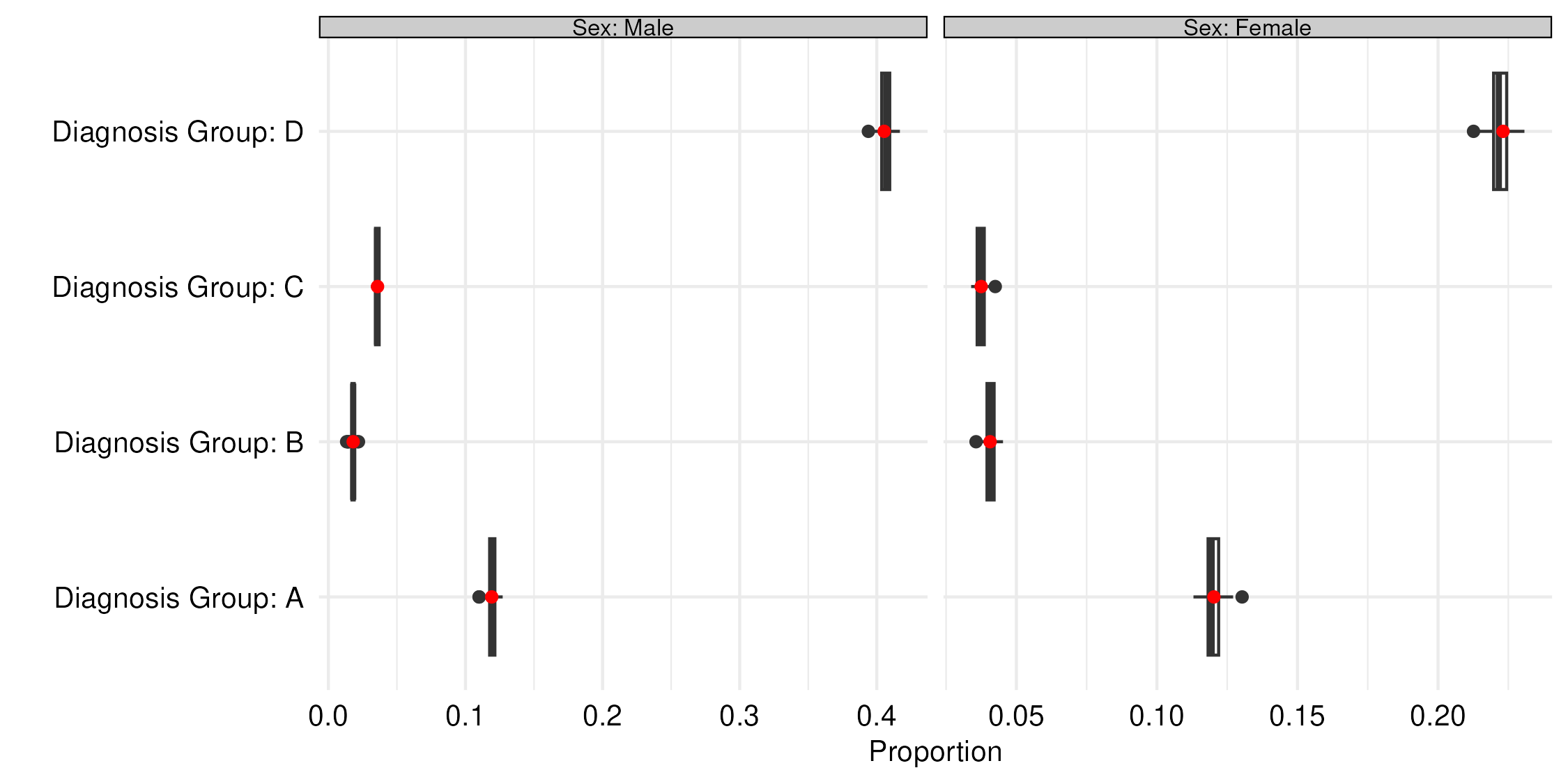

Supplement: S3 Fig — (TIF) [file pone.0296839.s004.tif]

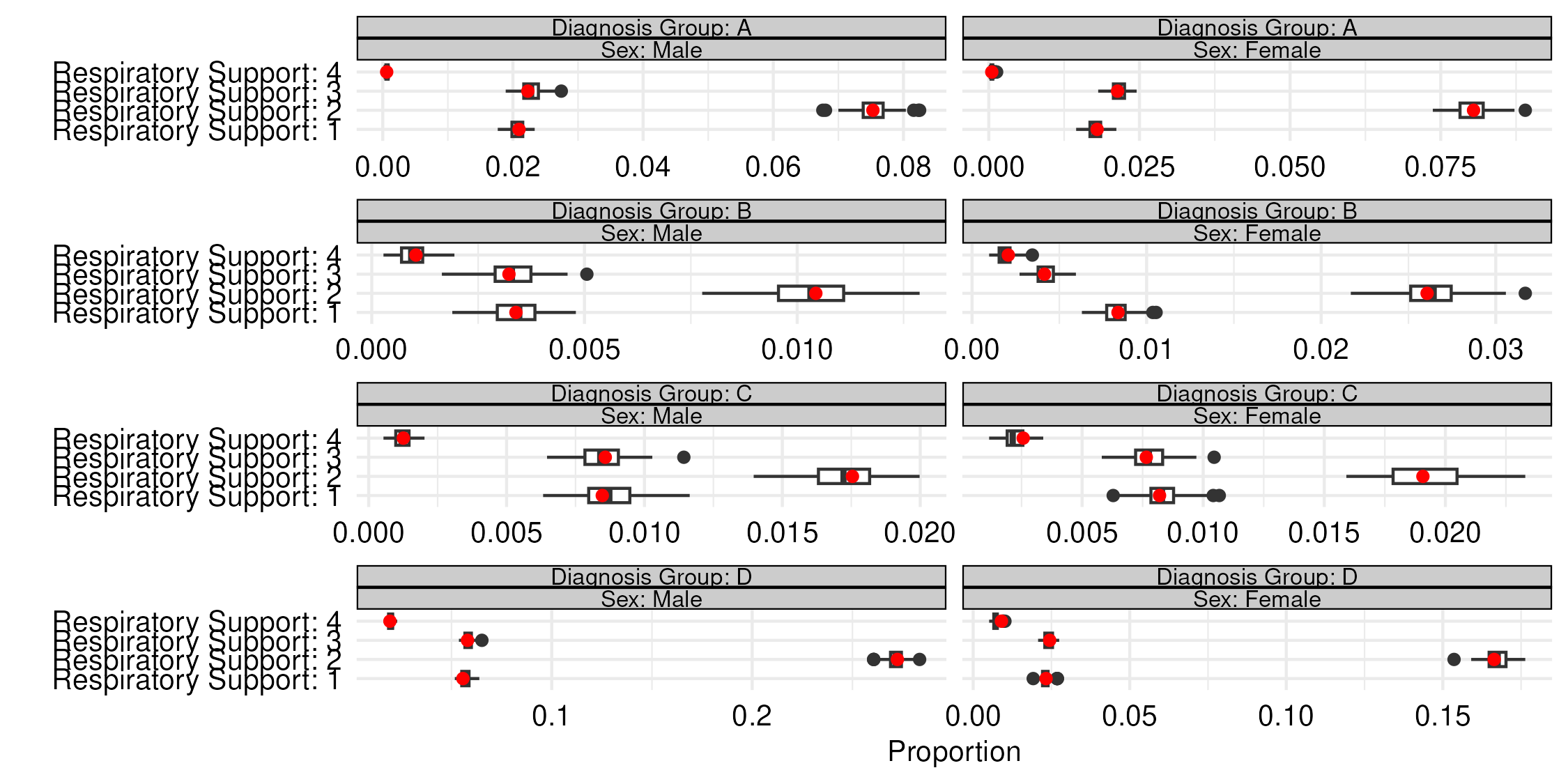

Supplement: S4 Fig — (TIF) [file pone.0296839.s005.tif]

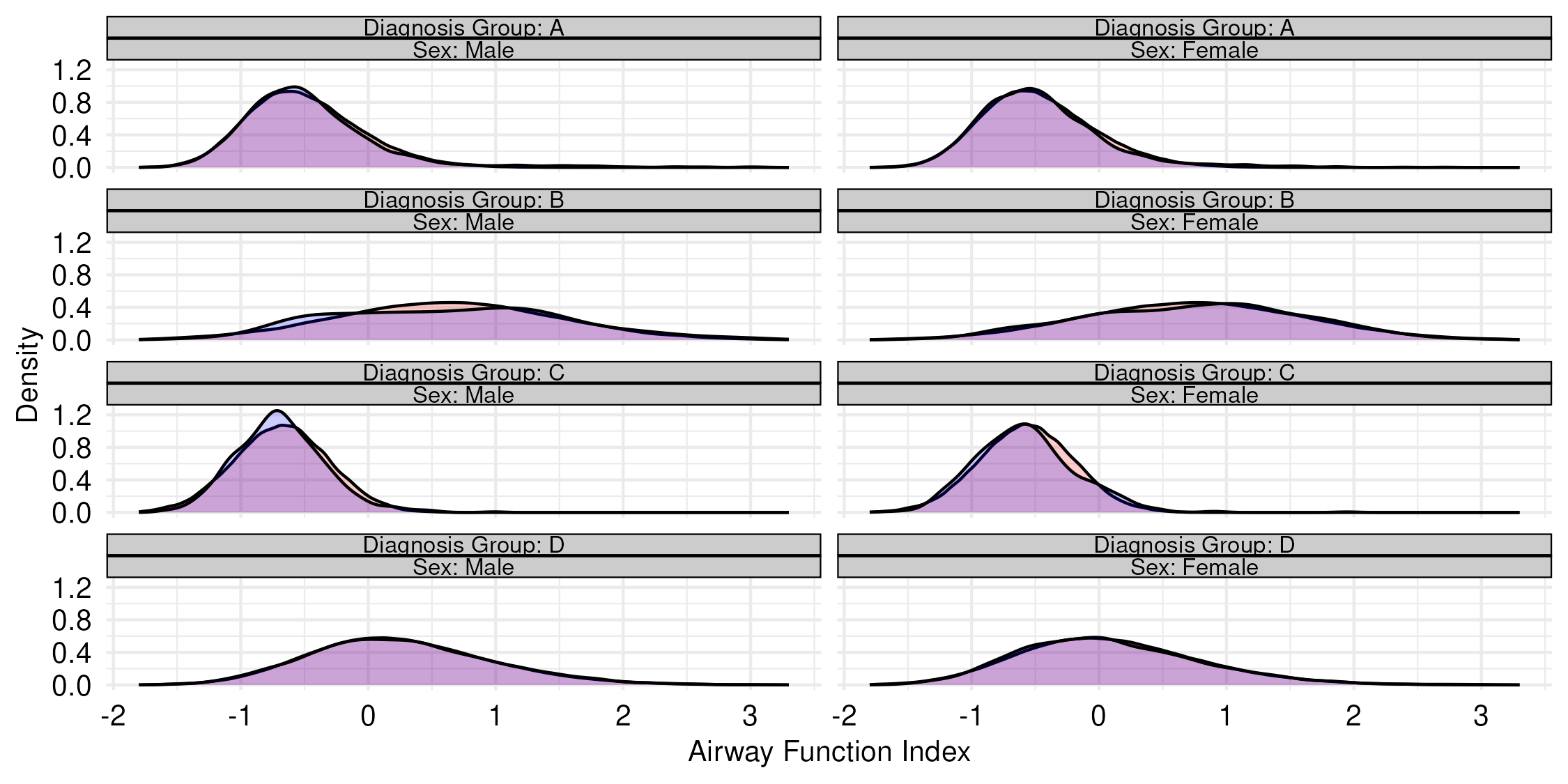

Supplement: S5 Fig — In each, the original SRTR data is represented with a blue shaded density plot, while the combined output from 100 synthetic populations is represented by a red shaded density plot. (TIF) [file pone.0296839.s006.tif]

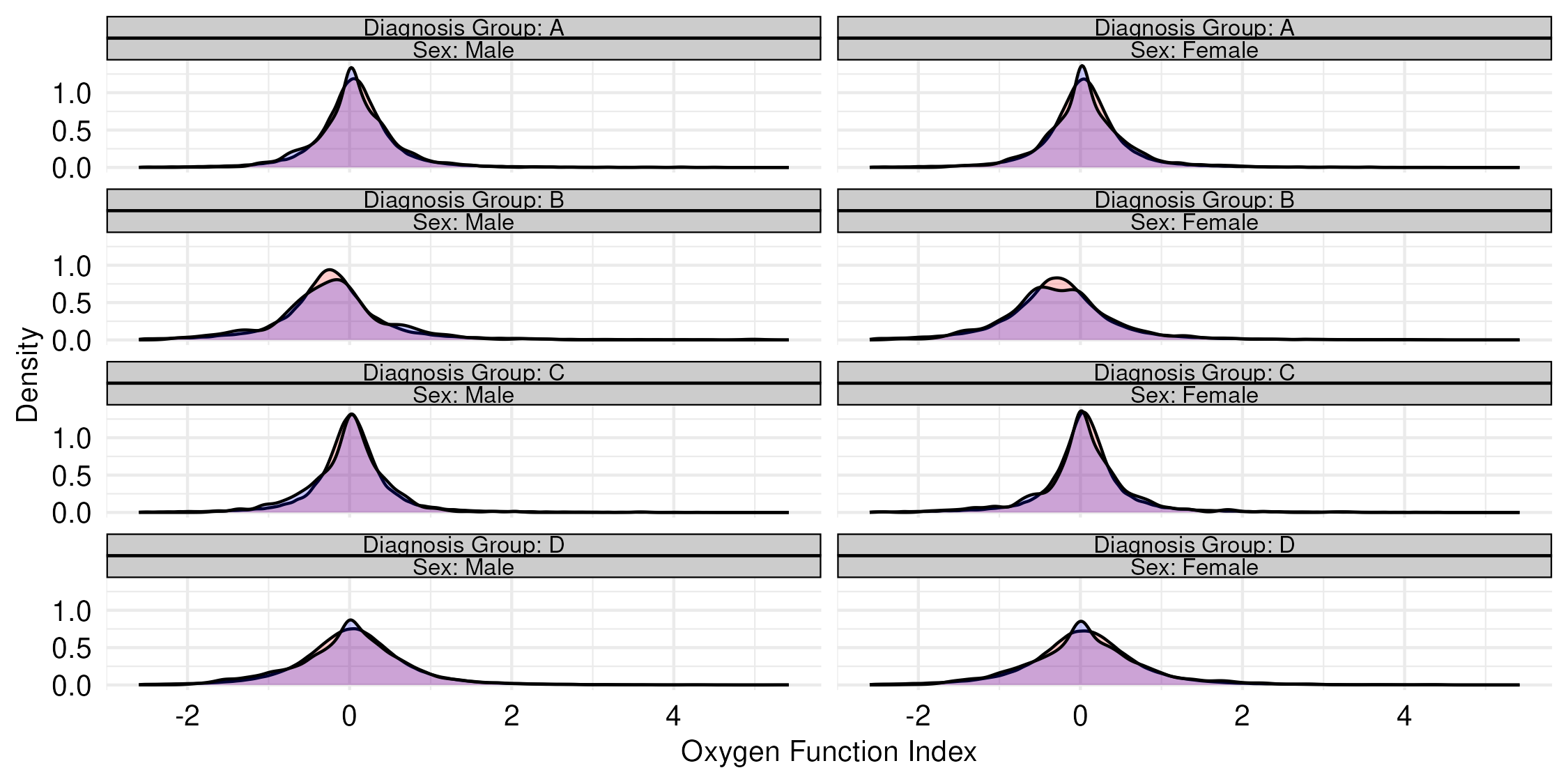

Supplement: S6 Fig — In each, the original SRTR data is represented with a blue shaded density plot, while the combined output from 100 synthetic populations is represented by a red shaded density plot. (TIF) [file pone.0296839.s007.tif]

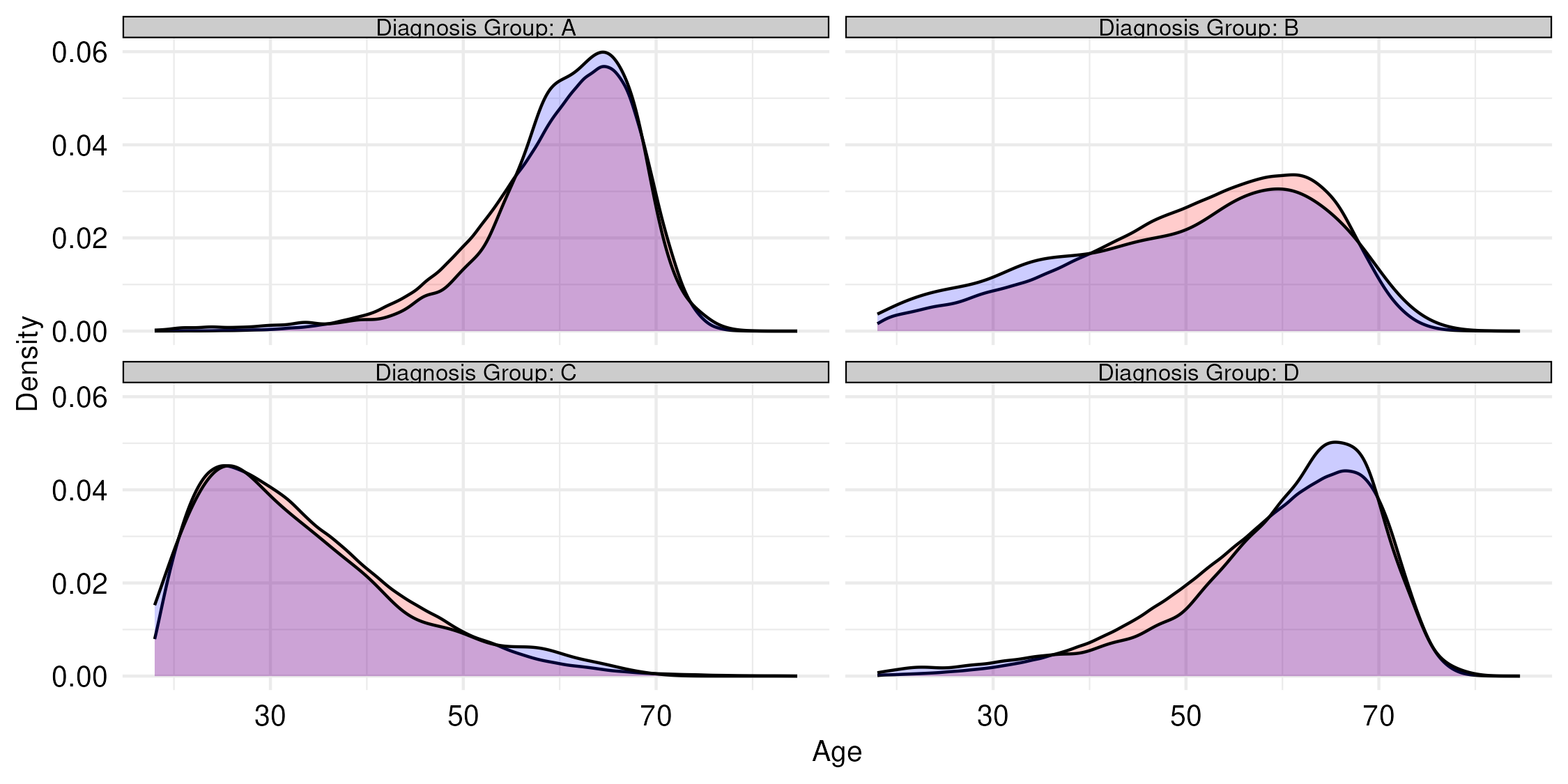

Supplement: S7 Fig — In each, the original SRTR data is represented with a blue shaded density plot, while the combined output from 100 synthetic populations is represented by a red shaded density plot. (TIF) [file pone.0296839.s008.tif]

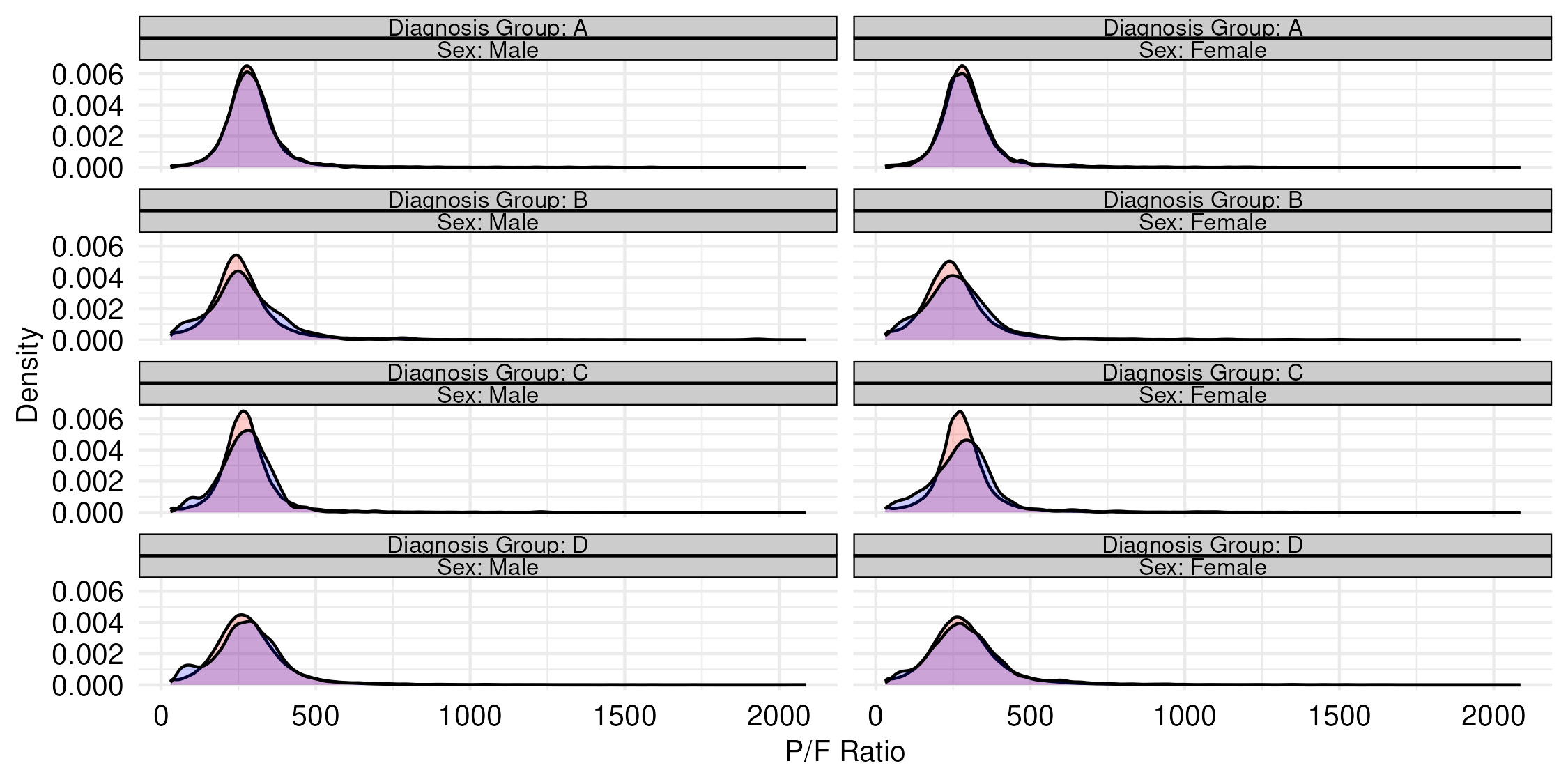

Supplement: S8 Fig — In each, the original SRTR data is represented with a blue shaded density plot, while the combined output from 100 synthetic populations is represented by a red shaded density plot. (TIF) [file pone.0296839.s009.tif]

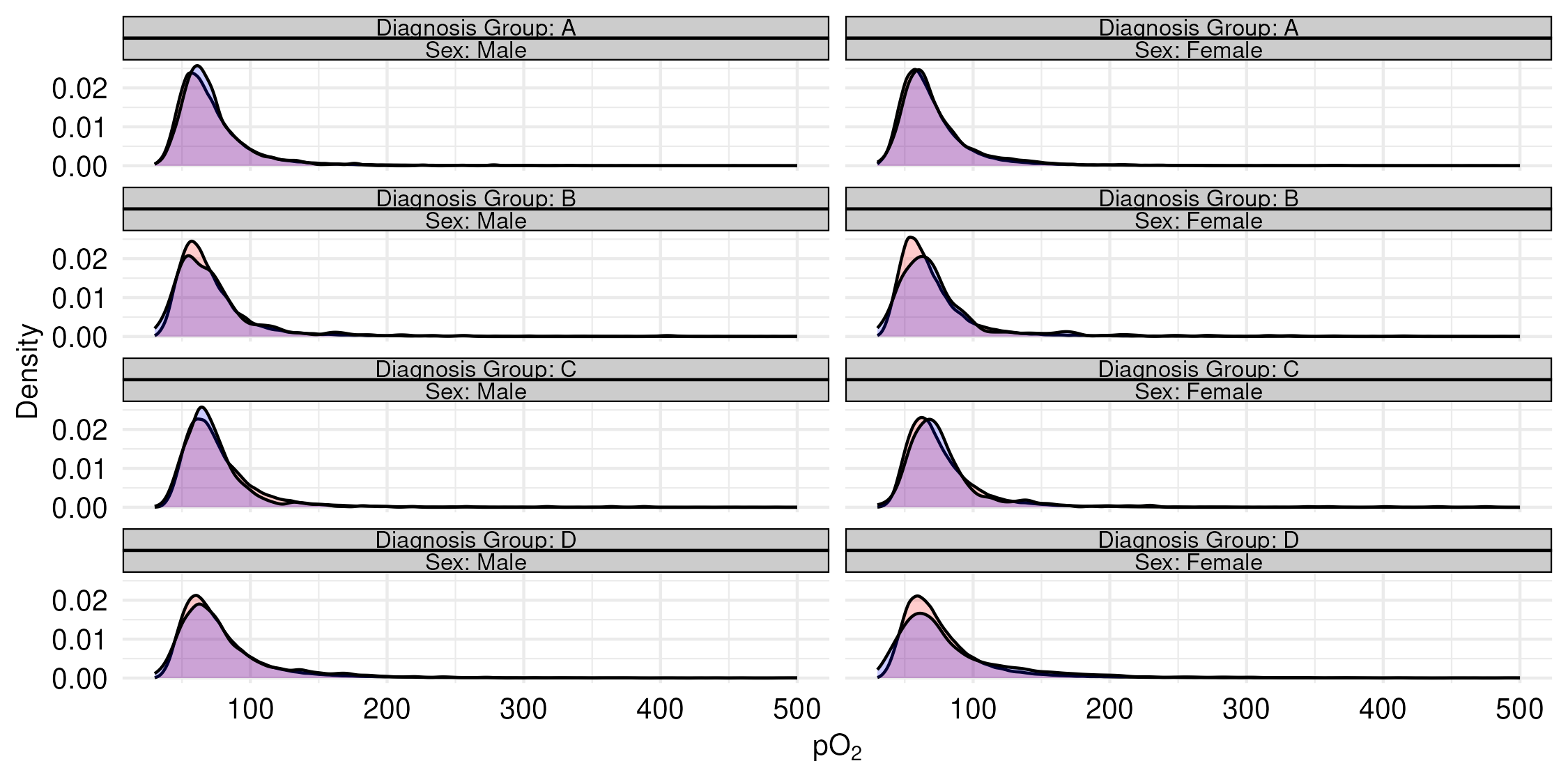

Supplement: S9 Fig — In each, the original SRTR data is represented with a blue shaded density plot, while the combined output from 100 synthetic populations is represented by a red shaded density plot. (TIF) [file pone.0296839.s010.tif]

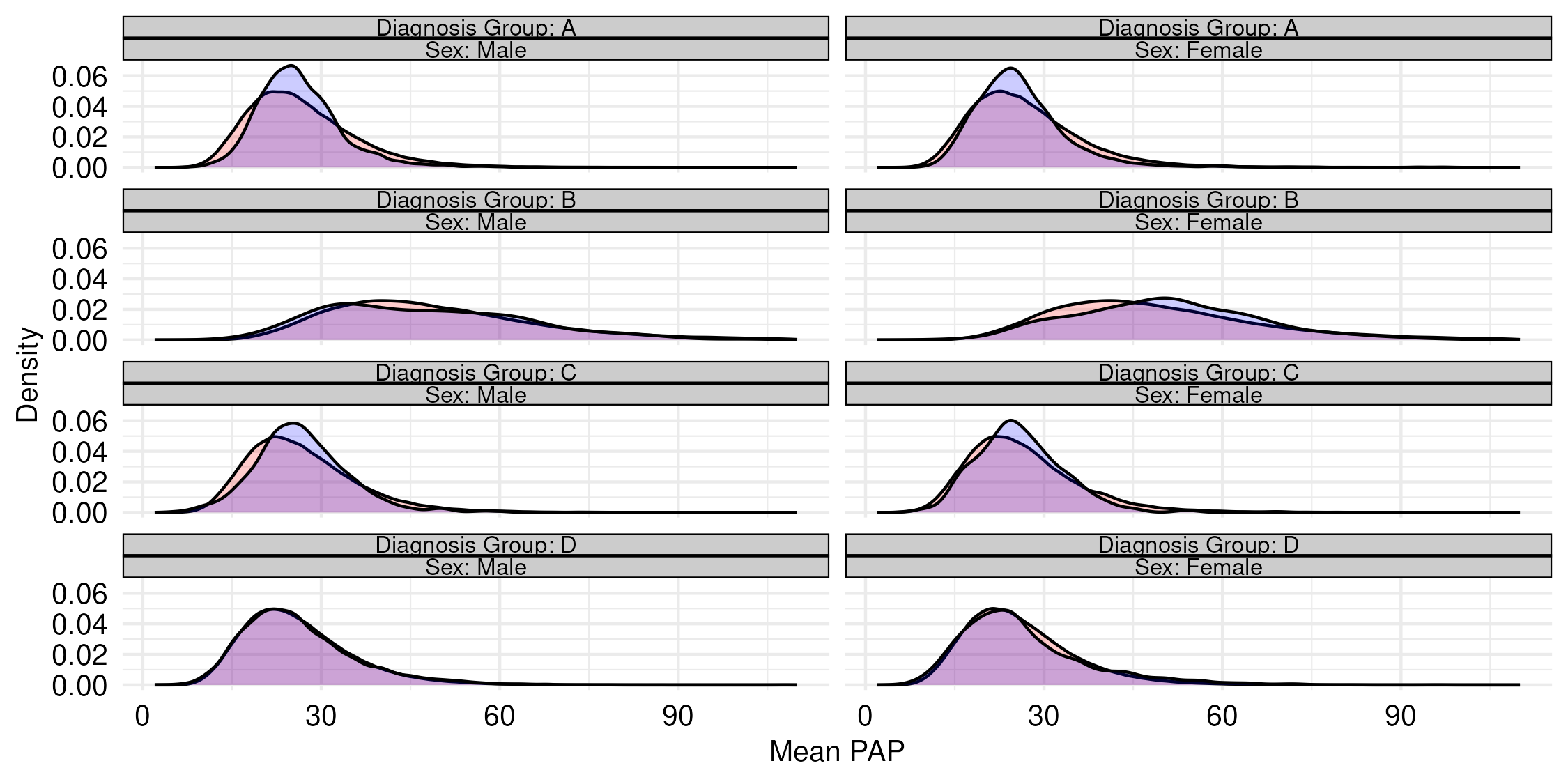

Supplement: S10 Fig — In each, the original SRTR data is represented with a blue shaded density plot, while the combined output from 100 synthetic populations is represented by a red shaded density plot. (TIF) [file pone.0296839.s011.tif]

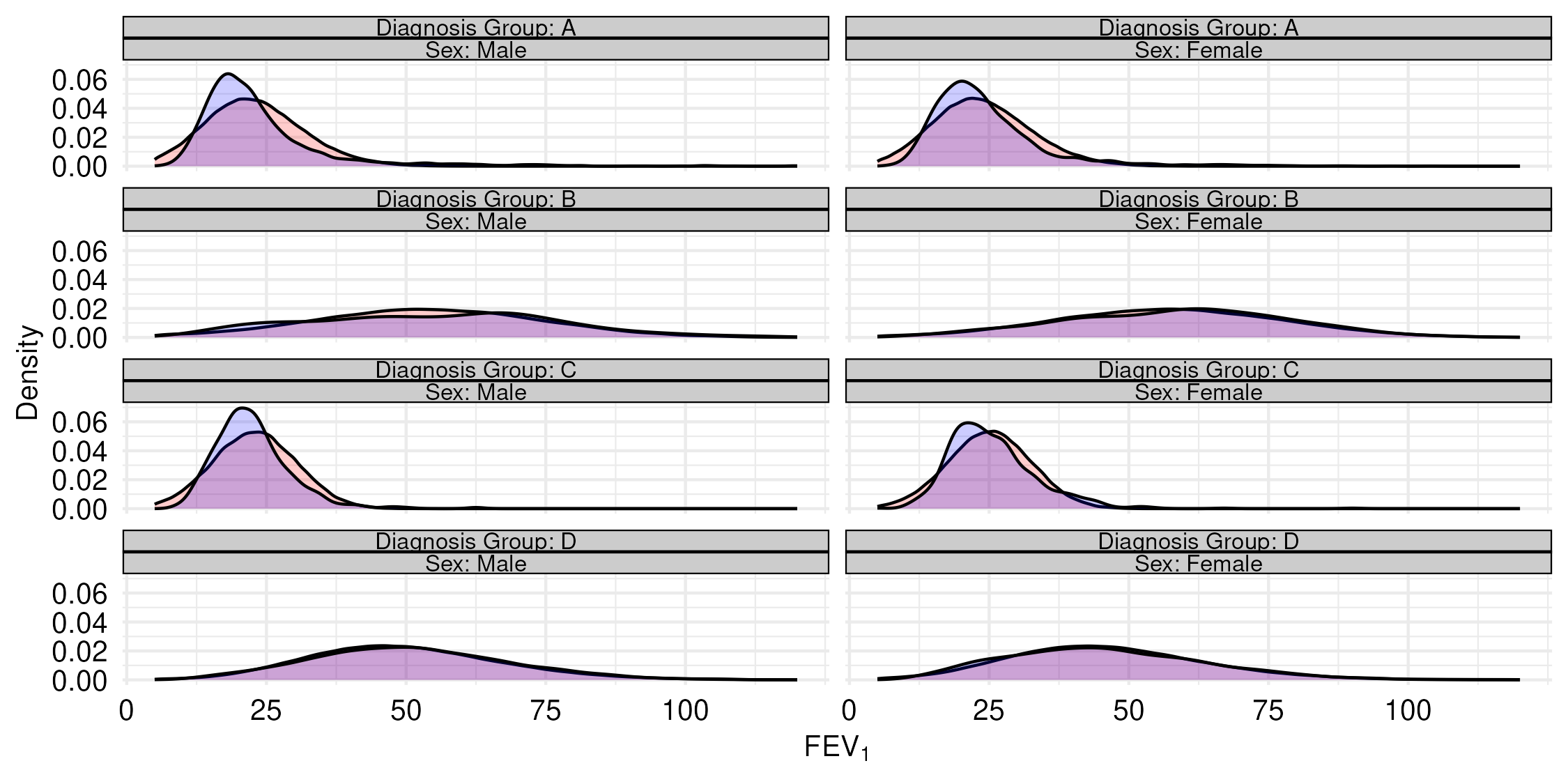

Supplement: S11 Fig — In each, the original SRTR data is represented with a blue shaded density plot, while the combined output from 100 synthetic populations is represented by a red shaded density plot. (TIF) [file pone.0296839.s012.tif]

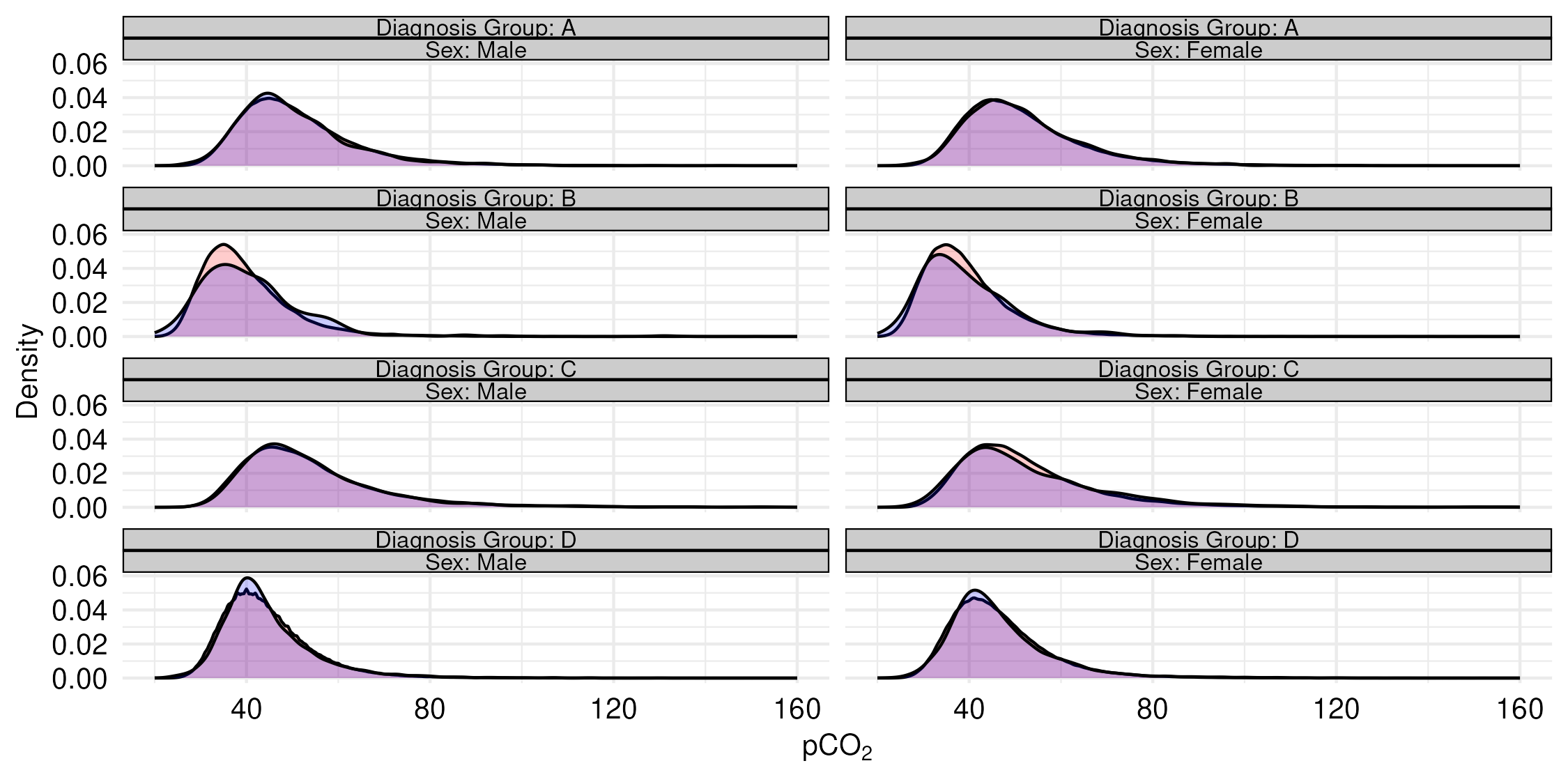

Supplement: S12 Fig — In each, the original SRTR data is represented with a blue shaded density plot, while the combined output from 100 synthetic populations is represented by a red shaded density plot. (TIF) [file pone.0296839.s013.tif]

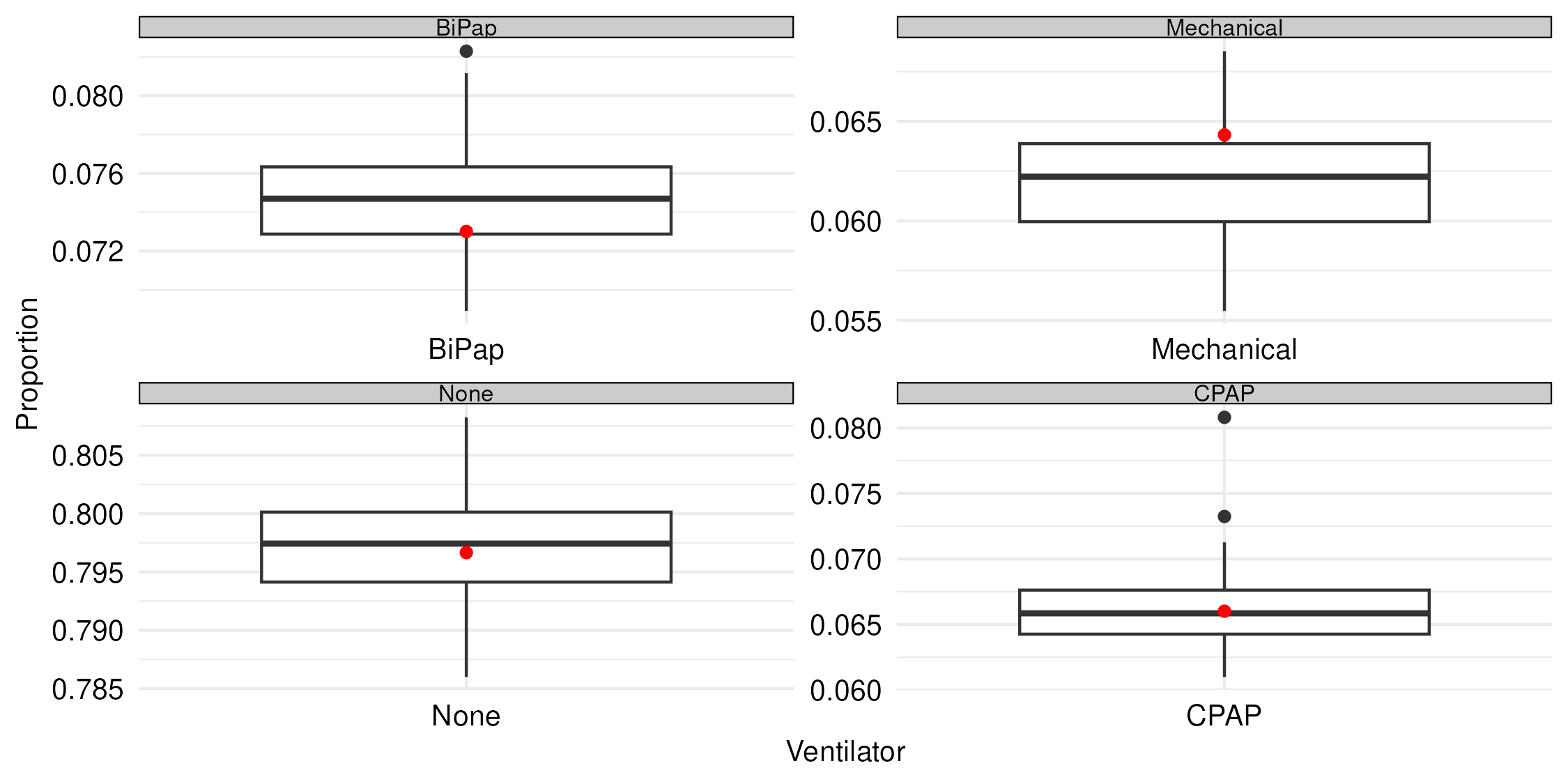

Supplement: S13 Fig — (TIF) [file pone.0296839.s014.tif]

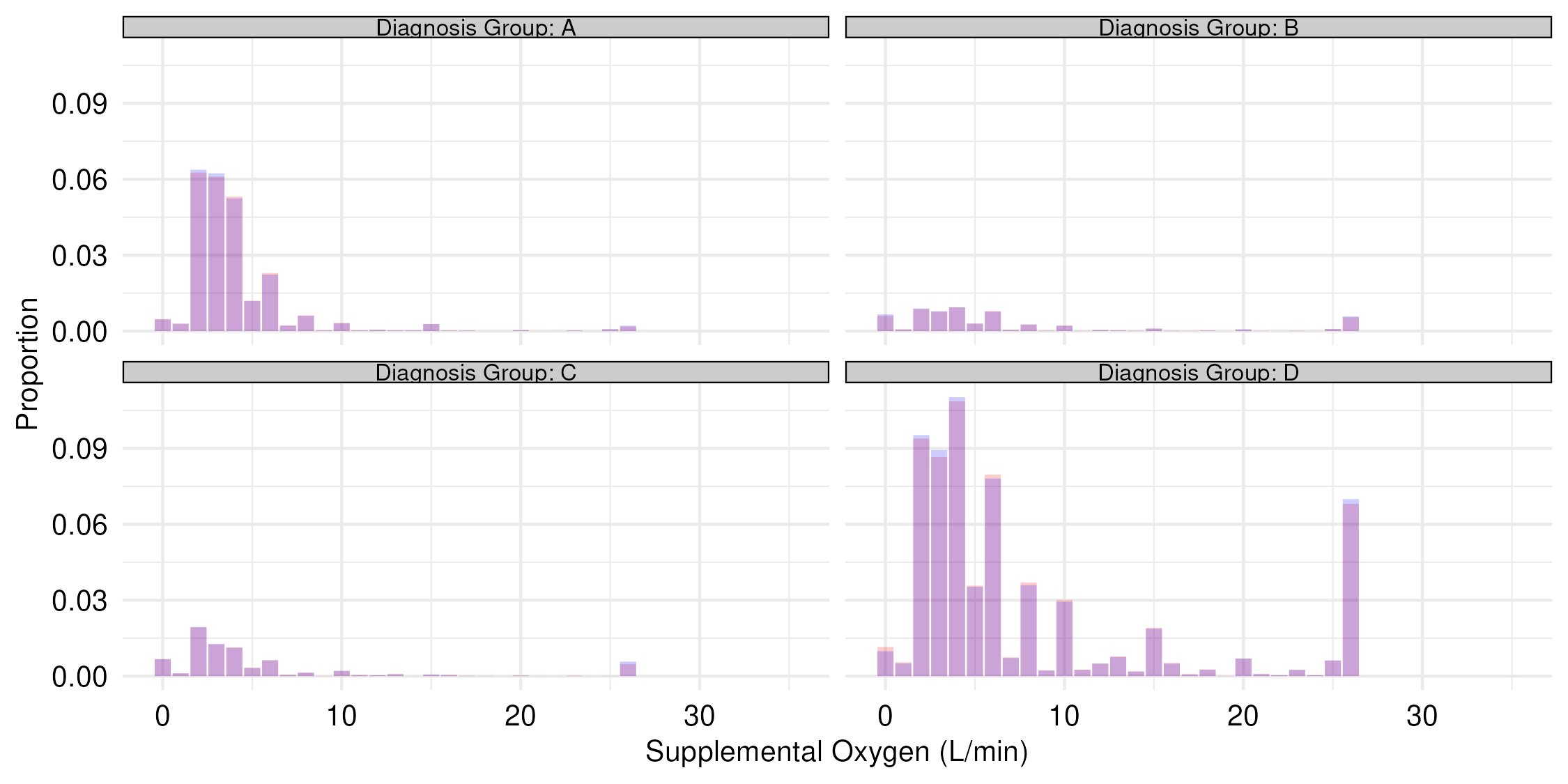

Supplement: S14 Fig — In each, the original SRTR data is represented with a blue shaded bars, while the combined output from 100 synthetic populations is represented by a red shaded bars. (TIF) [file pone.0296839.s015.tif]

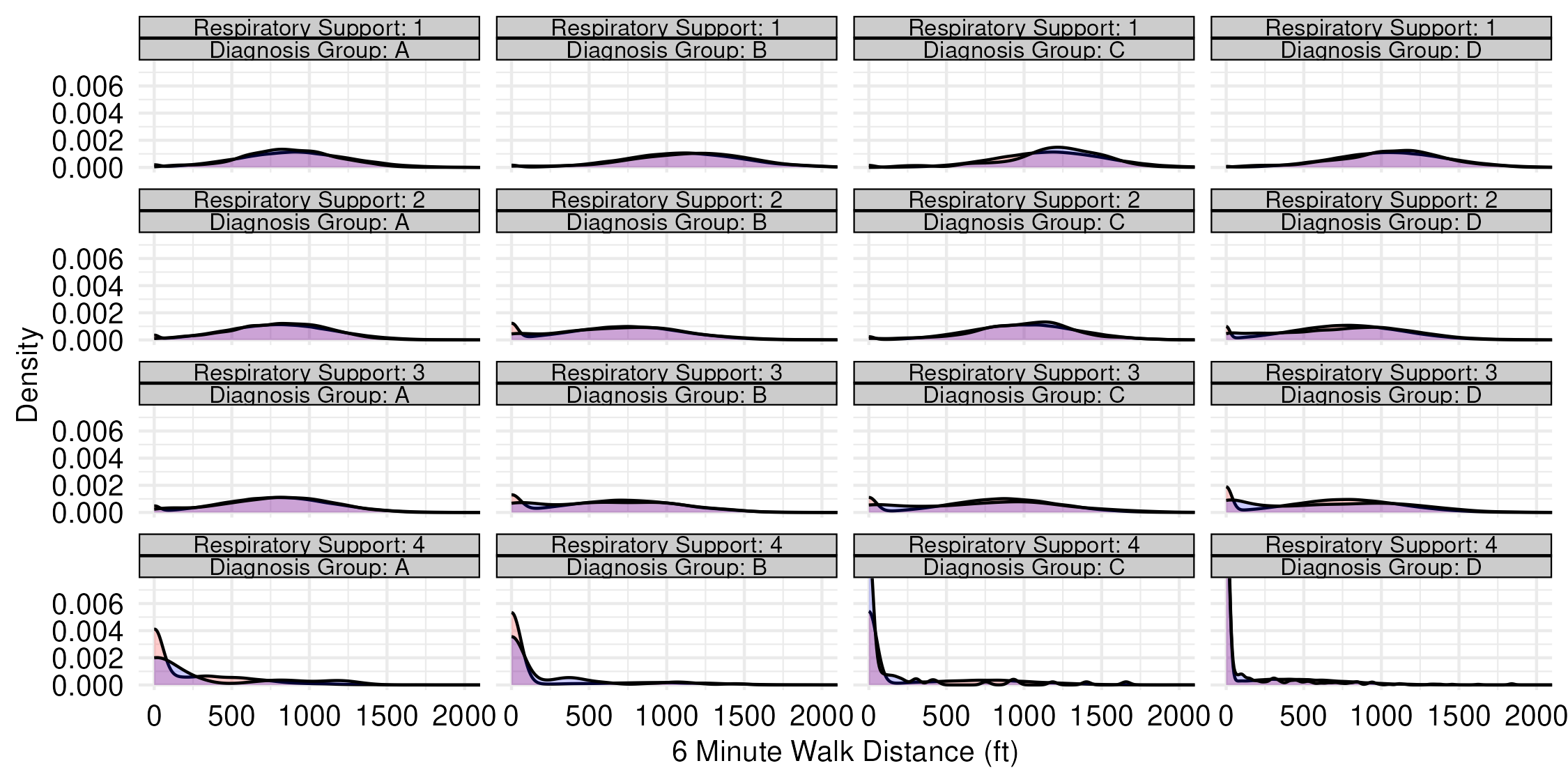

Supplement: S15 Fig — In each, the original SRTR data is represented with a blue shaded density plot, while the combined output from 100 synthetic populations is represented by a red shaded density plot. (TIF) [file pone.0296839.s016.tif]

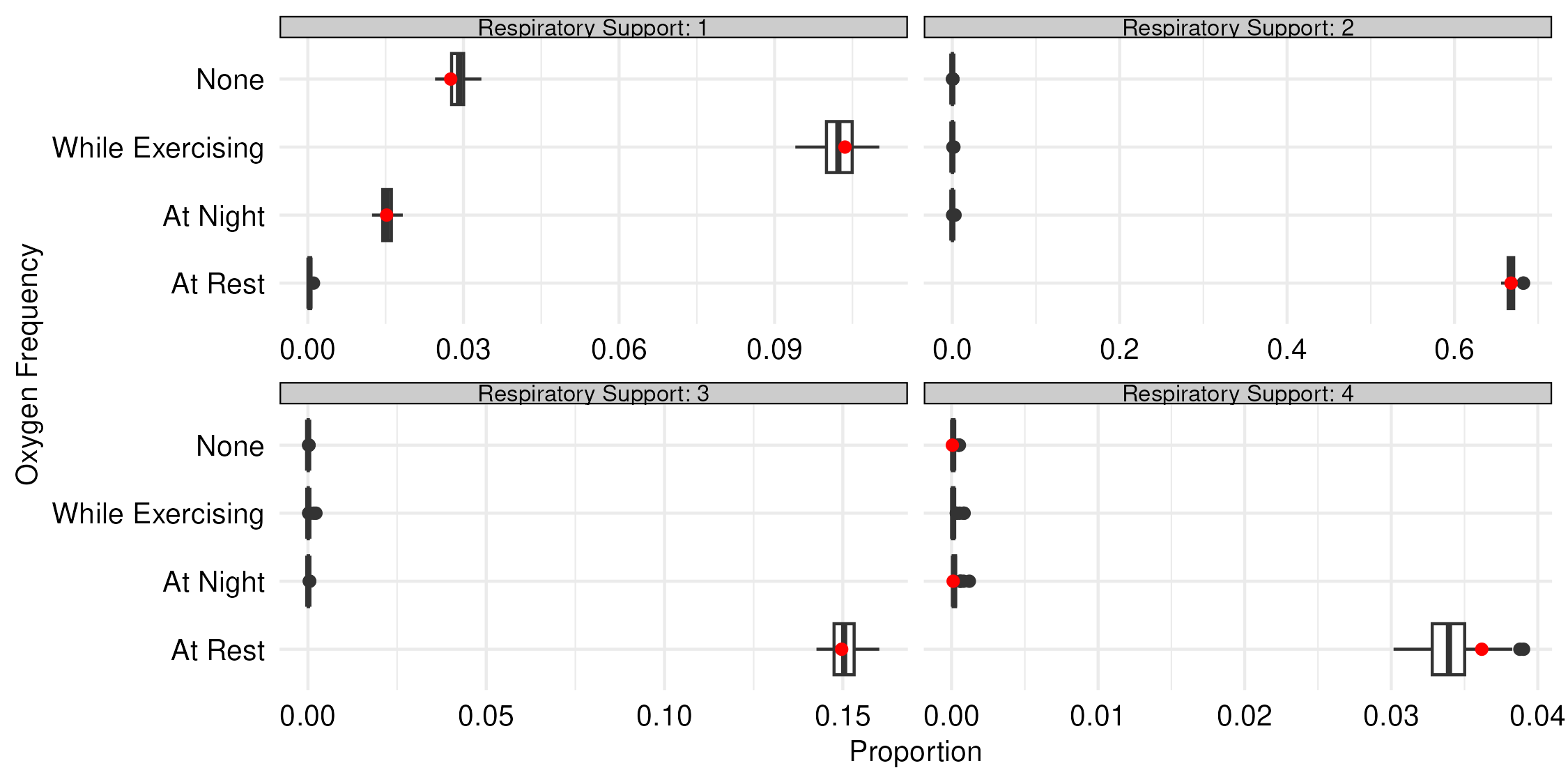

Supplement: S16 Fig — (TIF) [file pone.0296839.s017.tif]

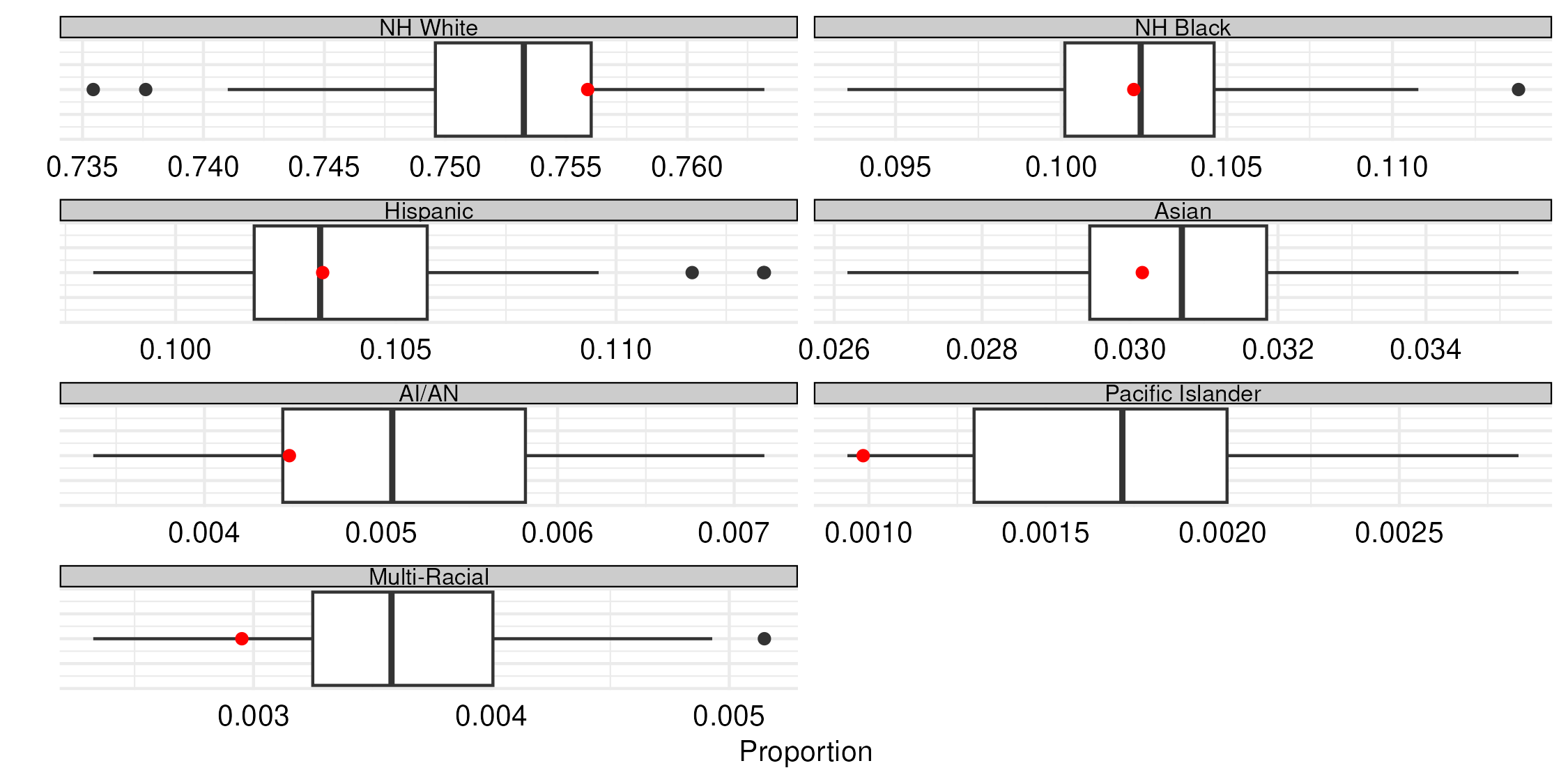

Supplement: S17 Fig — (TIF) [file pone.0296839.s018.tif]

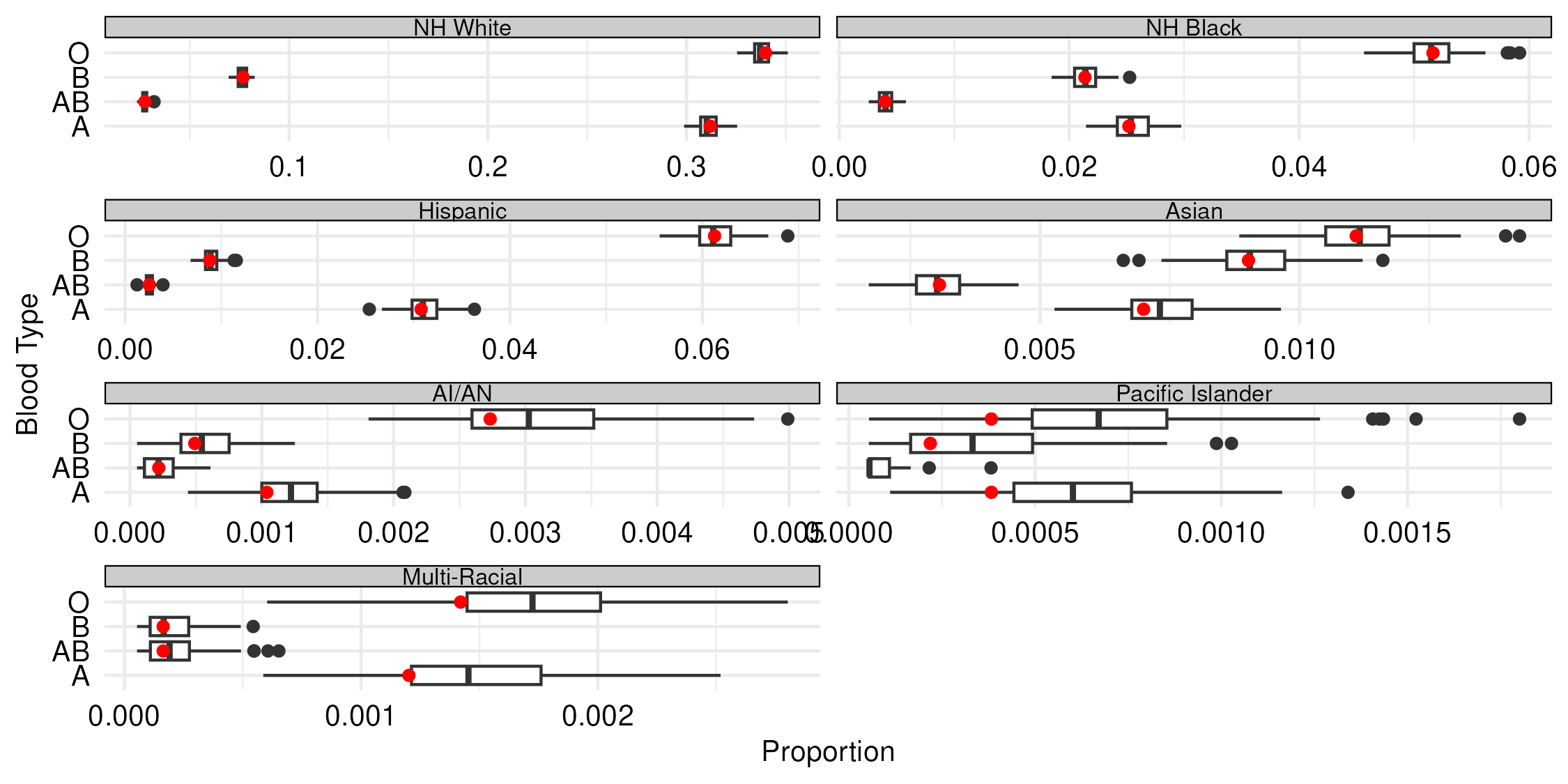

Supplement: S18 Fig — (TIF) [file pone.0296839.s019.tif]

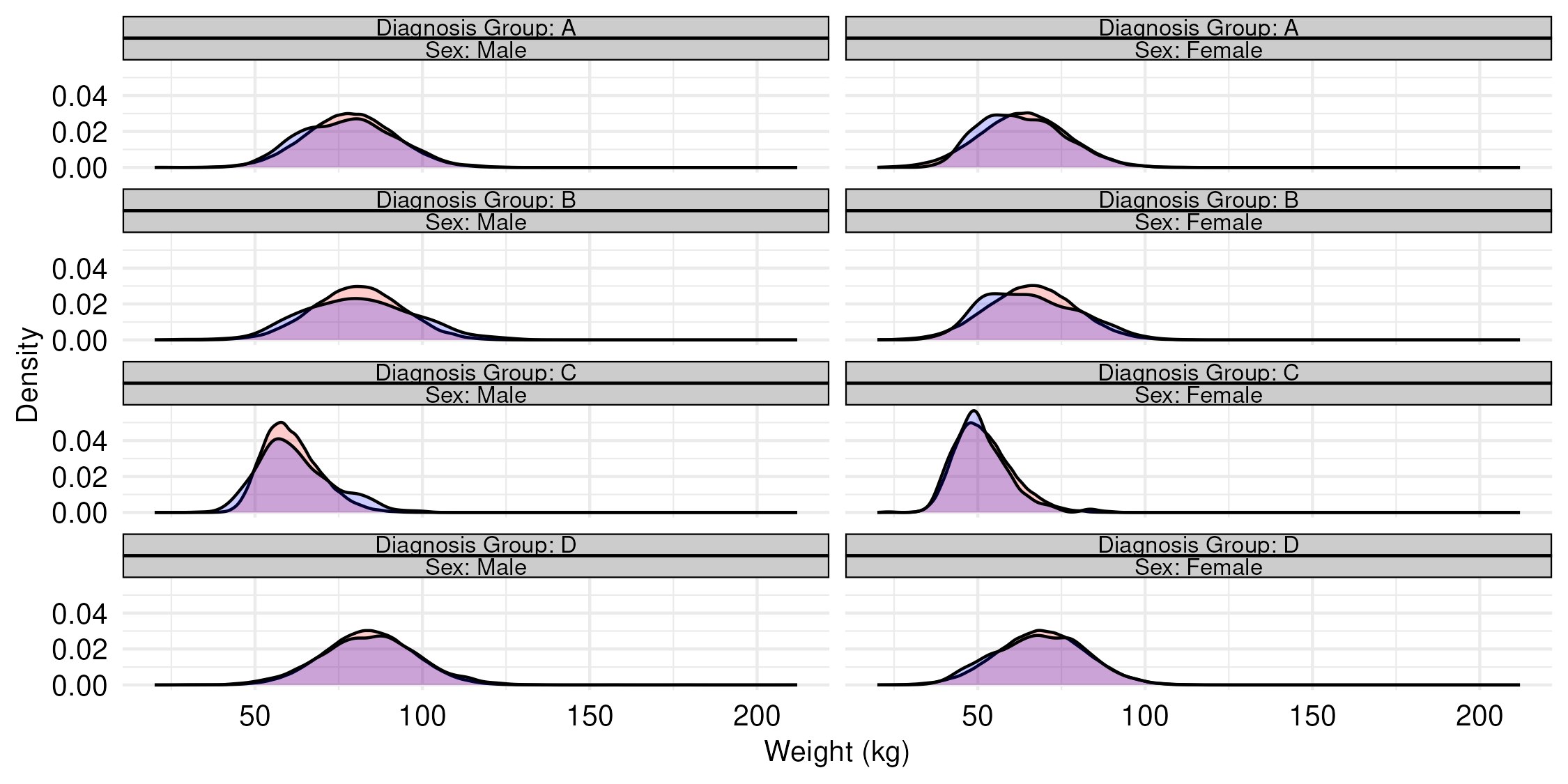

Supplement: S19 Fig — In each, the original SRTR data is represented with a blue shaded density plot, while the combined output from 100 synthetic populations is represented by a red shaded density plot. (TIF) [file pone.0296839.s020.tif]

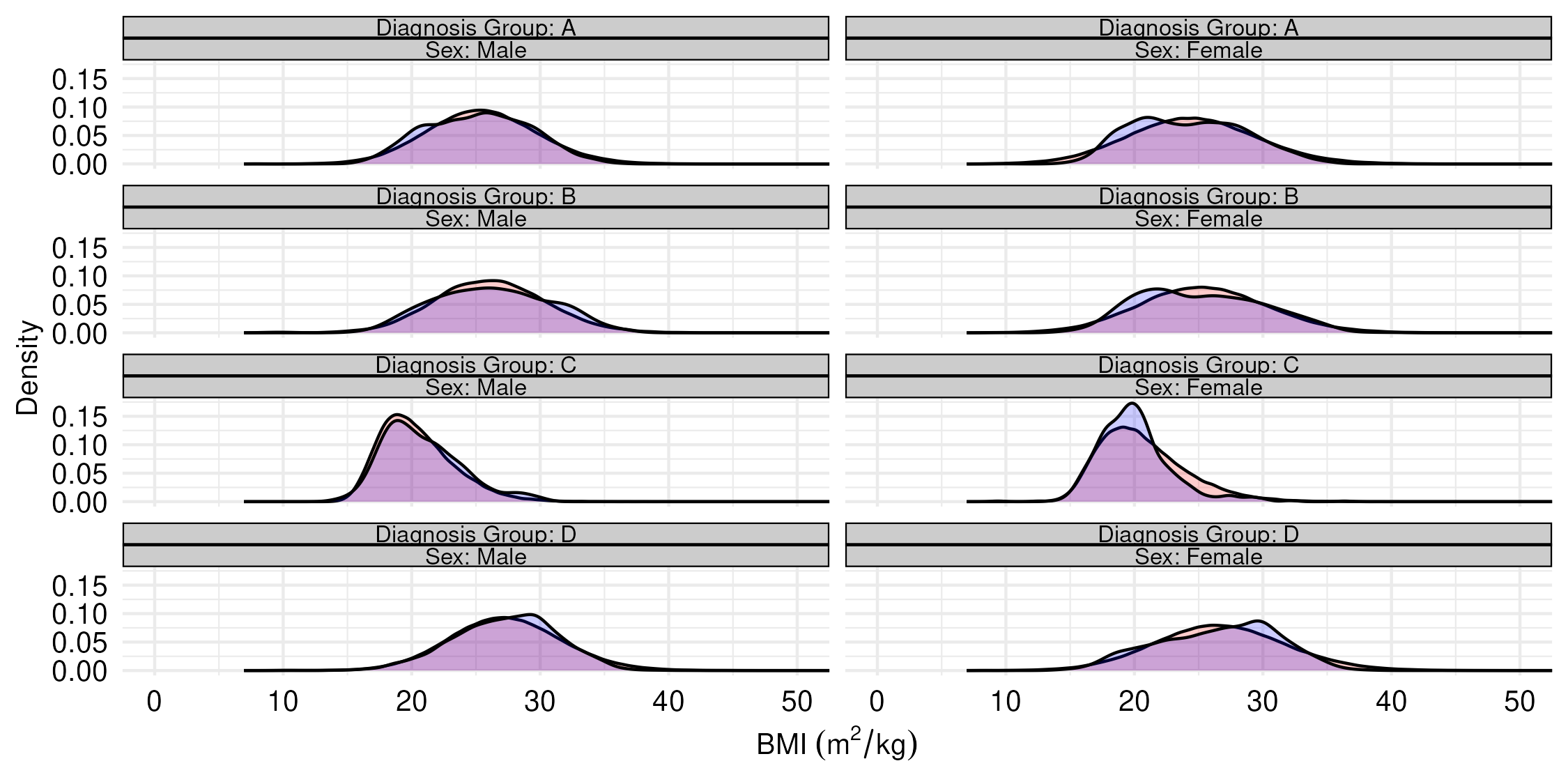

Supplement: S20 Fig — In each, the original SRTR data is represented with a blue shaded density plot, while the combined output from 100 synthetic populations is represented by a red shaded density plot. (TIF) [file pone.0296839.s021.tif]

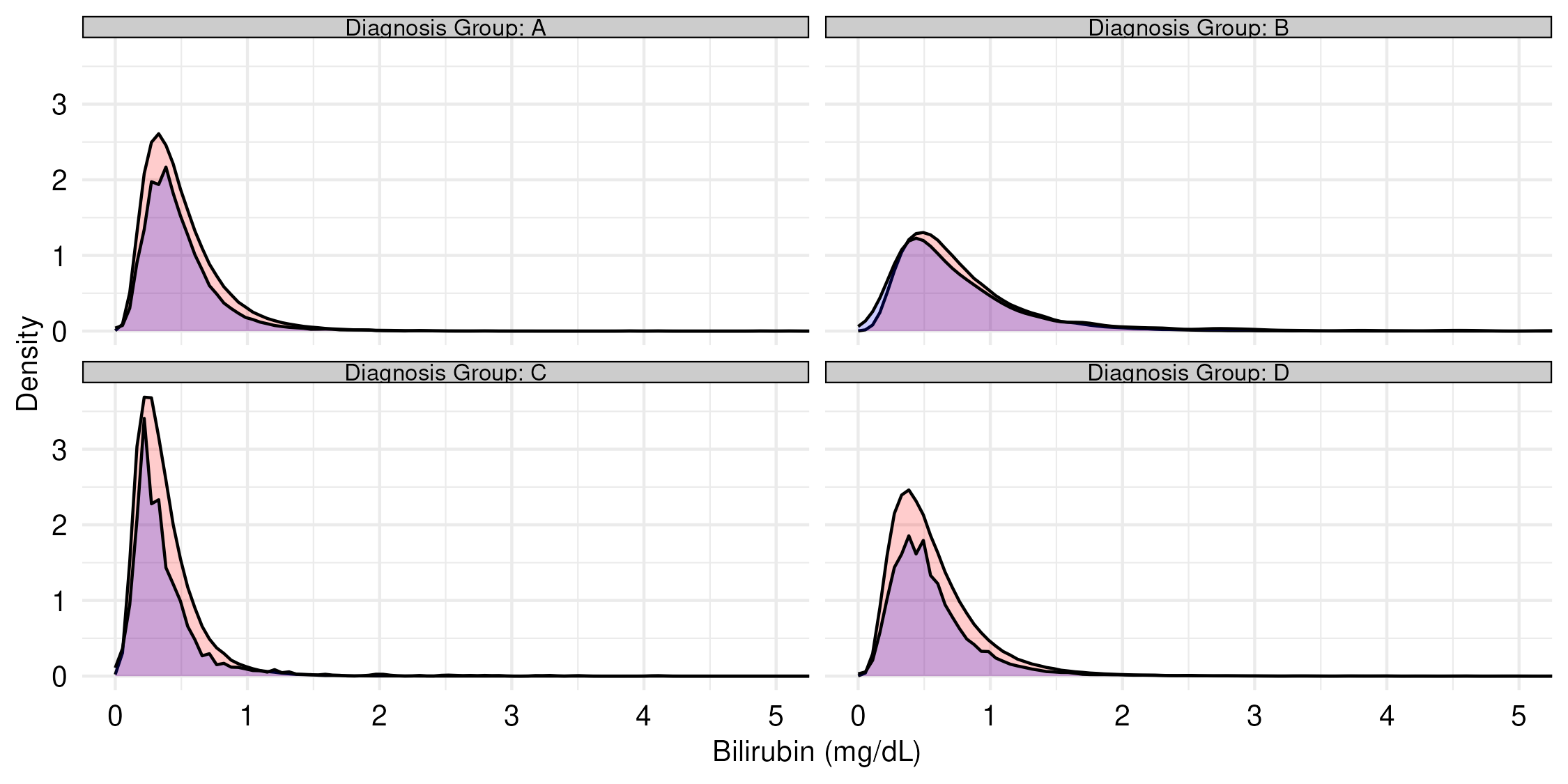

Supplement: S21 Fig — In each, the original SRTR data is represented with a blue shaded density plot, while the combined output from 100 synthetic populations is represented by a red shaded density plot. (TIF) [file pone.0296839.s022.tif]

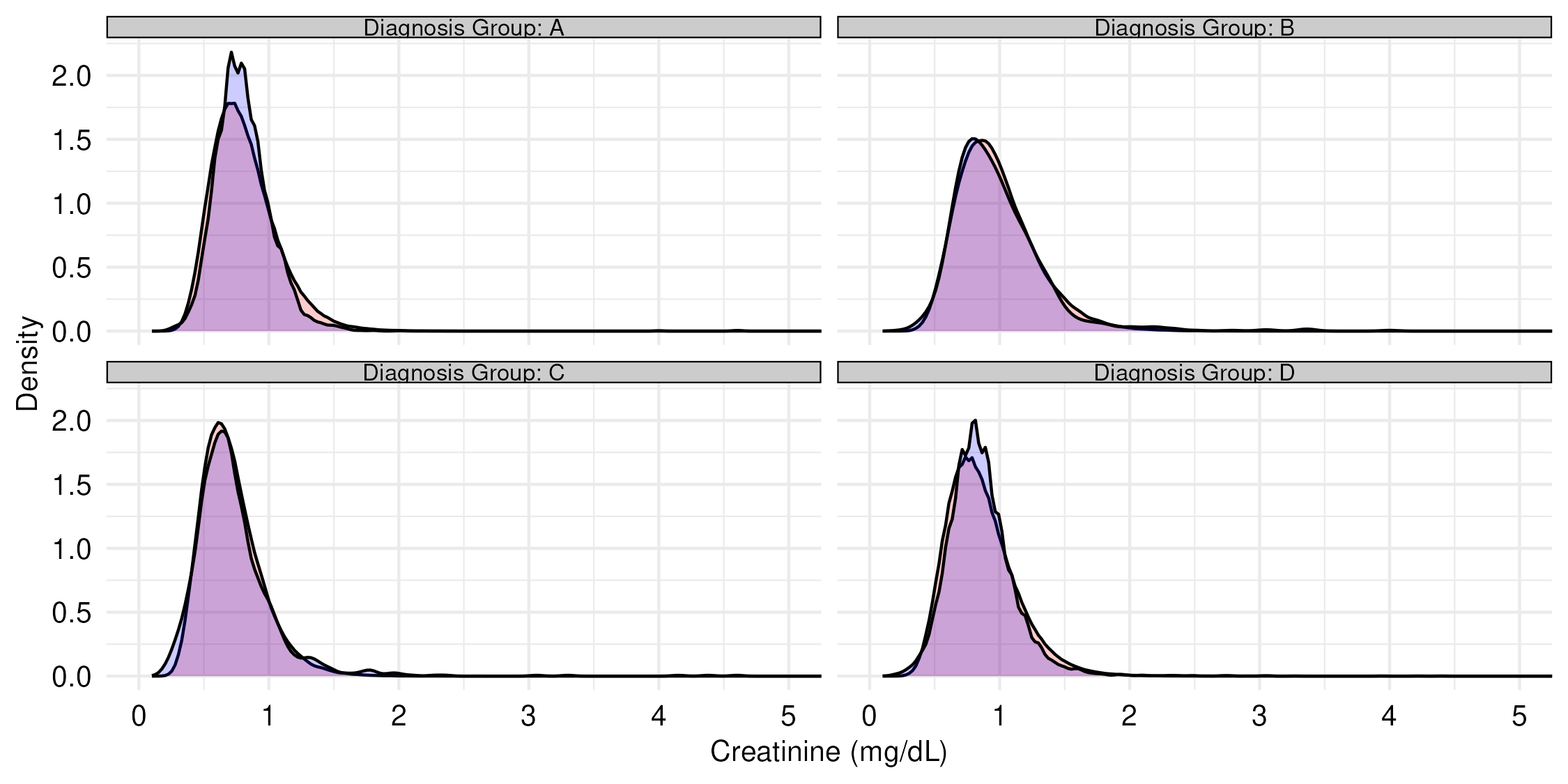

Supplement: S22 Fig — In each, the original SRTR data is represented with a blue shaded density plot, while the combined output from 100 synthetic populations is represented by a red shaded density plot. (TIF) [file pone.0296839.s023.tif]

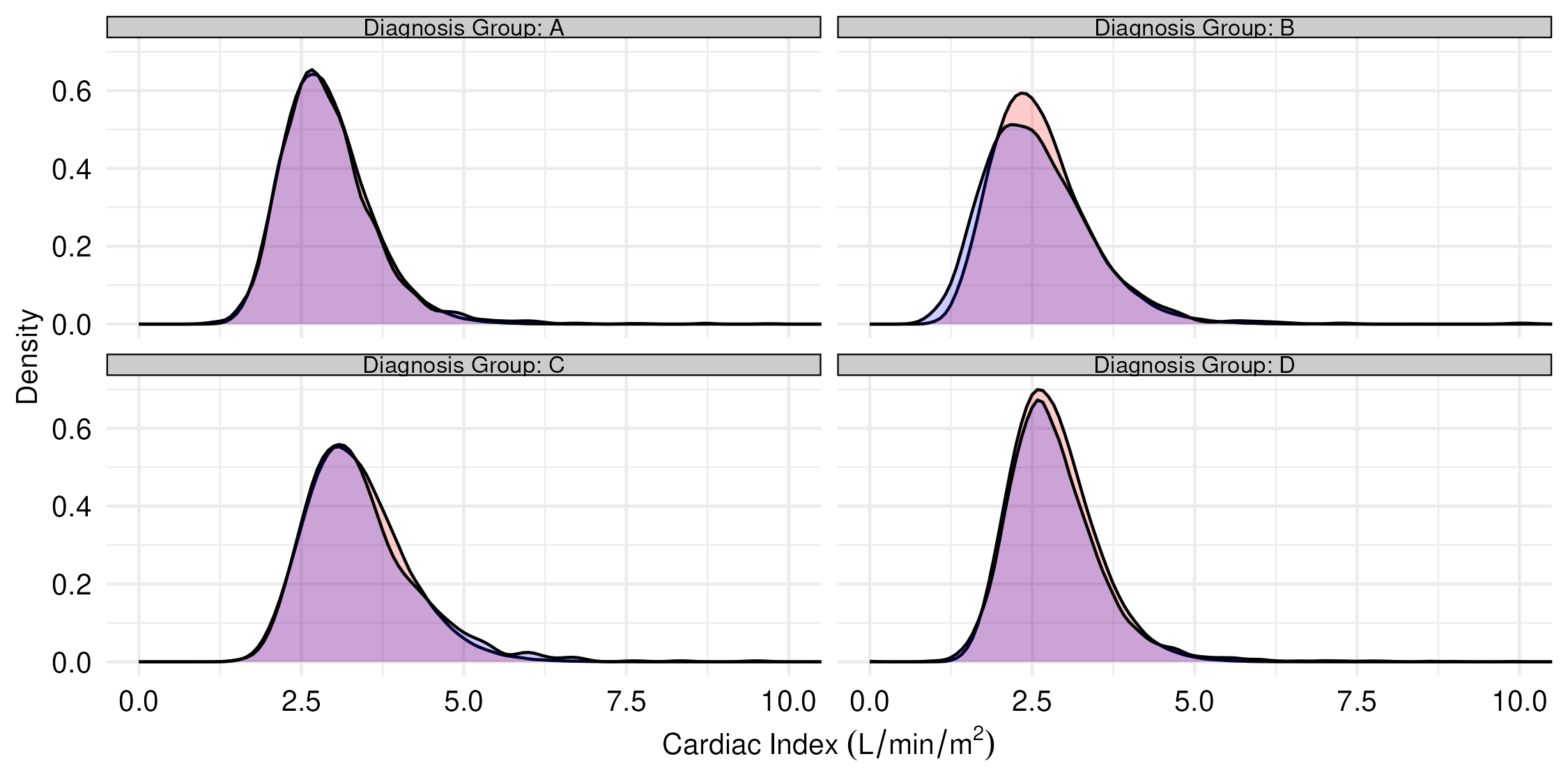

Supplement: S23 Fig — In each, the original SRTR data is represented with a blue shaded density plot, while the combined output from 100 synthetic populations is represented by a red shaded density plot. (TIF) [file pone.0296839.s024.tif]

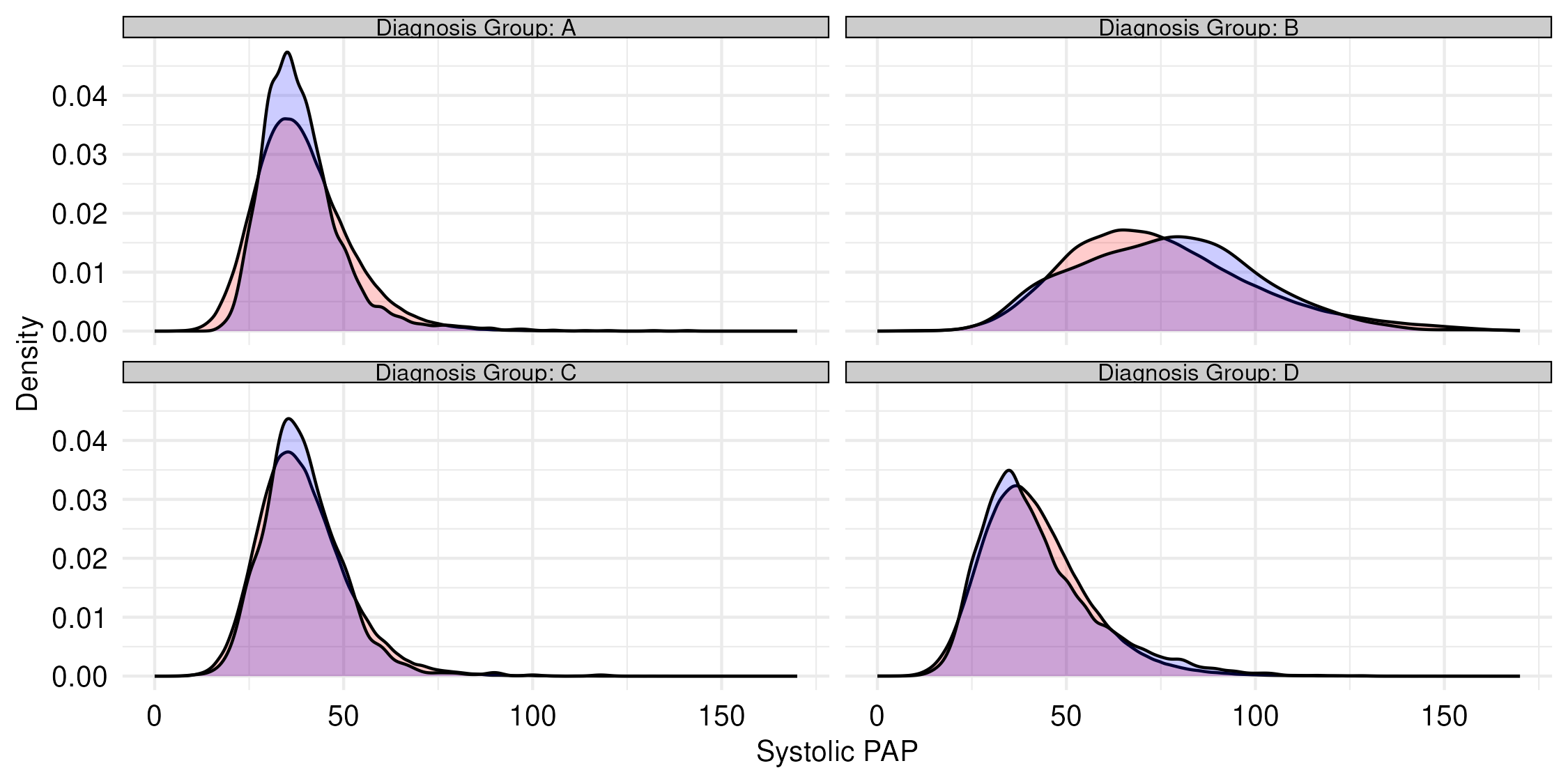

Supplement: S24 Fig — In each, the original SRTR data is represented with a blue shaded density plot, while the combined output from 100 synthetic populations is represented by a red shaded density plot. (TIF) [file pone.0296839.s025.tif]

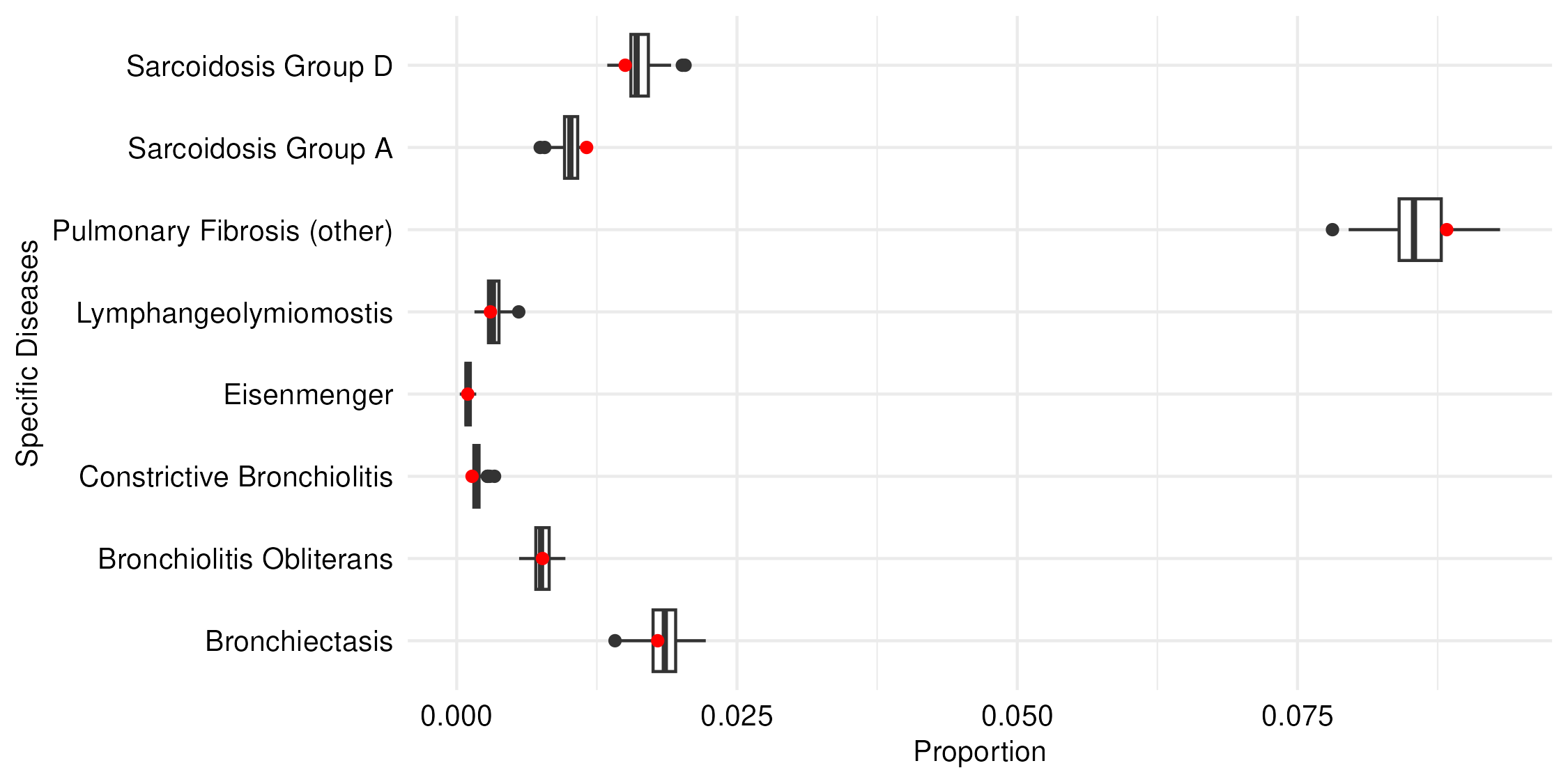

Supplement: S25 Fig — (TIF) [file pone.0296839.s026.tif]

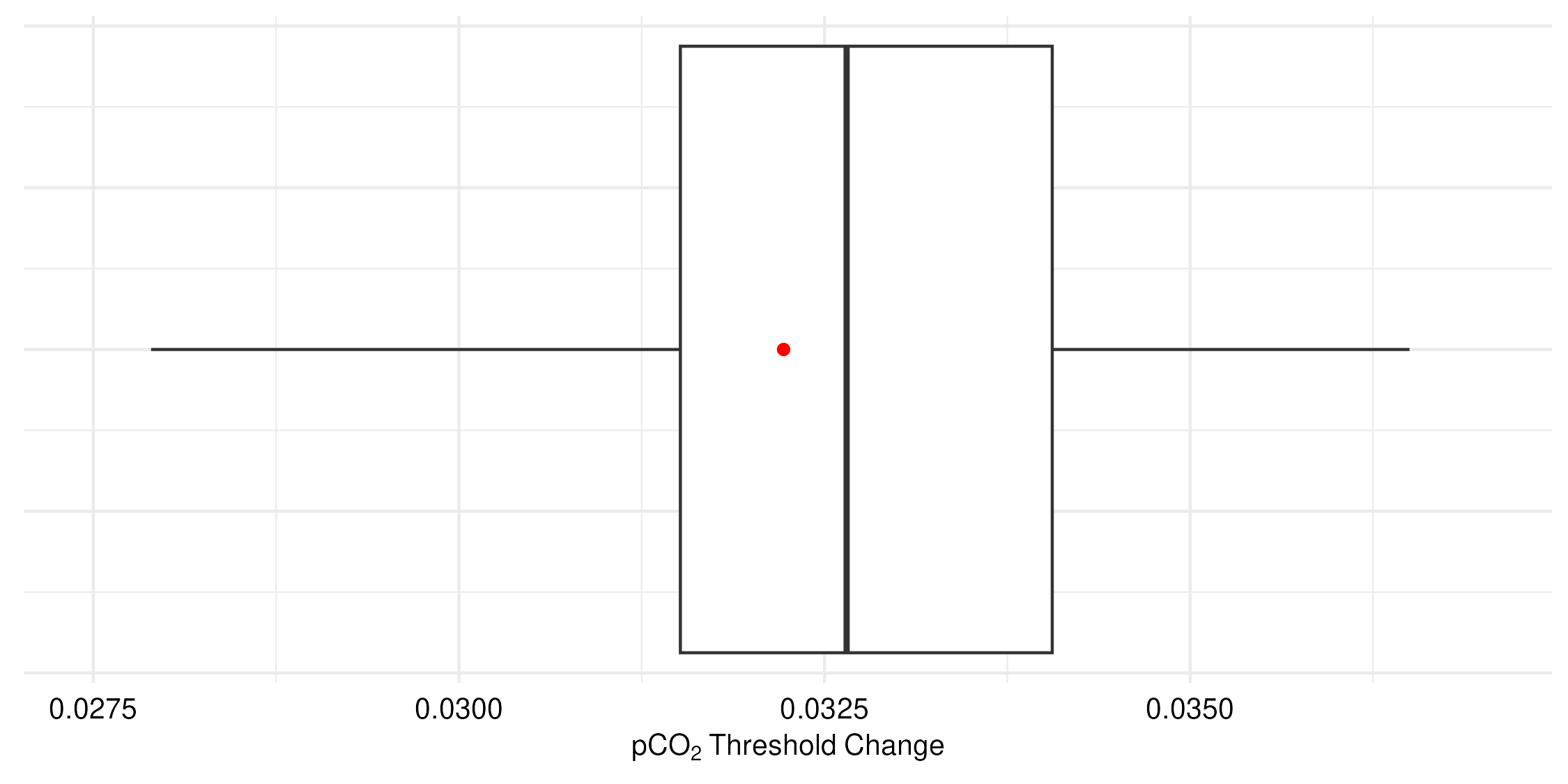

Supplement: S26 Fig — (TIF) [file pone.0296839.s027.tif]

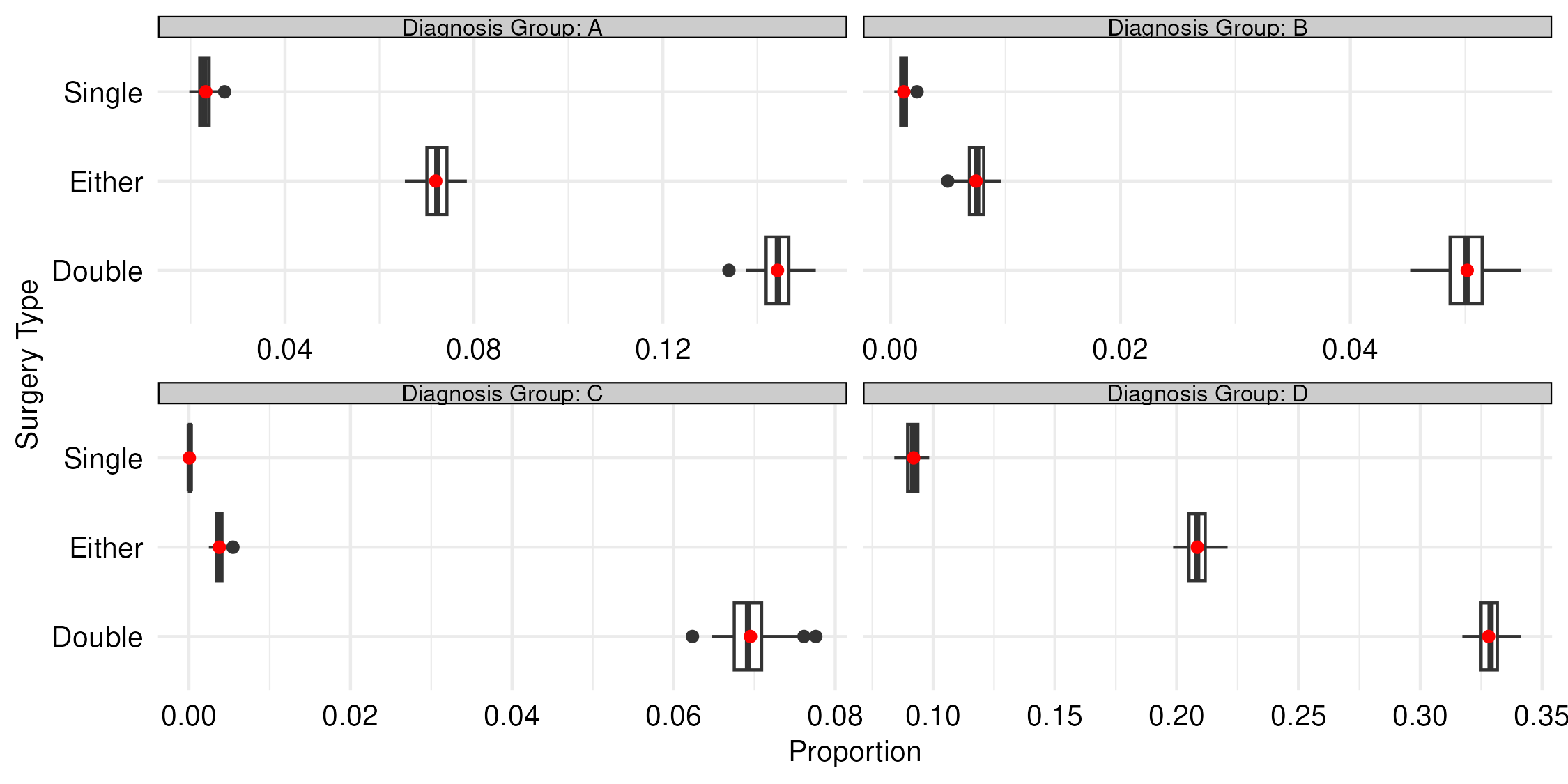

Supplement: S27 Fig — (TIF) [file pone.0296839.s028.tif]

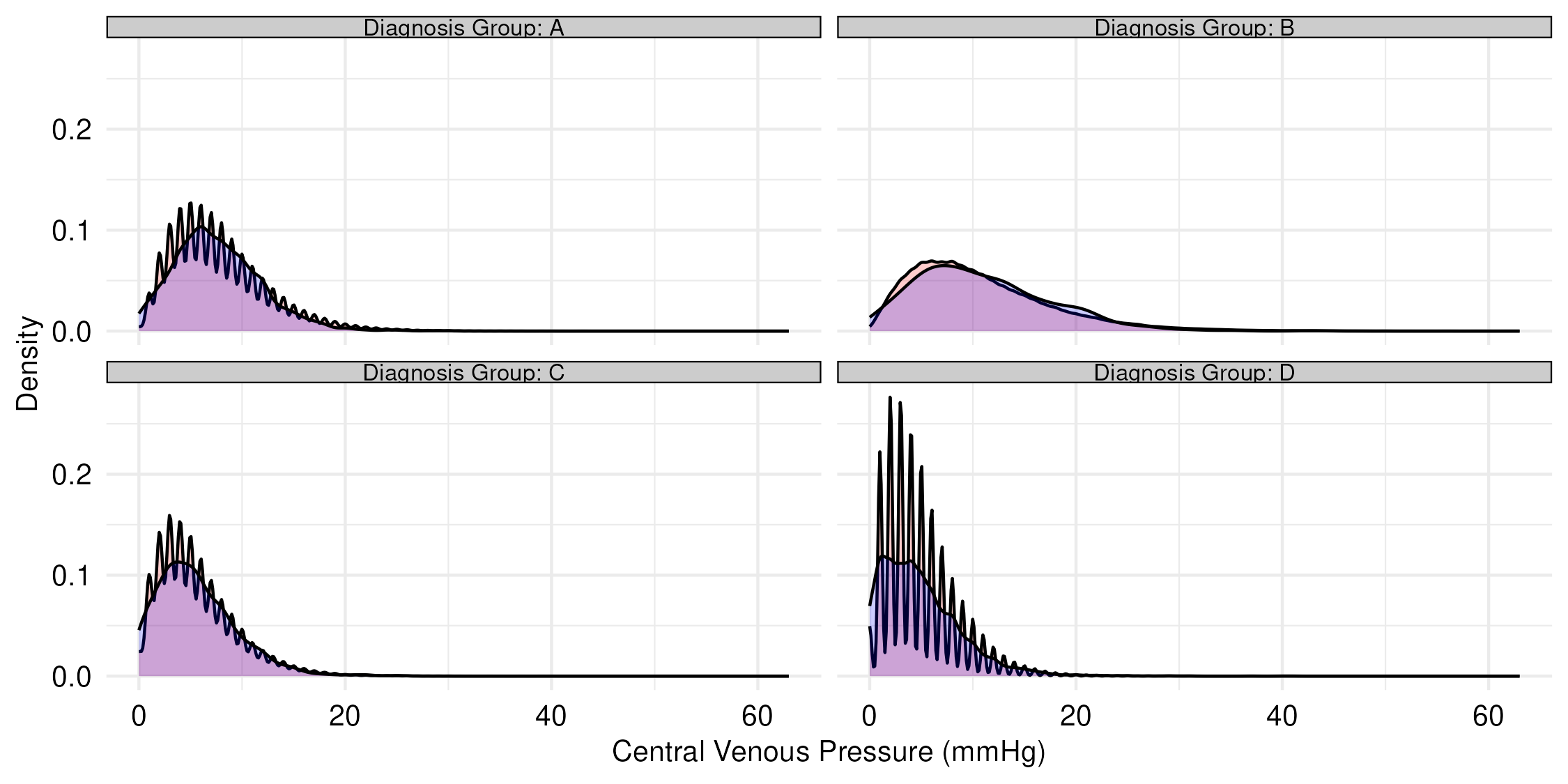

Supplement: S28 Fig — In each, the original SRTR data is represented with a blue shaded density plot, while the combined output from 100 synthetic populations is represented by a red shaded density plot. (TIF) [file pone.0296839.s029.tif]

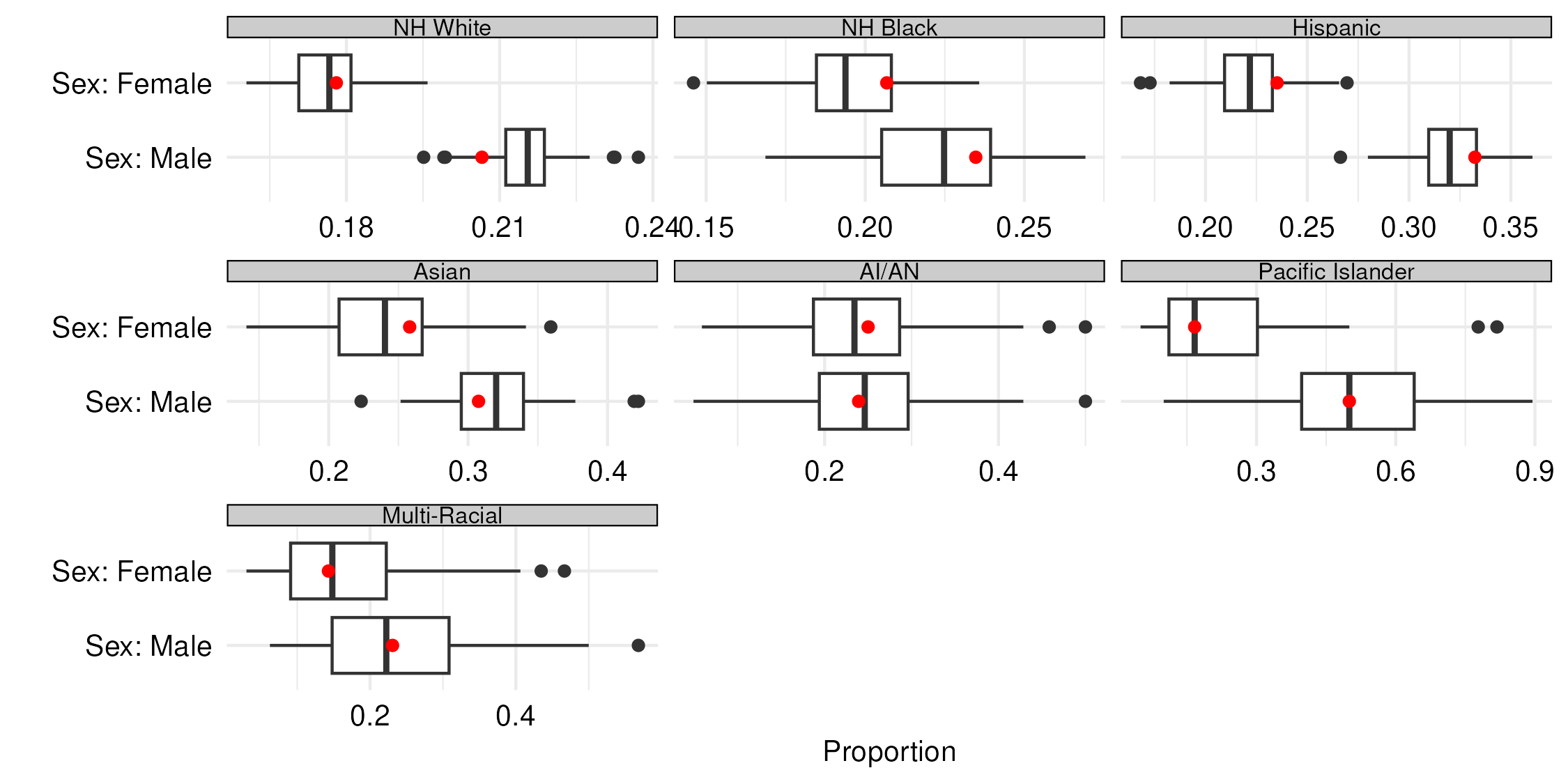

Supplement: S29 Fig — (TIF) [file pone.0296839.s030.tif]

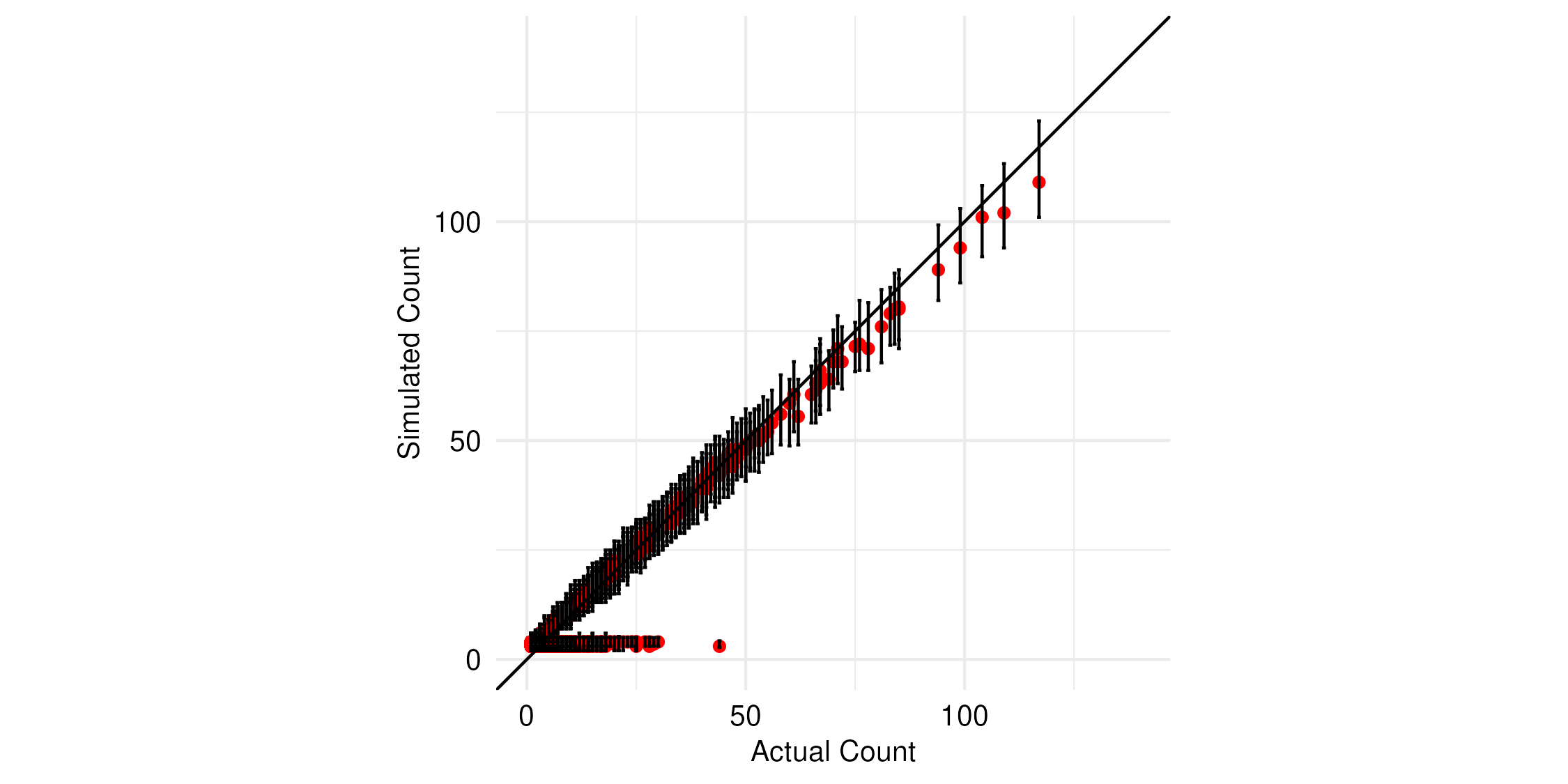

Supplement: S30 Fig — Each red dot represents a donor hospital. The x-axis is the observed count in SRTR from January 1st, 2015 to June 30th 2021. The y-axis is the median simulated count. The vertical bars with each red dot represent the simulated IQR for each donor hospital. (TIF) [file pone.0296839.s031.tif]

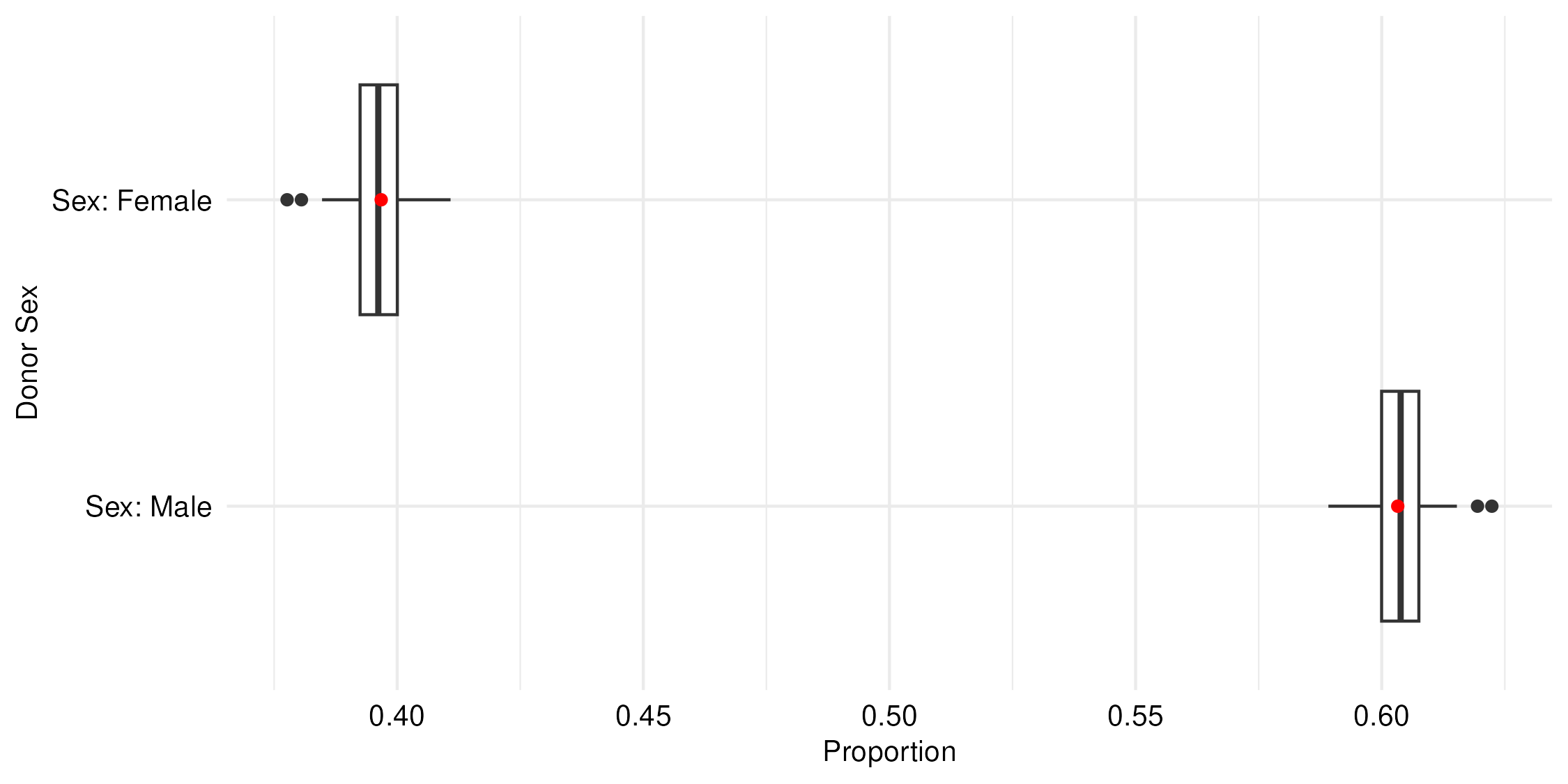

Supplement: S31 Fig — (TIF) [file pone.0296839.s032.tif]

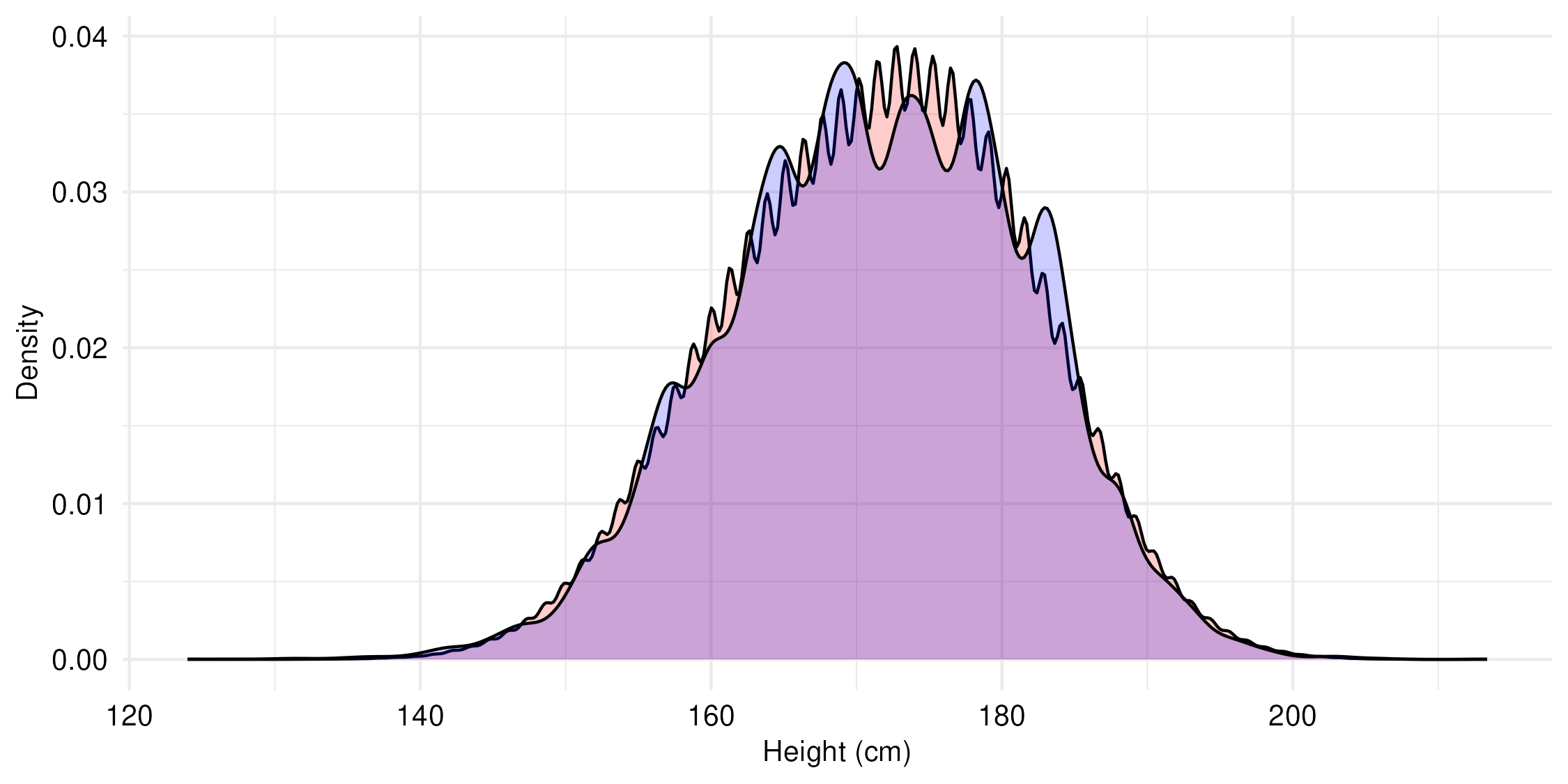

Supplement: S32 Fig — In each, the original SRTR data is represented with a blue shaded density plot, while the combined output from 100 synthetic populations is represented by a red shaded density plot. (TIF) [file pone.0296839.s033.tif]

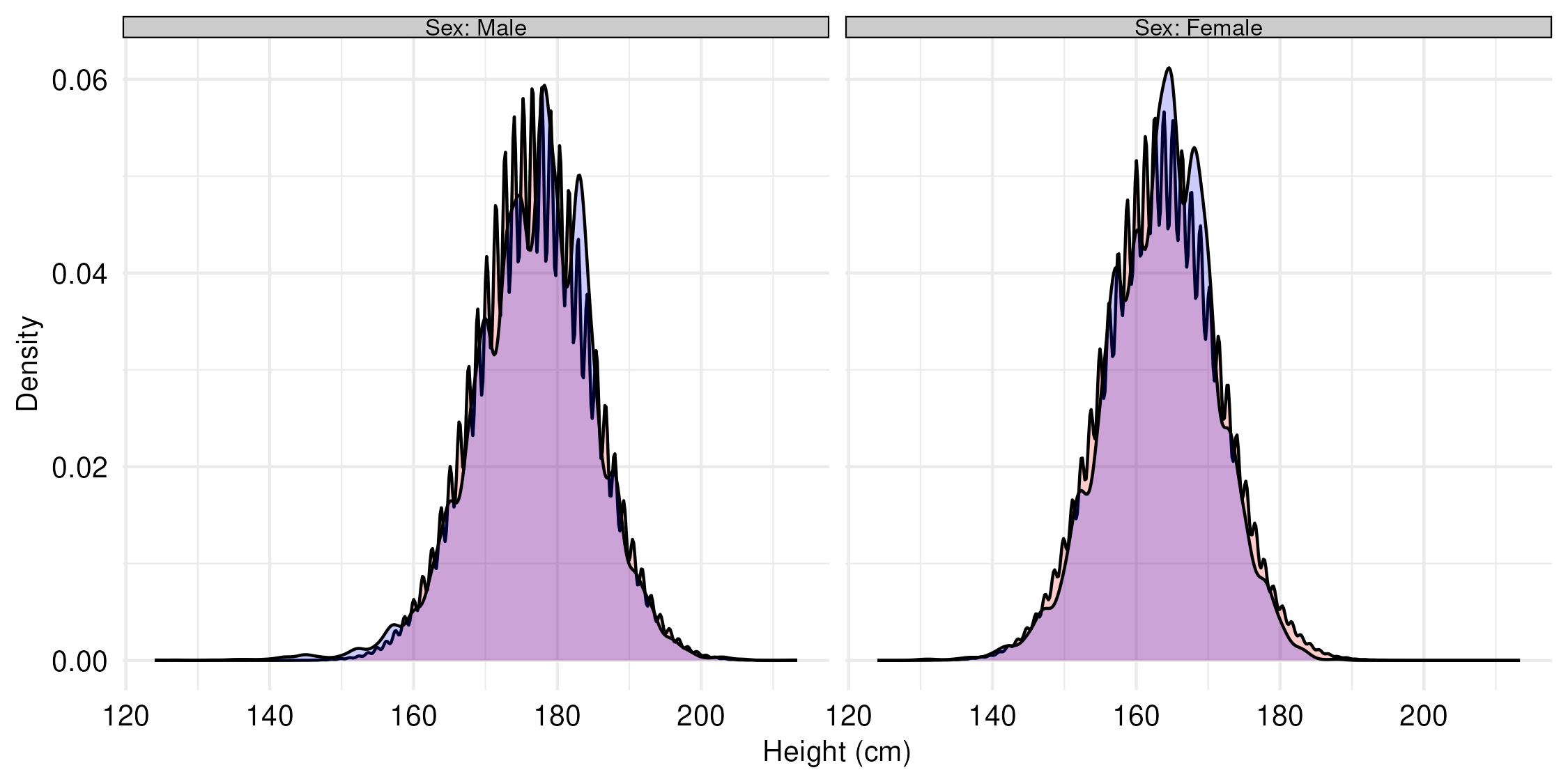

Supplement: S33 Fig — In each, the original SRTR data is represented with a blue shaded density plot, while the combined output from 100 synthetic populations is represented by a red shaded density plot. (TIF) [file pone.0296839.s034.tif]

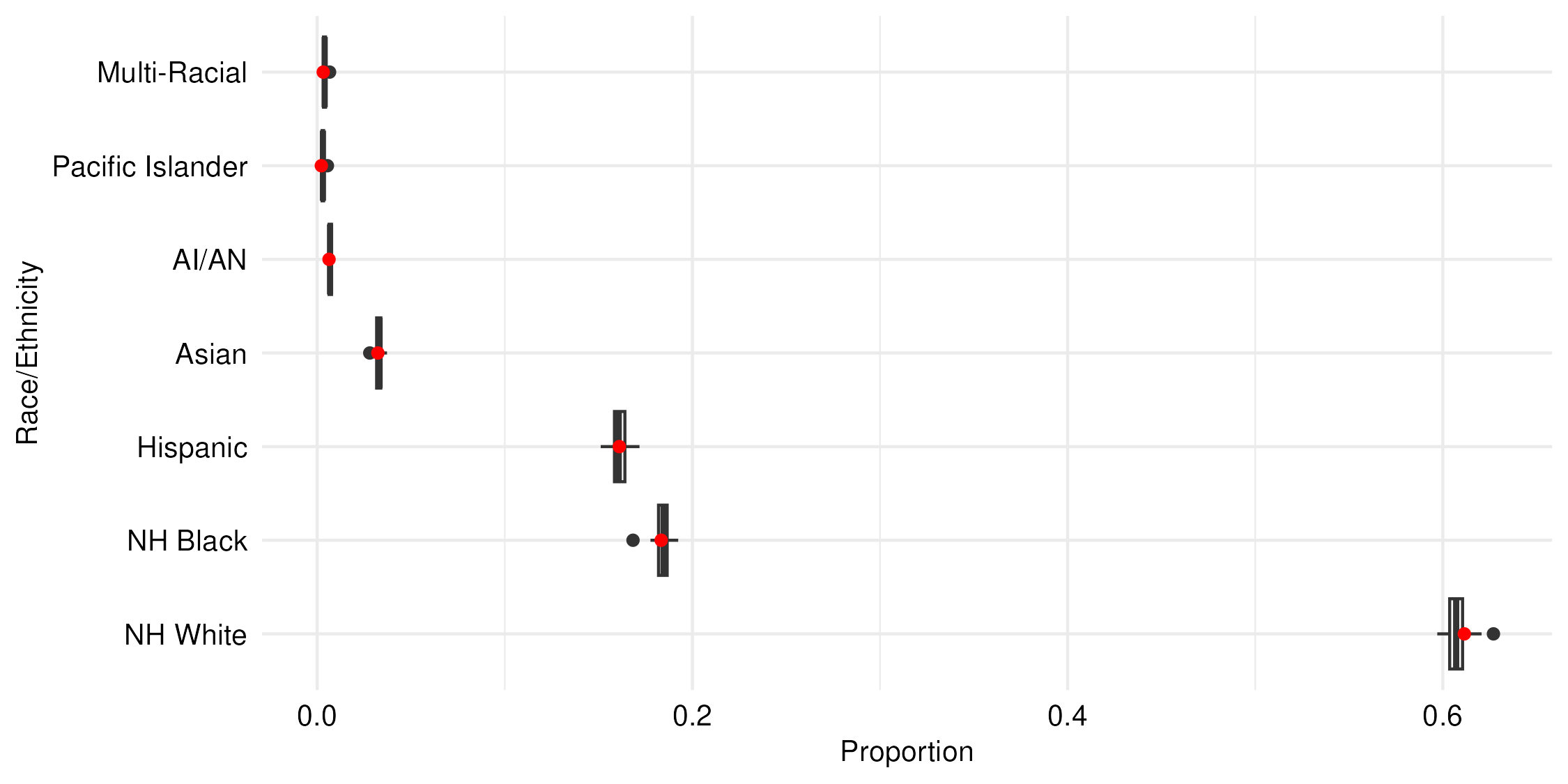

Supplement: S34 Fig — (TIF) [file pone.0296839.s035.tif]

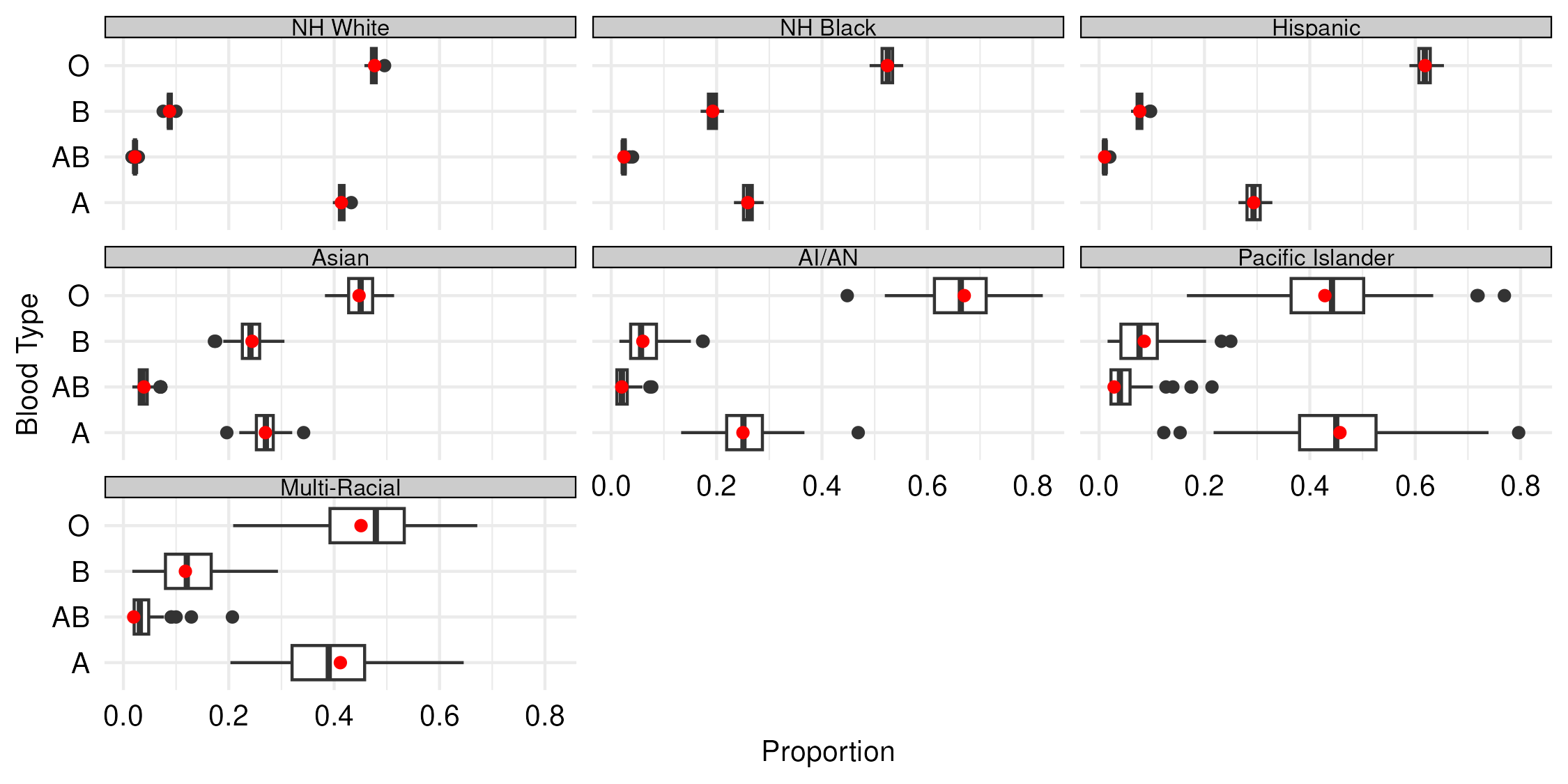

Supplement: S35 Fig — (TIF) [file pone.0296839.s036.tif]

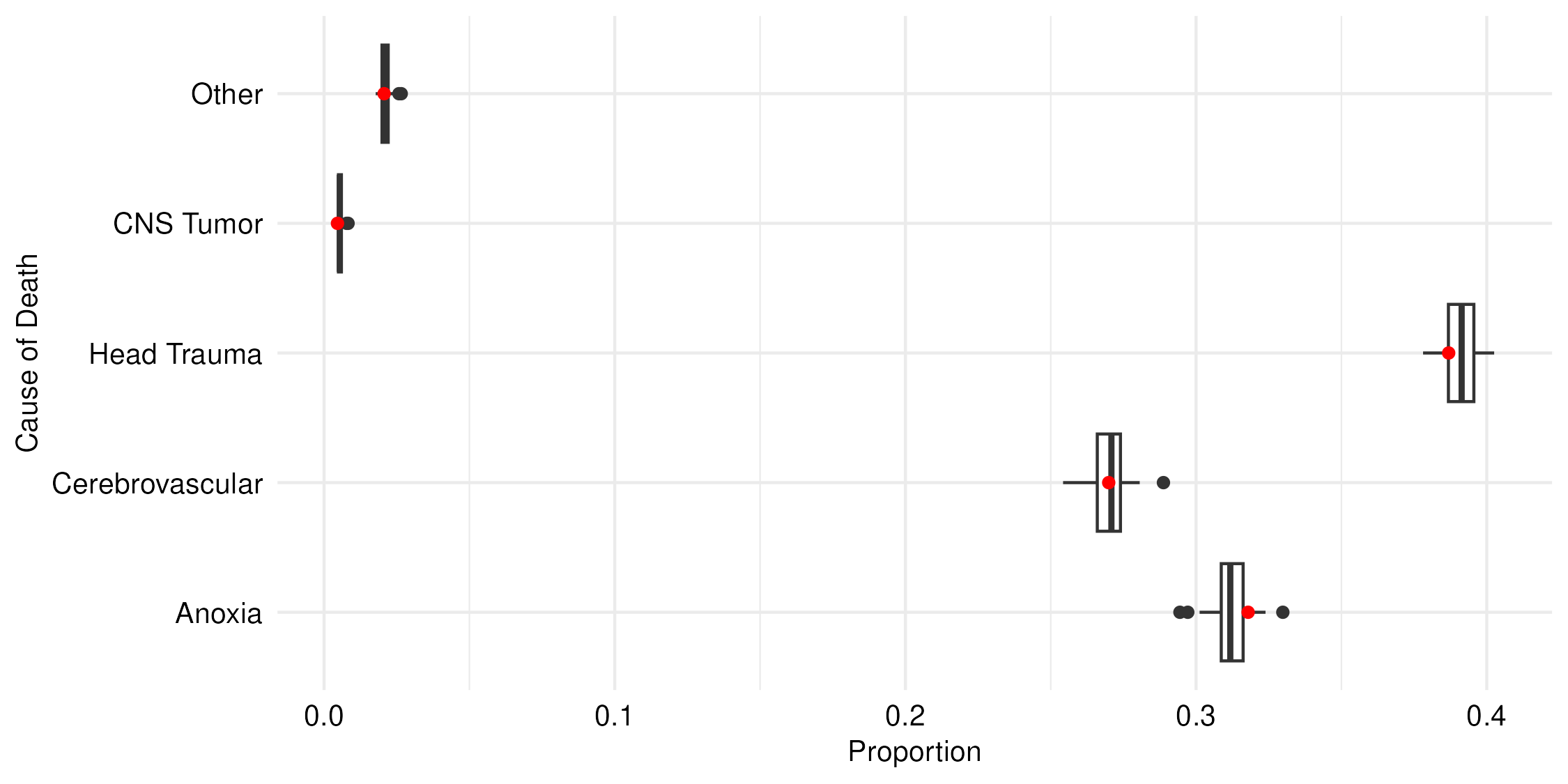

Supplement: S36 Fig — (TIF) [file pone.0296839.s037.tif]

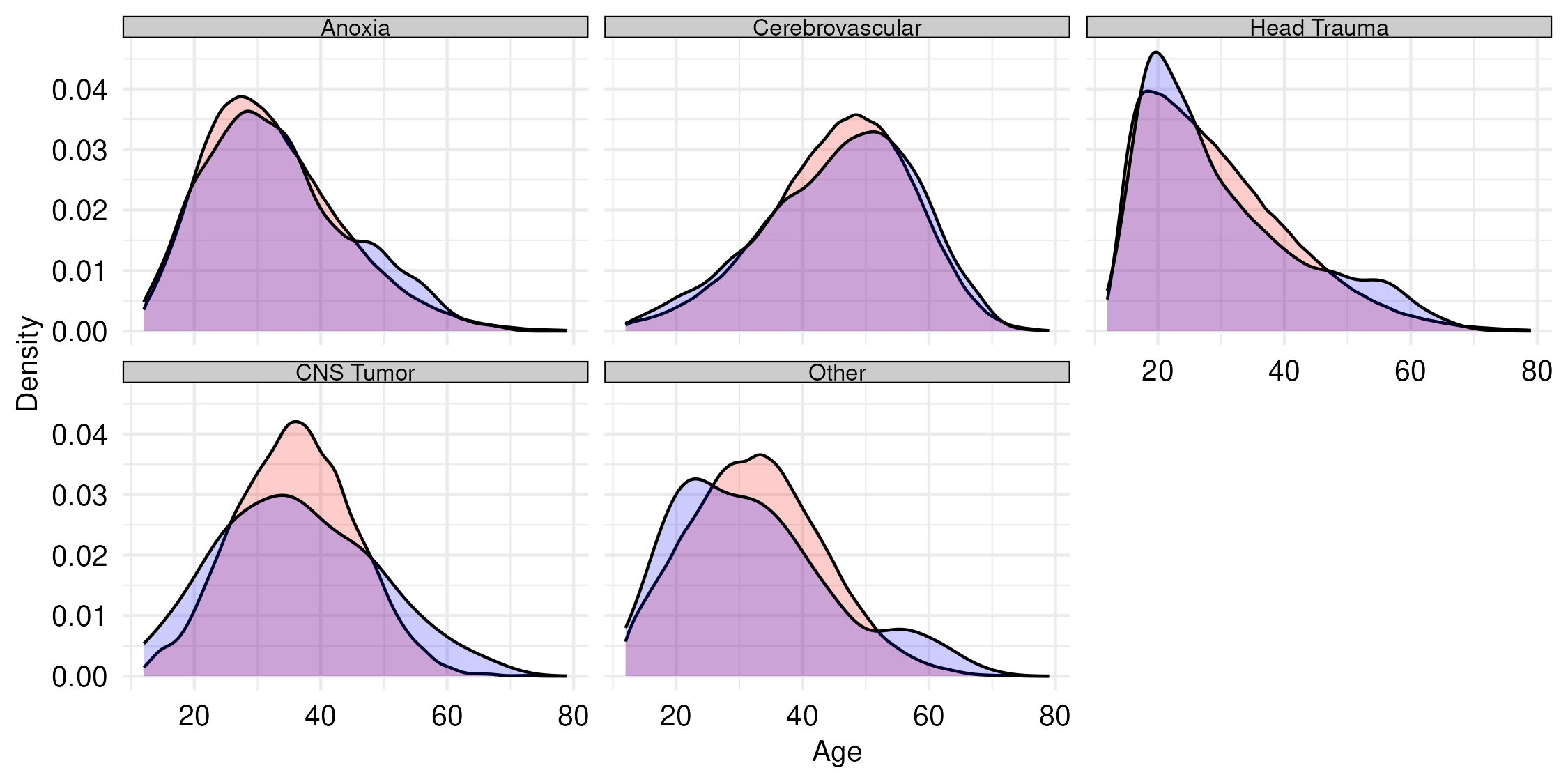

Supplement: S37 Fig — In each, the original SRTR data is represented with a blue shaded density plot, while the combined output from 100 synthetic populations is represented by a red shaded density plot. (TIF) [file pone.0296839.s038.tif]

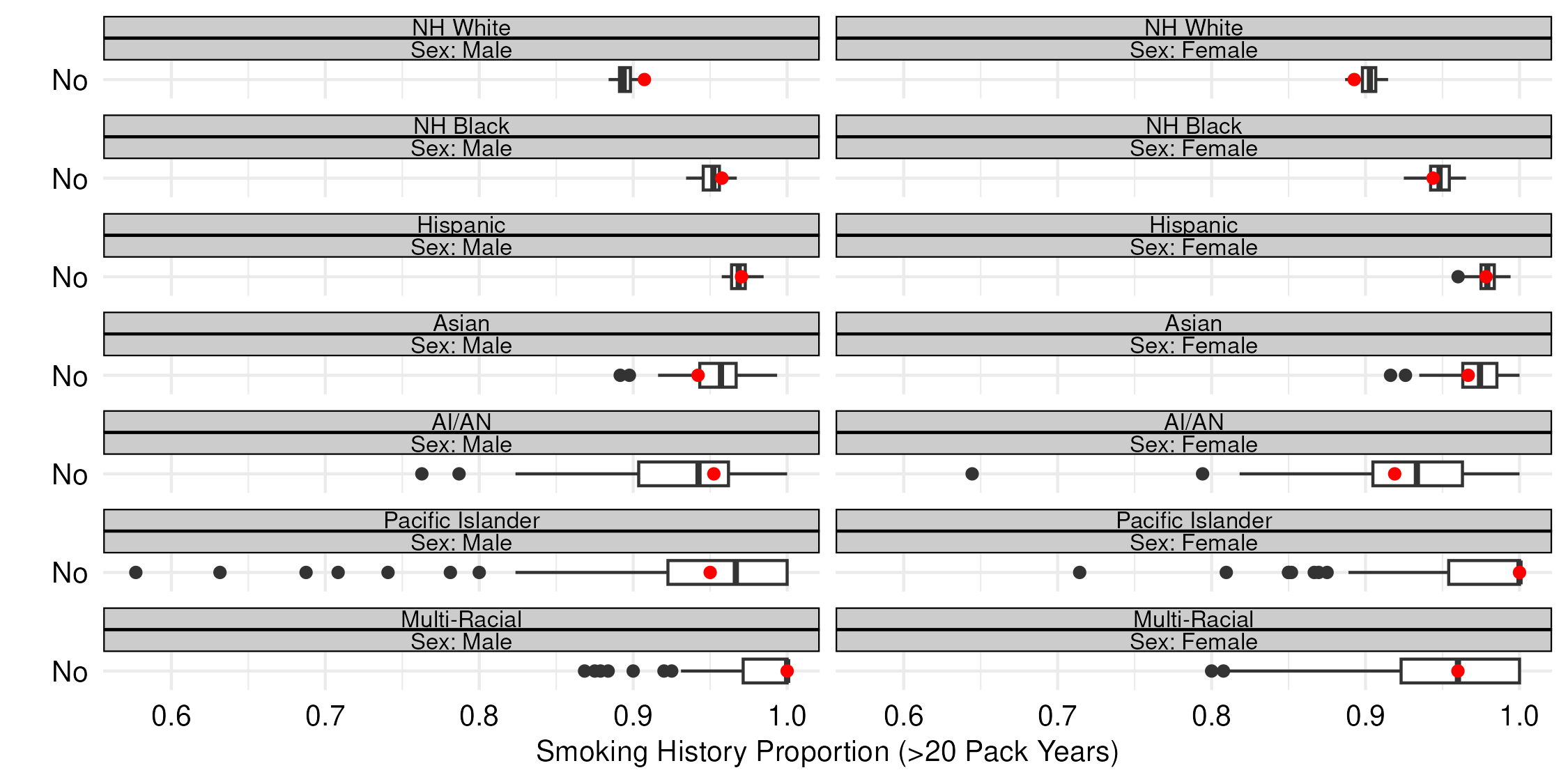

Supplement: S38 Fig — (TIF) [file pone.0296839.s039.tif]

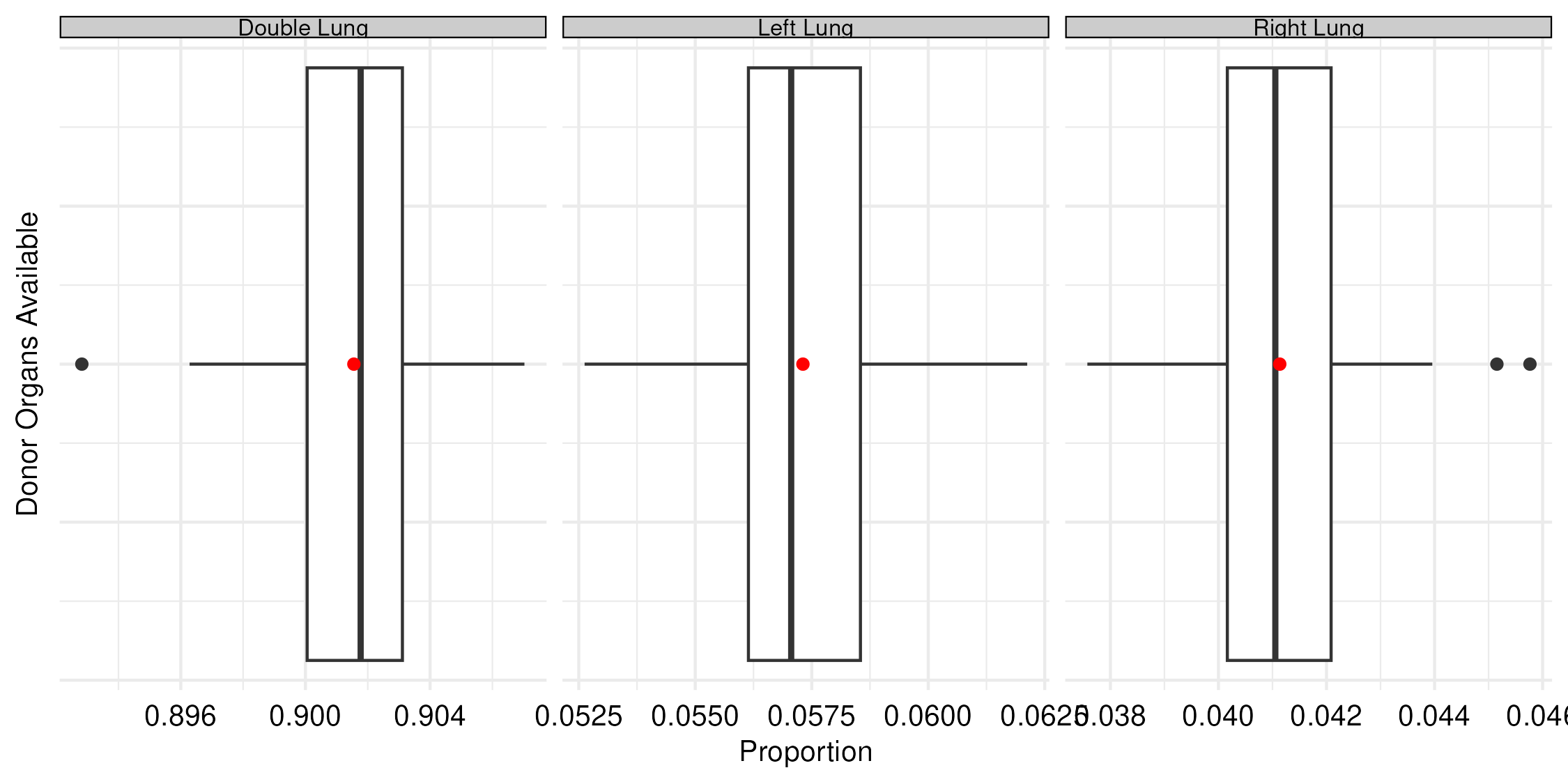

Supplement: S39 Fig — (TIF) [file pone.0296839.s040.tif]

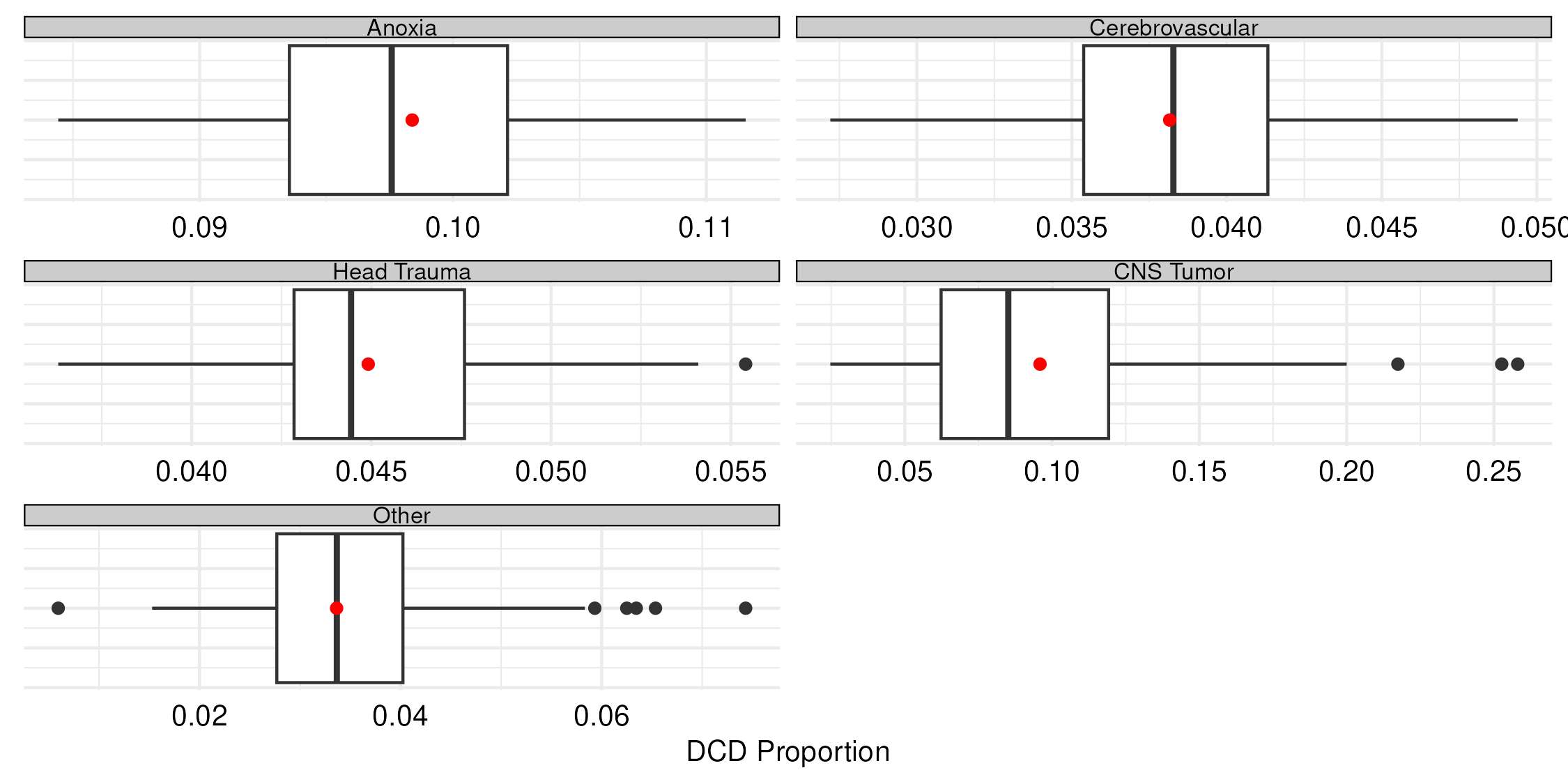

Supplement: S40 Fig — (TIF) [file pone.0296839.s041.tif]

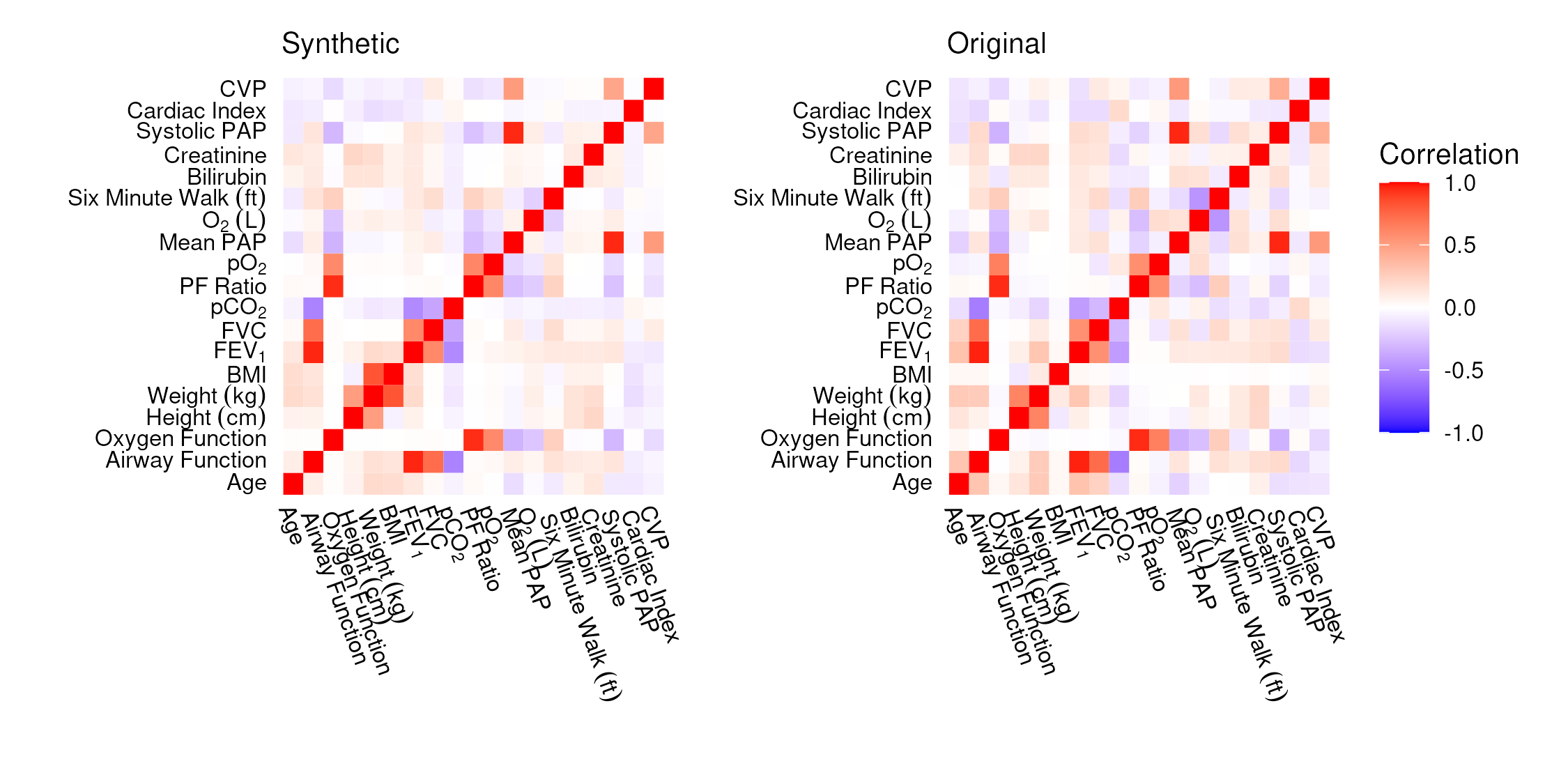

Supplement: S41 Fig — The panel on the left represents correlations from 100 synthetic candidate populations, while the panel on the right summarizes the corresponding correlations from the original candidate cohort from February 19, 2015 through September 1, 2021. Pairwise complete cases were used for the original population in the event of missing data. One outlier of BMI was removed the correlation calculations involving BMI but was included in the calculations for the other variables. (TIF) [file pone.0296839.s042.tif]

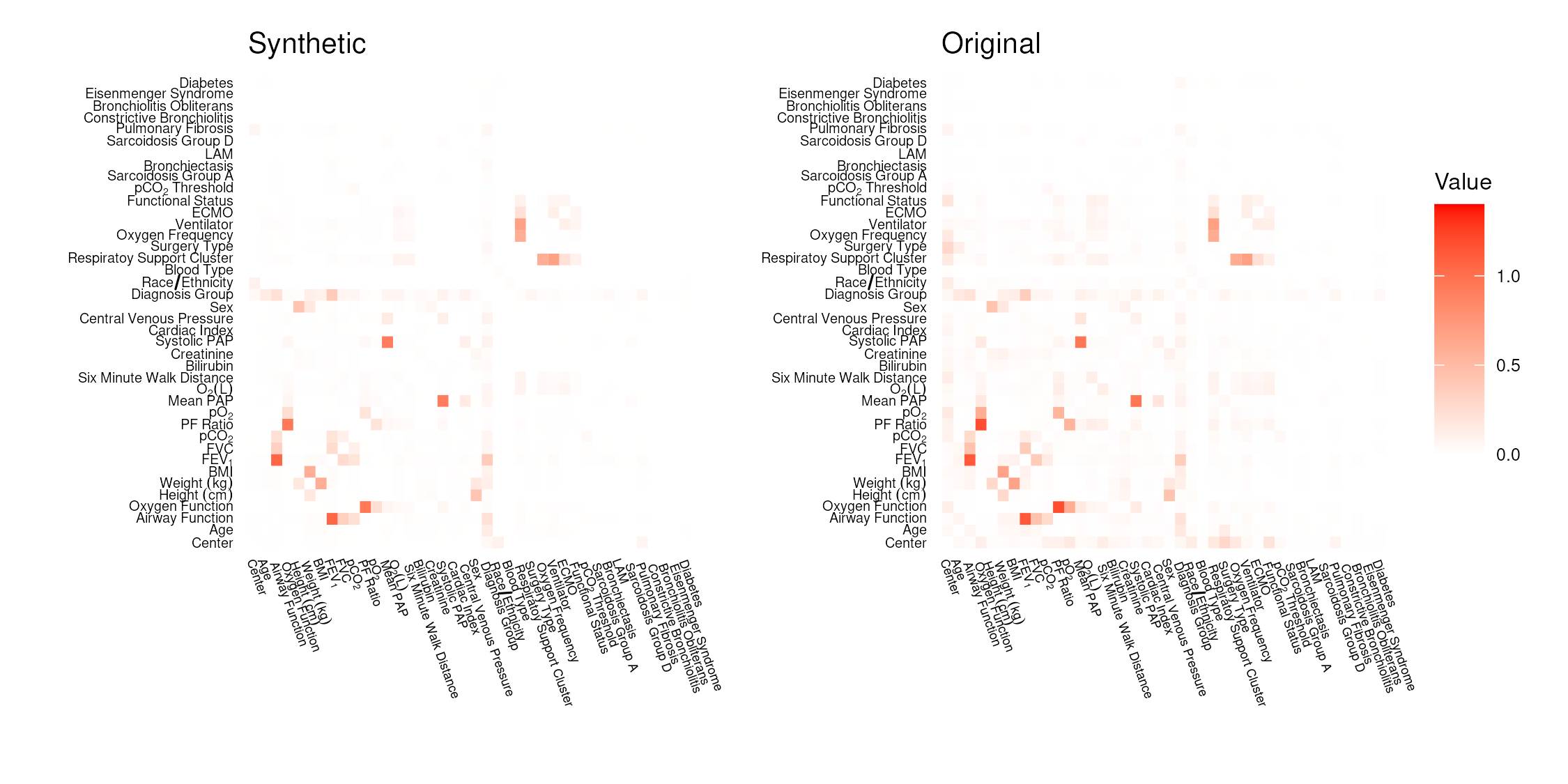

Supplement: S42 Fig — The panel on the left represents the synthetically generated candidates across 100 populations, while the panel on the right represents the original candidate cohort from January 1, 2015 to June 30, 2021. Pairwise complete cases were used for the original population in the event of missing data. To calculate joint entropy with continuous variables, all continuous variables were best split into 4 groups of equal size based on the original data, and these same numerical splits were applied to the synthetic data. (TIF) [file pone.0296839.s043.tif]

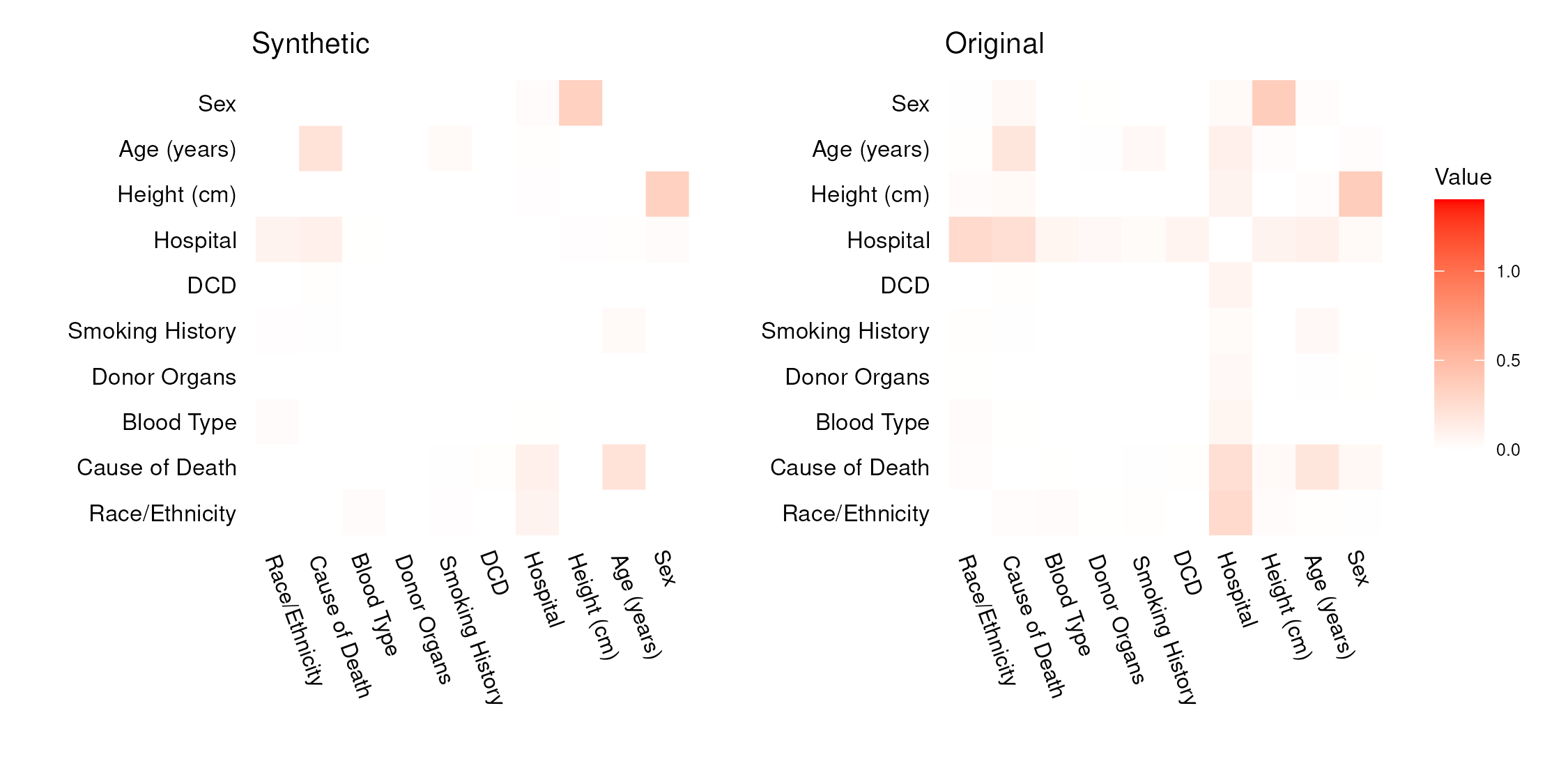

Supplement: S43 Fig — The panel on the left represents the synthetically generated candidates across 100 populations, while the panel on the right represents the original candidate cohort from February 19, 2015 and September 1, 2021. Pairwise complete cases were used for the original population in the event of missing data. To calculate joint entropy with continuous variables, all continuous variables were split into 4 groups of equal size based on the original data, and these same numerical splits were applied to the synthetic data. (TIF) [file pone.0296839.s044.tif]

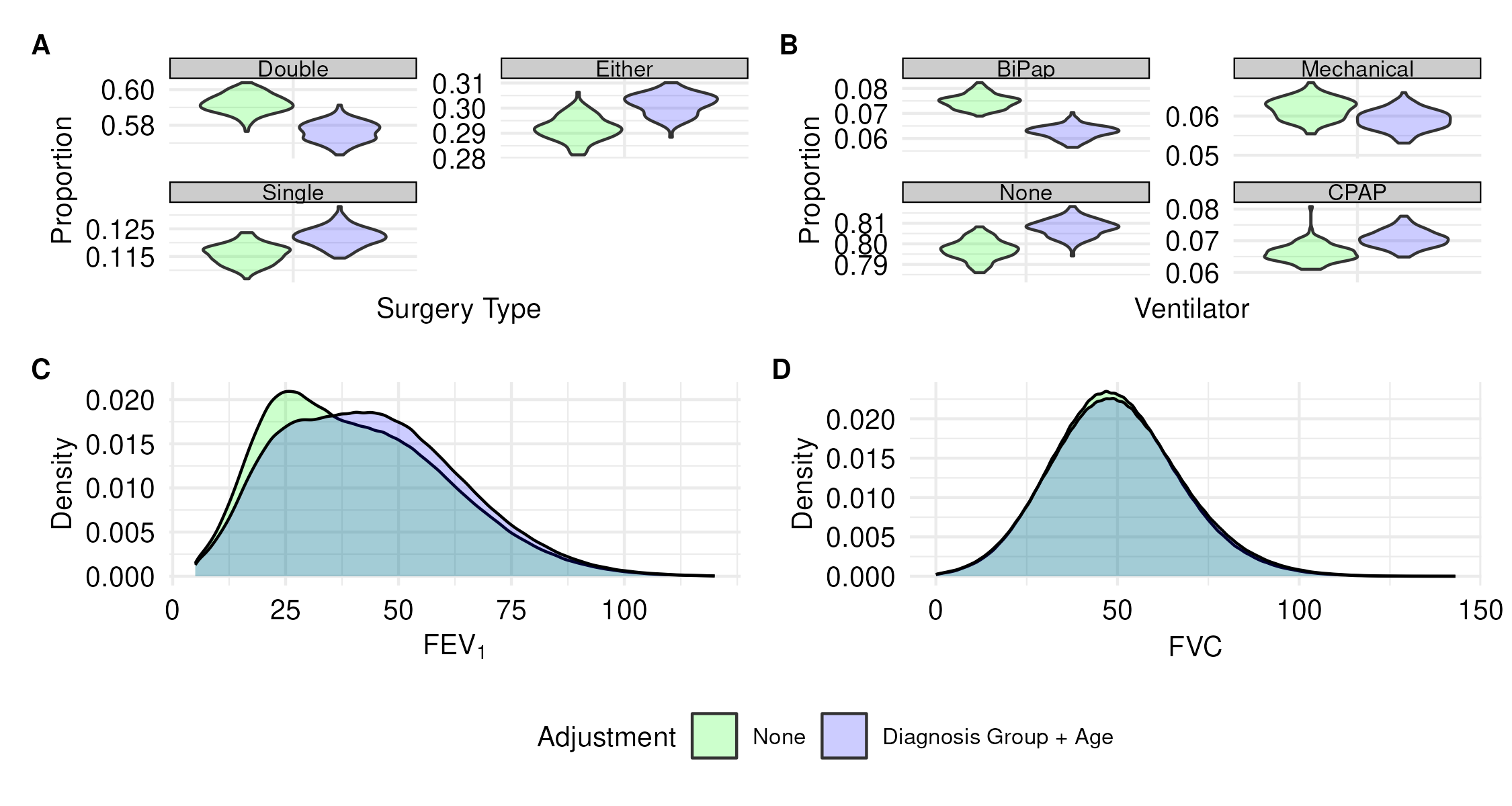

Supplement: S44 Fig — Panels A and B are violin plots comparing the distribution of candidate surgery types and ventilator status respectively from 100 synthetic populations with the original parameters and the diagnosis group and age extrapolations. Panels C and D are density plots comparing the distribution of FEV1 and FVC respectively for 100 synthetic populations with the original parameters and the diagnosis group and age extrapolations. (TIF) [file pone.0296839.s045.tif]
