# Supplementary material for: Creating synthetic populations in transplantation: A Bayesian approach enabling simulation without registry re-sampling
Source: PLoS One. 2024 Mar 21;19(3):e0296839. doi: 10.1371/journal.pone.0296839 (PMC10956776; doi:10.1371/journal.pone.0296839)
Supplement: S1 Table — (DOCX) [file pone.0296839.s046.docx]

**S1 Table –** Comparison of candidate population composition under two scenarios

| Characteristic | Base Case *  (100 simulations) | Scenario with extrapolated diagnosis group & age group trends *  (100 simulations) |
| --- | --- | --- |
|  | **N = 1,828,605 simulated candidates** | **N = 1,896,209 simulated candidates** |
|  | **Mean n per simulation = 18,286** | **Mean n per simulation = 18,962** |
| Sex |  |  |
| Female | 42.0% | 41.7% |
| Male | 58.0% | 58.3% |
| Diagnosis Group |  |  |
| A | 23.9% | 19.4% |
| B | 5.9% | 8.8% |
| C | 7.3% | 2.2% |
| D | 62.9% | 69.7% |
| Race/Ethnicity |  |  |
| NH White | 75.3% | 75.0% |
| NH Black | 10.2% | 10.3% |
| Hispanic | 10.4% | 10.6% |
| Asian | 3.1% | 3.1% |
| AI/AN | 0.5% | 0.5% |
| Pacific Islander | 0.2% | 0.2% |
| Multi/Other | 0.4% | 0.4% |
| Age | 60 [51, 66]; (31, 72) | 62 [54, 68]; (38, 73) |
| Airway Function Index | -0.09 [-0.55, 0.47]; (-1.04, 1.40) | 0.01 [-0.46, 0.58]; (-1.00, 1.49) |
| Oxygen Function Index | 0.03 [-0.30, 0.35]; (-1.03, 1.13) | 0.02 [-0.33, 0.36]; (-1.07, 1.15) |
| Blood Type |  |  |
| A | 37.8% | 37.6% |
| AB | 3.7% | 3.7% |
| B | 11.7% | 11.7% |
| O | 46.8% | 47.0% |
| Height (cm) | 170 [161, 177]; (152, 185) | 170 [163, 177]; (152, 185) |
| Weight (kg) | 75 [64, 86]; (48, 101) | 76 [65, 87]; (50, 101) |
| BMI (km/m^2^) | 26.0 [22.7, 29.3]; (18.1, 34.2) | 26.4 [23.2, 29.6]; (18.6, 34.4) |
| Diabetes | 21.2% | 20.1% |
| Respiratory Support Cluster |  |  |
| 1 | 14.7% | 14.1% |
| 2 | 66.8% | 68.3% |
| 3 | 15.1% | 14.2% |
| 4 | 3.4% | 3.4% |
| Surgical Type Preference |  |  |
| Double | 59.2% | 57.6% |
| Either | 29.2% | 30.2% |
| Single | 11.6% | 12.2% |
| FEV_1_ (%) | 39 [26, 54]; (14, 77) | 42 [29, 57]; (15, 79) |
| FVC (%) | 49 [37, 61]; (22, 80) | 49 [37, 61]; (21, 81) |
| pCO_2_ | 45 [39, 52]; (32, 69) | 44 [38, 51]; (32, 68) |
| P/F ratio | 275 [222, 333]; (131, 478) | 274 [219, 335]; (128, 483) |
| pO_2_ | 68 [56, 86]; (44, 146) | 68 [56, 86]; (44, 147) |
| Mean PAP | 25 [20, 33]; (14, 48) | 26 [20, 33]; (14, 50) |
| Supplemental Oxygen | 4 [3, 7]; (2, 26) | 4 [3, 8]; (2, 26) |
| Oxygen Frequency |  |  |
| At Rest | 85.3% | 85.9% |
| At Night | 1.6% | 1.4% |
| While Exercising | 10.3% | 10.0% |
| None | 2.9% | 2.6% |
| Ventilator |  |  |
| Bipap | 7.5% | 6.3% |
| Mechanical | 6.2% | 5.9% |
| None | 79.7% | 80.8% |
| CPAP | 6.6% | 7.1% |
| Six Minute Walk Distance (ft) | 795 [515, 1,059]; (0, 1,434) | 777 [495, 1,041]; (0, 1,418) |
| Bilirubin | 0.47 [0.32, 0.69]; (0.18, 1.19) | 0.48 [0.33, 0.71]; (0.19, 1.23) |
| Creatinine | 0.81 [0.65, 0.99]; (0.49, 1.33) | 0.82 [0.67, 1.01]; (0.50, 1.35) |
| Systolic PAP | 40 [32, 50]; (23, 74) | 41 [32, 51]; (23, 77) |
| Cardiac Index | 2.79 [2.39, 3.27]; (1.90, 4.10) | 2.76 [2.36, 3.23]; (1.88, 4.04) |
| Central Venous Pressure | 5.0 [3.0, 8.0]; (1.0, 14.0) | 5.0 [3.0, 8.0]; (1.0, 14.0) |
| Functional Status |  |  |
| None | 7.8% | 7.8% |
| Some | 82.9% | 82.7% |
| Total | 9.3% | 9.5% |
| ECMO | 3.4% | 3.4% |
| pCO_2_ increase 15% | 3.3% | 2.9% |
| Unknown |  |  |
| Sarcoidosis Group A | 1.0% | 0.8% |
| Bronchiectasis | 1.9% | 1.5% |
| Lymphangioleiomyomatosis | 0.3% | 0.3% |
| Eisenmenger Syndrome | 0.1% | 0.1% |
| Sarcoidosis Group D | 1.6% | 1.8% |
| Pulmonary Fibrosis (other) | 8.6% | 9.6% |
| Constrictive Bronchiolitis | 0.2% | 0.2% |
| Bronchiolitis Obliterans | 0.8% | 0.8% |
| *^*^* %; Median [IQR]; (5%, 95%) | | |

NH = Non-Hispanic; AI/AN = American Indian/Alaska Native; FEV_1_ = Forced Expiratory Volume in 1 second; FVC = Forced Vital Capacity (% predicted); PCO2 = partial pressure of carbon dioxide; P/F ratio = ratio of partial pressure of arterial oxygen (PaO2) to fraction inspired oxygen (FiPO2); PO2 = partial pressure of oxygen; Mean PAP = mean pulmonary arterial pressure; BIPAP = bi-level positive airway pressure; CPAP = continuous positive airway pressure; Systolic PAP = Systolic pulmonary arterial pressure; CVP = central venous pressure; ECMO = extracorporeal membrane oxygenation. Diagnosis Groups = Group A, obstructive lung disease; Group B, pulmonary vascular disease; Group C, cystic fibrosis and immunodeficiency disorders; and Group D, restrictive lung diseases
