# Supplementary material for: Creating synthetic populations in transplantation: A Bayesian approach enabling simulation without registry re-sampling
Source: PLoS One. 2024 Mar 21;19(3):e0296839. doi: 10.1371/journal.pone.0296839 (PMC10956776; doi:10.1371/journal.pone.0296839)
Supplement: Attachment — Submitted filename: gunsalus et al - synthetic data generation.pdf [file pone.0296839.s047.pdf]

Referee Report

Creating synthetic populations in transplantation: A Bayesian approach enabling simulation without registry re-sampling

P.R. Gunsalus, J. Rose, C.J. Lehr, M. Valapout, J.E. Dalton

This manuscript proposes a strategy for generating synthetic data in the context of lung transplantation. A hierarchical Poisson model is proposed to obtain patient counts, and a pair of Bayesian network models are constructed to generate the characteristics of the populations of lung donors and lung transplant candidates. The models are fit using SRTR data. Overall, the approach provided seems reasonable, and the empirical comparison generally support the appropriateness of the synthetic data. However, there are a few points that I believe require further investigation and further clarity.

Major Comments:

1. The method represents a sophisticated and complex modeling strategy, and there are a number of statistical modeling considerations that are not described in sufficient detail. The authors should further elaborate on the following issues either in the manuscript or as part of the supplementary document.

   (a) Bayesian modeling requires the specification of prior distributions for each parameter in the model, but this is not discussed. In general, how were priors determined? Disperse normal, uniform, etc? What kind of sensitivity analysis was considered to determine the impact of the prior choice?

   (b) The hierarchical Poisson model used to generate counts is very unclear. For a given site, how are the various rate parameters connected? It appears that there are different rates in each time frame for each diagnostic group, but presumably there is some kind of regression model and/or shrinkage hierarchy that shares information across the various rate parameters. (If there isn't any, then this should be discussed.)

   (c) What kind of validation/model simplification steps were used when determining the model at each node (Tables 1 & 2) or were these fixed a priori? Some high level discussion of the general strategy used is needed.

   (d) It is noted that the model is fit using STAN, but STAN has multiple implementation options. Are you using Hamiltonian sampling, variation inference, something else?

   (e) When generating the synthetic data, is each data generated from a randomly chosen parameter sample (as in the usual posterior predictive distribution) or are data generated from a single set of parameter estimates (such as posterior mean)?

2. Two example cases are considered. In one, data are generated corresponding to the same years that the model was trained on. Additionally, data for 2021–2027 were generated to represent an extrapolation case, but there is no ground truth to compare this to. A more

useful investigation might be to withhold the data from year 2020 (or both 2019 and 2020) when (re-)training the model and compare the synthetic data generated for 2020 to the true withheld data.

3. Results in Tables 3 and 4 demonstrate the synthetic data match the modal behavior of the observed data, in terms of estimating distribution medians and IQRs and class probabilities. However, correctly generating appropriate tail behavior for continuous variables may be just as important. This is especially true if the synthetic data is to be used for analysis the relies heavily on individual-level values such as testing a new donor matching algorithm. Unlike in a resampling approach, the tail behavior of your synthetic data may be highly sensitive to the assumed parametric model. For instance, in many cases, normal distributions will have too light of tails relative to ground truth. This tail behavior should be further investigated, perhaps by comparing the 5% and 95% percentiles (or even 1% and 99%) across the synthetic data to the observed data.

4. Similar to the above concern about tail behavior, correlations between variables should also be further investigated. The Supplementary Figures include a number of cases where characterization of one feature is stratified by another, but this stratification seems to always be by parent variables in the Bayesian network; such correlations are expected to be handled correctly since they are estimated within the model fitting. To help verify that the Bayesian network captures the dependence across all variables, it would be useful to consider the joint behavior of a pair of variables that aren't directly connected in the network, such as (cardiac index, age) or (cardiac index, six min walk). Comparison of scatterplots and/or correlation measures for some representative pairs would be illustrative.

Minor Comments:

1. The authors argue that creating synthetic data using a resampling strategy to be ineffective. It would be helpful to include some explicit comparisons between your synthetic data and a resampling-based data. This is particularly important in terms of investigating the behavior of points #2–4 above since resampling should maintain tail and correlation behavior.

2. The row labels in Table 4 are not aligned correctly.

3. Many of the Supplementary Figures for continuous variable compare the observed density plot to one created by combining all 100 synthetic datasets. But, what is really needed is that the density for a single synthetic data approximately matches the observed data. So, the authors should consider also/instead producing these comparisons for using one or more synthetic data (possibly the five in Tables 3 and 4) without combining.

4. The "Each box horizontally..." statement in the caption for each figure is unnecessary and repetitive. I feel that you should only include it once, if it is needed at all.
