# Supplementary material for: Creating synthetic populations in transplantation: A Bayesian approach enabling simulation without registry re-sampling
Source: PLoS One. 2024 Mar 21;19(3):e0296839. doi: 10.1371/journal.pone.0296839 (PMC10956776; doi:10.1371/journal.pone.0296839)
Supplement: Attachment — Submitted filename: Response to Reviewers_11-21-23.pdf [file pone.0296839.s048.pdf]

Dear Dr. Trivedi,

We thank you for the opportunity to respond to the editorial and reviewer comments on our manuscript entitled “Creating synthetic populations in transplantation: A Bayesian approach enabling simulation without registry re-sampling” (PONE-D-23-30778). We have responded to the comments below and have submitted a marked up copy with revisions and a clean copy.

The reviewers have provided many thoughtful insights, for which we are grateful. We have responded to each with the overarching goal of maintaining clarity for a broad readership and reproducibility. The reviewers posed multiple valid alternative methods which could have been invoked in this work. We have incorporated some of these suggestions in the revised manuscript. In other cases, we have attempted to better articulate our rationale for our original methodologic choices. We hope the reviewers find this acceptable. The work reported here represents the initial versions of open-source synthetic donor and candidate generation models. This open-source approach will allow other users to develop their own alternative versions using differing clinical assumptions or modeling techniques.

Sincerely,

Johnie Rose

Reviewer #1: This manuscript - "Creating synthetic populations in transplantation: A Bayesian approach enabling simulation without registry re-sampling" by Johnie Rose et al - describes a method for generating simulated lung transplant donor and recipient populations, with the goal of being able to use these simulations to predict the impact of demographic shifts or policy changes. The concept is well described, and the manuscript well written. I have a few questions and comments:

C1) As with most models, picking the appropriate variables, and determining the associations between them, determines the utility of the model. On the donor side of the model, was there any consideration to including the paO2/FiO2 ratio for the donor lungs as a variable? This is the single most important clinical variable regarding the likelihood of the lungs being accepted, as well a critical factor in the success of the transplant. As such, knowing how the model expects this parameter to change with shift in demographics would be very important.

R1) The data used to train our donor model reflected only organs which had been procured for transplant and had thus met a minimum threshold of acceptability prior to the decision to transplant. Currently, a comprehensive accounting of organ offer acceptance decision-making is generally lacking in the field. For example, a donor paO2/FiO2 ratio that may have been unacceptable may improve (resulting in acceptance) or not (resulting in decline of an organ offer) after lung recruitment maneuvers. At this time, these steps and the final paO2/FiO2 that ultimately inform the procurement decision are

not consistently captured in the transplant registries. Such knowledge will be vital to determine the appropriate donor factors which should be generated.

C2) Similarly, ex vivo lung perfusion has been an increasing factor in the utilization of donors, especially with the increase in the use of DCD for lung procurement. Knowing if changes will lead to an increase in the need for ex vivo lung perfusion would also be of great clinical import, since not all centers have access to this technology.

R2) We based our selection of the initial set of donor variable generated primarily on the analysis by Mulvihill et al [“Variability in Donor Organ Offer Acceptance and Lung Transplantation Survival” published 2020 in *J Heart Lung Transplant]*. Specifically, we used statistically significant predictors of first-rank offer acceptance from Supplementary Table 1 of Supplemental File #3 of that work. We agree that both paO2/FiO2 ratio and *ex vivo* perfusion could be important additional factors to include in future synthetic donor models. The current work represents a first step in the development of synthetic populations for transplant simulation, but the open-source nature of the work ensures that—in addition to our own future work—the transplant community will be able to develop more comprehensive donor and candidate generation models moving forward as the science around acceptance decision making matures.

C3) I appreciate that the entirety of the network models would be difficult to present, but the fact that you felt the need to develop three indices/clusters to keep certain variables grouped leads one to wonder about the organization of the rest of the model. Can you give some additional description about how the network was organized? You state "presumed dependencies between variables were based on known clinical and physiologic relationships" - which leaves quite a bit to the imagination.

R3) The Airway Function Index (AFI), Oxygen Function Index (OFI), and Respiratory Support Clusters (RSC) applied are based on work our group recently published in the *Journal of Heart and Lung Transplant [*A new method for classifying prognostic risk factors in lung transplant candidates. *J Heart Lung Transplant.* 2023 Nov;42(11):1569-1577*.]* We undertook that work to create clinically meaningful categories to classify the lung function of waitlist candidates. Candidates with similar AFI can be expected to have similar, predictable patterns of FEV1, FVC and pCO2; while candidates with similar OFIs would have similar, predictable patterns of PaO2/FiO2, pO2, and mPAP, and candidates in the same RSC should have similar needs for supplemental oxygen, ventilatory assistance, and ECMO. The three constructs (AFI, OFI, and RSC) validly capture the interrelatedness of a set of key variables relevant to candidate outcomes and therefore are a useful tool to reduce the complexity of the candidate generation model.

We have amended the language around “presumed dependencies” to emphasize that the relationships specified in the network structure were all empirically confirmed using the SRTR data: “*The dependencies represented by the network structures were based on known clinical and physiologic relationships and confirmed empirically within the SRTR data.*” Perhaps more importantly, the validation results we report attest to the fact that the assumptions made about variable interdependencies were sound.

C4) Some of your answer to question #3 will no doubt involve pointing to your simulation data, showing that you accurately simulated the existing data, which is a strong point your favor. I do find some of the discrepancies to be interesting - for example, why do you think the simulated population is shorter than the actual population? You explain away some of the other discrepancies by saying there was incomplete data, but that should not be an issue for height, as neither the donors nor recipients would be able to match without a height entered into the system.

R4) These models are necessarily an attempt to capture patterns in the underlying empirical data. As such, the extent to which parametric distributions reflect the actual shape of empirical distributions will vary. At the recommendation of Reviewer 3, we added data for 5th and 95th percentiles of continuous variables to the median and IQR data already in Tables 3 and 4; the 5th and 95th percentile numbers are now shown in parentheses in the tables. These additions help to understand how the behavior of the distributions matches reality across the full range of each continuous variable (such as height). As seen from the excerpt of Table 3 below, the simulated height IQR's and 5th and 95th percentiles align well with original donor height distribution, which demonstrates a more accurate fit even if the medians do not align perfectly. Considering the range of donor heights expressed by the distributions, we are confident that the simulated donor heights reflect the actual donor population with sufficient accuracy.

Characteristic	Observed	Synthetic Minimum Count	Synthetic First Quantile Count	Synthetic Median Count	Synthetic Third Quantile Count	Synthetic Maximum Count	100 Synthetic Runs
	**N = 15,826***	**N = 15,470***	**N = 15,746***	**N = 15,854***	**N = 15,949***	**N = 16,209***	**N = 1,584,991***
**Height (cm)**	173 [165, 178]; (155, 188)	171 [164, 179]; (155, 188)	171 [164, 179]; (154, 188)	171 [164, 179]; (155, 188)	171 [164, 179]; (155, 188)	171 [164, 179]; (154, 188)	171 [164, 179]; (155, 188)

C5) For your example use case, if I understand it correctly, you simulated a population several years into the future, presuming that there would be a demographic shift away from cystic fibrosis patients - the scope of this shift was based on extrapolating the current demographic shifts? I find it interesting that you also picked a change in age as the other shift for this use case, as shifting away from cystic fibrosis patients (who are generally much younger) will also shift the average age of the transplanted patient towards older. Can you tell which of your changes led to what impacts downstream? And are you able to separate influences of the two demographic shifts on the overall age - and how that age shift impacts the other variables?

R5) Thank you for this comment. We would like to reiterate that we modeled diagnosis group-specific age trends (mean ages of groups A through D were projected to increase by 1.5, 4.6, 0.4, and 1.1 years, respectively). The effects of these age trends would, therefore, be separate from the effect of declining numbers of cystic fibrosis patients on overall age of candidates (combining all groups). We did simulate the two changes (diagnosis group distribution and diagnosis group-specific age trends) incrementally but did not include this in the manuscript for simplicity. We include those figures below. As can be seen, the largest impact on most outcomes stemmed from changes in the distribution of diagnosis group (which, as noted, would itself affect overall age of candidates even if diagnosis group-specific age increases had

not occurred). We are happy to include this figure in the paper if the editors prefer that we report these more granular results.

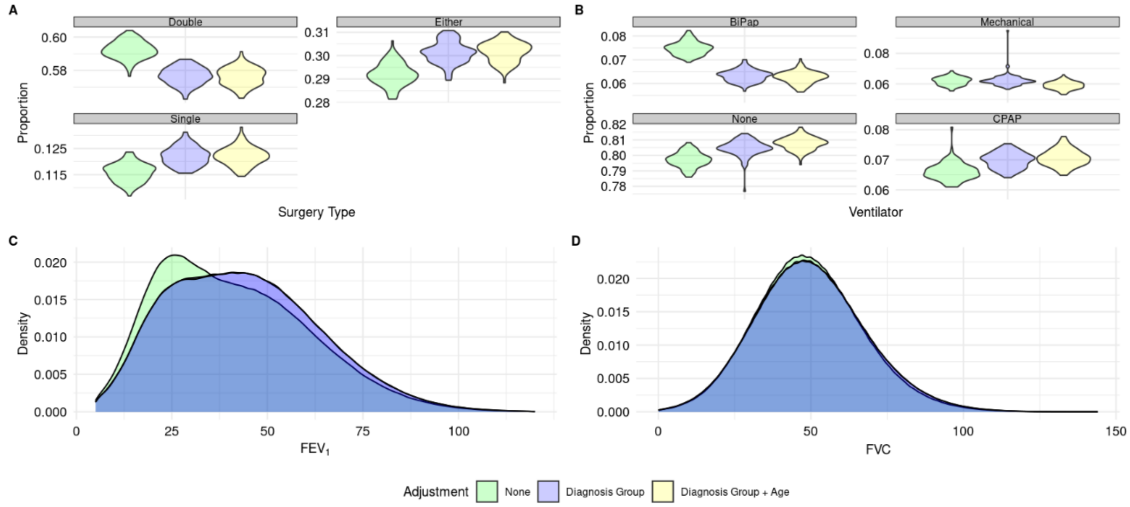

C6) While achieving equity in lung transplant from a racial/ethnic perspective is obviously a laudable goal, I struggle somewhat to understand how this modeling system would facilitate this process. You state that you could "adjust populations in simulations in a manner that acknowledges and attempts to neutralize these disparities" - what does this mean, exactly? How would you adjust the population? If you just increase, for example, the number of African-American recipients, how do you know that the model is going to respond correctly, given that the model is based, and tested, on a population that is predominantly caucasian?

R6) Though likely underrepresented, there was still a substantial number of Non-Hispanic (NH) Black candidates in the SRTR data used (nearly 1,900). The Reviewer is absolutely correct, though, that simply adjusting the racial/ethnic proportions of transplant candidates to reflect racial/ethnic distributions of disease burden would not suffice to correct biases. One would need to know, or at least hypothesize about, how the group of NH Black candidates in the SRTR differed from an ideal, non-biased group of NH Black candidates. An approach to doing so would be to compare various markers of disease severity or non-racial/-ethnic demographic factors between NH White and NH Black patients, then adjust candidate model coefficients to reflect the characteristics that a more equitably selected group of NH Black candidates "should" have. We added the following sentence in the fourth paragraph of the Discussion, where this idea is mentioned: "*For example, one could adjust model coefficients affecting both the racial/ethnic distribution of candidates and markers of disease severity in a manner that one had reason to believe more accurately reflected the true burden of disease in an underserved racial/ethnic group.*"

Reviewer #2: The manuscript addresses an interesting topic. Synthetic control methods are widely used in the medical statistics and economics literature. Novel methods are always welcomed, and the proposal is promising. Numerical examples are given to show the goodness of performance of the proposed method. Some comments follow.

C7). I feel that there is room left to improve the description of the methodological part. The implementation of the method, as well as statistical inference should be described more in depth. Details about the hierarchical Bayesian Poisson model are currently swept under the carpet. Info about the priors and other computational details should be included to ensure the reproducibility of the results. From a methodological perspective, I am wondering if large sample inferential techniques, known to be not well suited to comparative case studies when the number of units in the comparison group is small, are still valid; in general, uncertainty measures should be included and discussed. Similarly, more details about computational times, and the employed algorithm should be introduced.

R7) We appreciate and agree with the reviewer's perspective and have added two additional supplemental tables reporting the priors used (Tables A1.1 and A1.2). We were less concerned about overfitting because the data that we fit represents the entire census of lung transplant data in the United States. We used uninformative priors and, in select cases, the priors were adjusted to convergence and goodness-of-fit measures.

C8) A comparison with other well-established approaches is currently missing. To really appreciate the advantages of the proposal with respect to existing methods, comparisons are mandatory. I am not sure that it is straightforward to compare your approach with that in Abadie A, Diamond A, Hainmueller J (2010). "Synthetic Control Methods for Comparative Case Studies: Estimating the Effect of California's Tobacco Control Program." Journal of the American Statistical Association, 105(490), 493–505. (implemented in the Synth R package), but this is of course a good reference to start from.

R8) We appreciate the comment, but we want to be clear that this work is not intended as a statistical methods paper. We agree that it is critical to benchmark models against well-established approaches when they are available. In our case, the benchmark we chose was of a clinical nature in the form of the SRTR data. The current state of the art for transplant simulation modeling is to simply resample this registry data.

C9) Model validation is crucial. The impact of atypical values, missingness, etc should be discussed and solutions to these potential issues discussed. Similarly, did you check for the reliability of the Poisson assumption? Why did not you consider e.g. a Negative Binomial or another count distribution? Is there any difficulties in moving out from the Poisson case?

R9) We thank the reviewer for this comment. Impacts of atypical values and missingness of data are always a concern and should be considered when fitting parametric models. We chose to include all data except in cases where these outliers affected model fit or posed convergence issues. For example, when modeling the weight distribution for diagnosis group C patients, we excluded the records of diagnosis group C patients with the top and bottom 1% of weight values (see Table 2).

Because synthetically generated data is based on the data that is non-missing in the SRTR, one would not be able to assess whether imputing missing values in the underlying SRTR data improved the fit of synthetic data or not.
We acknowledge there are many alternatives to the Poisson model for count data, but we picked this familiar one. To assess the suitability of the Poisson assumption, we checked the mean to variance ratio per diagnosis group per center. The vast majority were close to 1, with 70% being between 0.8 and 1.2 and 91% between 0.7 and 1.3.

Reviewer #3) This manuscript proposes a strategy for generating synthetic data in the context of lung transplantation. A hierarchical Poisson model is proposed to obtain patient counts, and a pair of Bayesian network models are constructed to generate the characteristics of the populations of lung donors and lung transplant candidates. The models are fit using SRTR data. Overall, the approach provided seems reasonable, and the empirical comparison generally support the appropriateness of the synthetic data. However, there are a few points that I believe require further investigation and further clarity.

Major Comments:
C10). The method represents a sophisticated and complex modeling strategy, and there are a number of statistical modeling considerations that are not described in sufficient detail. The authors should further elaborate on the following issues either in the manuscript or as part of the supplementary document. (a) Bayesian modeling requires the specification of prior distributions for each parameter in the model, but this is not discussed. In general, how were priors determined? Disperse normal, uniform, etc? What kind of sensitivity analysis was considered to determine the impact of the prior choice?

R10) We thank the reviewer for this comment and have reported the final priors in the Appendix in Tables A1.1 and A1.2. All priors began uninformative and were further specified for goodness of fit or model convergence problems.

C11) (b) The hierarchical Poisson model used to generate counts is very unclear. For a given site, how are the various rate parameters connected? It appears that there are different rates in each time frame for each diagnostic group, but presumably there is some kind of regression model and/or shrinkage hierarchy that shares information across the various rate parameters. (If there isn't any, then this should be discussed.)

R11) Donor rate parameters were estimated individually for each donor hospital with greater than two donors between 2013 and 2014. Candidate rate parameters were estimated individually for each of the four candidate diagnosis groups at each center. We have attempted to clarify these explanations under "Step 1: Generation of Site-specific Donor and Candidate Counts" in the Methods section. The priors sampled to generate the model are now described in Tables A1.1 and A1.2,

C12) (c) What kind of validation/model simplification steps were used when determining the model at each node (Tables 1 & 2) or were these fixed a priori? Some high level discussion of the general strategy used is needed.

R12) We chose not to do structural learning. Instead, the dependencies represented by the network structures were fixed a priori based on known clinical and physiologic relationships. Importantly, the validation results we report attest to the fact that the assumptions made about variable interdependencies were reasonable.

C13) (d) It is noted that the model is fit using STAN, but STAN has multiple implementation options. Are you using Hamiltonian sampling, variation inference, something else?

R13) Thank you for this comment, in STAN, we employed the No U-turn Sampler (NUTS) and have added this to the methods.

C14) (e) When generating the synthetic data, is each data generated from a randomly chosen parameter sample (as in the usual posterior predictive distribution) or are data generated from a single set of parameter estimates (such as posterior mean)?

R14) When generating synthetic data, each datum is chosen from a randomly chosen parameter sample from the posterior predictive distribution rather than the posterior mean.

C15) Two example cases are considered. In one, data are generated corresponding to the same years that the model was trained on. Additionally, data for 2021–2027 were generated to represent an extrapolation case, but there is no ground truth to compare this to. A more 1 useful investigation might be to withhold the data from year 2020 (or both 2019 and 2020) when (re-)training the model and compare the synthetic data generated for 2020 to the true withheld data.

R15) We acknowledge that the models were internally validated on SRTR data. Lung-transplantation is a dynamic field in which policy changes rapidly occur and influence structures of candidate and donor populations. This inherently introduces a limitation for any empirical model validation strategies that rely on population stability and identically distributed observations (consider for example COVID-19 related changes in 2020). The initial models for donor and candidate populations were designed to reflect conditional relationships based on clinical knowledge and expertise. They are intended to serve as a basis for further modeling efforts as the field of lung transplantation evolves over time. Nonetheless, given the large sample size and relatively small number of parameters captured in our model we posit that the risk of overfitting to the observed data was relatively minimal.

C16) Results in Tables 3 and 4 demonstrate the synthetic data match the modal behavior of the observed data, in terms of estimating distribution medians and IQRs and class probabilities. However, correctly generating appropriate tail behavior for continuous variables may be just as important. This is especially true if the synthetic data is to be used for analysis the relies heavily on individual-level values such as testing a new donor matching algorithm. Unlike in a resampling approach, the tail behavior of your synthetic data may be highly sensitive to the assumed parametric model. For instance, in many cases, normal distributions will have too light of tails relative to ground truth. This tail behavior should

be further investigated, perhaps by comparing the 5% and 95% percentiles (or even 1% and 99%) across the synthetic data to the observed data.

R16) We would like to thank the reviewer for this astute comment. We have added the 5th and 95th percentiles to Tables 3 and 4 to better show how the tail behavior of our simulated characteristics compares to the tail behavior of the original data. These new results suggest that the tail behavior of the synthetic donor and candidate data closely reflects the underlying empirical data. For consistency, we also added 5th and 95th percentile values to Table A3.1, which compares simulated candidate populations in a base case versus a hypothetical scenario of differing diagnosis group and diagnosis group-specific age distributions.

C17) Similar to the above concern about tail behavior, correlations between variables should also be further investigated. The Supplementary Figures include a number of cases where characterization of one feature is stratified by another, but this stratification seems to always be by parent variables in the Bayesian network; such correlations are expected to be handled correctly since they are estimated within the model fitting. To help verify that the Bayesian network captures the dependence across all variables, it would be useful to consider the joint behavior of a pair of variables that aren't directly connected in the network, such as (cardiac index, age) or (cardiac index, six min walk). Comparison of scatterplots and/or correlation measures for some representative pairs would be illustrative.

R17) We would like to express gratitude for this comment. It has inspired us to add supplemental figures A2.41-43. The first displays correlation coefficients matrices for synthetic and original data for all continuous candidate variables. The second and third depict joint entropy values for all donor and candidate variables, respectively. The first two can be seen below for your ease.

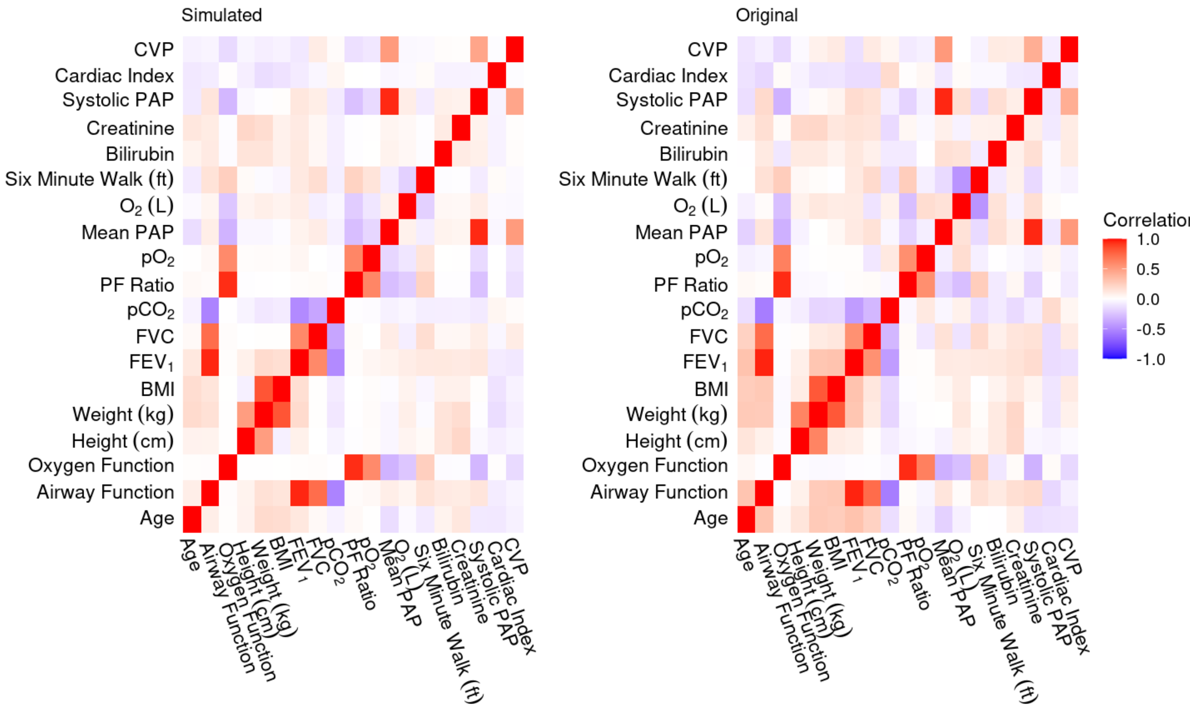

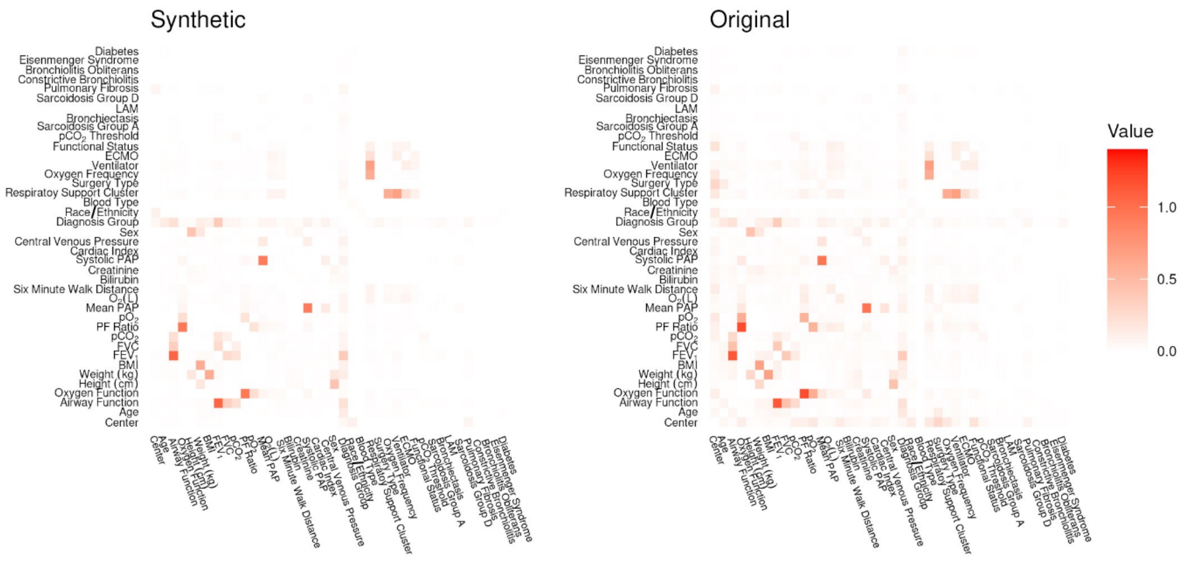

Minor Comments:

C18) The authors argue that creating synthetic data using a resampling strategy to be ineffective. It would be helpful to include some explicit comparisons between your synthetic data and a resampling-based data. This is particularly important in terms of investigating the behavior of points #2–4 above since resampling should maintain tail and correlation behavior.

R18) We do not feel that a resampling strategy is ineffective at reproducing the characteristics of the donor and candidate populations. Instead, we argue in the paper that resampling limits the possibilities for manipulating population parameters to produce populations reflecting ongoing or hypothetical trends, and that relying on resampling can slow progress in simulation because of the data access restrictions necessary to protect patient data confidentiality (see "Synthetic Data" by Raghunathan in *Annual Review of Statistics and Its Application*. 20218:129-140). With this work, our primary goals were to introduce our method of creating synthetic populations and to assess its ability to create realistic populations resembling the underlying SRTR data (including reproducing tail and correlation behavior). Direct comparison of synthetic populations to the full SRTR data seems the most direct method by which to achieve the second goal. If we compared synthetic to resampled populations, any discrepancies between the two may in fact be attributable to random variation in samples.

C19) The row labels in Table 4 are not aligned correctly.

R19) Thank you for noticing this, we have fixed this error.

C20) Many of the Supplementary Figures for continuous variable compare the observed density plot to one created by combining all 100 synthetic datasets. But, what is really needed is that the density for a single synthetic data approximately matches the observed data. So, the authors should consider also/instead producing these comparisons for using one or more synthetic data (possibly the five in Tables 3 and 4) without combining.

R20) We appreciate the reviewer's suggestion here. We agree that showing validity within the individual simulations for each datum is important. We sought to do so with Tables 3 and 4, where we display the results of five individual simulations; we believe that adding 5th/95th percentiles to these tables further strengthens our validation. Below we have attached a figure showing height across the five individual simulation runs included in Tables 3 and 4. To provide a full complement of run-specific figures for five individual simulation runs, though, would require including 160 additional supplemental figures in the Appendix. As previously stated we tried to balance the readability of the paper with the complexity of the analysis.

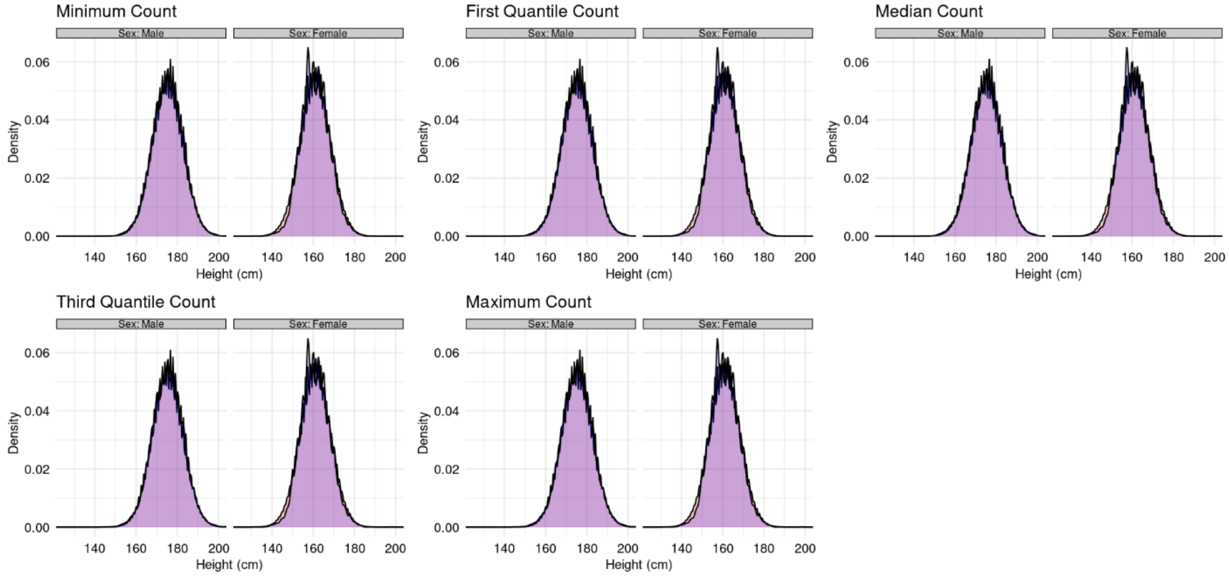

C21) The "Each box horizontally..." statement in the caption for each figure is unnecessary and repetitive. I feel that you should only include it once, if it is needed at all

R21) We thank the reviewer for this comment. We have removed this phrase from the pations of all but Figures 1 and A2.1,
